# Machine learning reveals diverse cell death patterns in lung adenocarcinoma prognosis and therapy

**DOI:** 10.1038/s41698-024-00538-5

**Published:** 2024-02-26

**Authors:** Shun Wang, Ruohuang Wang, Dingtao Hu, Caoxu Zhang, Peng Cao, Jie Huang

**Affiliations:** 1 Department of Respiratory Medicine, Shanghai Xuhui Central Hospital, Zhongshan-Xuhui Hospital, Fudan University, Shanghai, 200031 China; 2 Department of Otolaryngology, the Second Affiliated Hospital of the Naval Military Medical University (Shanghai Changzheng Hospital), Shanghai, 200003 China; 3 Clinical Cancer Institute, Center for Translational Medicine, Naval Medical University, Shanghai, 200433 China; 4 Department of Molecular Diagnostics, The Core Laboratory in Medical Center of Clinical Research, Department of Endocrinology, Shanghai Ninth People’s Hospital, State Key Laboratory of Medical Genomics, Shanghai Jiaotong University School of Medicine, Shanghai, 200011 China; 5 Department of Interventional Pulmonology, Anhui Chest Hospital, Hefei, Anhui 230022 China

**Keywords:** Computational biology and bioinformatics, Prognostic markers

## Abstract

Cancer cell growth, metastasis, and drug resistance pose significant challenges in the management of lung adenocarcinoma (LUAD). However, there is a deficiency in optimal predictive models capable of accurately forecasting patient prognoses and guiding the selection of targeted treatments. Programmed cell death (PCD) pathways play a pivotal role in the development and progression of various cancers, offering potential as prognostic indicators and drug sensitivity markers for LUAD patients. The development and validation of predictive models were conducted by integrating 13 PCD patterns with comprehensive analysis of bulk RNA, single-cell RNA transcriptomics, and pertinent clinicopathological details derived from TCGA-LUAD and six GEO datasets. Utilizing the machine learning algorithms, we identified ten critical differentially expressed genes associated with PCD in LUAD, namely CHEK2, KRT18, RRM2, GAPDH, MMP1, CHRNA5, TMPRSS4, ITGB4, CD79A, and CTLA4. Subsequently, we conducted a programmed cell death index (PCDI) based on these genes across the aforementioned cohorts and integrated this index with relevant clinical features to develop several prognostic nomograms. Furthermore, we observed a significant correlation between the PCDI and immune features in LUAD, including immune cell infiltration and the expression of immune checkpoint molecules. Additionally, we found that patients with a high PCDI score may exhibit resistance to immunotherapy and standard adjuvant chemotherapy regimens; however, they may benefit from other FDA-supported drugs such as docetaxel and dasatinib. In conclusion, the PCDI holds potential as a prognostic signature and can facilitate personalized treatment for LUAD patients.

## Introduction

Lung cancer is widely recognized as the leading cause of cancer-related mortality and the second most prevalent cancer globally, with lung adenocarcinoma (LUAD) being the most common histological type^1–3^. Despite advancements in diagnostic techniques and treatment modalities, the prognosis for patients remains grim, with a discouraging 5-year survival rate of merely 10–20%^4–6^. For patients diagnosed at an advanced stage, the available treatment options are limited to targeted therapy and immunotherapy. However, the high degree of heterogeneity in lung cancer and the inevitable development of drug resistance results in only a small fraction of patients responding favorably to these therapeutic approaches. In recent times, significant progress has been made in the treatment of non-small cell lung cancer, particularly through the use of immunotherapy, specifically immune checkpoint inhibitors such as anti-PD-1/PD-L1^7^. However, the emergence of high resistance rates and low overall response rates poses a significant challenge^8^. Therefore, there is an urgent need for further research into biomarkers that could potentially predict the efficacy of targeted and immune therapies in LUAD. Additionally, exploring the underlying mechanisms will provide potential targets and theoretical foundations for drug design and clinical decision-making.

Programmed cell death (PCD), also known as regulated cell death, refers to a specific form of cell death that can be controlled by various biomacromolecules^9^. For several decades, PCD has been recognized as a pivotal mechanism governing the intricate dynamics of tumor development and progression. The ability of tumor cells to evade or resist PCD exerts a profound impact on their unrestrained proliferation and malignancy. Perturbation of the intricate orchestration of PCD metabolic pathways leads to the accumulation of genetically compromised or aberrant cells, thereby facilitating their relentless persistence and uncontrolled proliferation, ultimately culminating in the formation of tumor masses^10–12^.

PCD encompasses a diverse array of distinct cellular demise mechanisms, including apoptosis, necroptosis, ferroptosis, pyroptosis, netotic cell death, entotic cell death, lysosome-dependent cell death, parthanatos, autophagy, oxeiptosis, cuproptosis, alkaliptosis, and disulfidptosis^13–15^. Apoptosis, a highly regulated form of cell death, is characterized by controlled cellular disassembly and plays a vital role in various physiological processes such as tissue development, immune response regulation, and elimination of damaged cells. Inhibition or resistance of apoptosis often contributes to the development of cancer^16^. Notably, apigenin has been reported to induce the reprogramming of TRAIL/DISC components, rendering lung cancer cells sensitive to TRAIL-mediated apoptosis^17^. Entotic cell death arises from actomyosin-dependent cell-in-cell internalization (entosis) and is executed through lysosome degradation^18,19^. This form of cell death has been observed in various human neoplasms and is presumed to act as an oncosuppression mechanism^20–22^. Autophagy, a cellular process involved in the degradation of cellular components, exerts a dual role in cell survival and cell death^23,24^. Previous evidence suggested that USP15 may negatively regulate lung cancer progression by modulating the TRAF6-BECN1 signaling axis to induce autophagy^25^. Disulfidptosis, a novel form of cell death induced by disulfide stress, is characterized by the breakdown of cytoskeletal proteins and F-actin due to intracellular accumulation of disulfides^14,26^. In addition, the roles of other forms of PCD, such as ferroptosis, cuproptosis, and pyroptosis, in LUAD have been widely discussed^27–29^. However, the comprehensive association between these thirteen forms of PCD and anticancer immunity in LUAD remains unclear.

In the present study, we have identified ten differentially expressed genes associated with PCD in LUAD. Subsequently, we have developed a programmed cell death index (PCDI) to elucidate the relationship between these model genes, PCDI, and the carcinogenesis of LUAD. Furthermore, we have comprehensively characterized the genetic and mutation landscape of these genes in LUAD and formulated a prognostic model for accurately predicting the survival outcomes of LUAD patients. Additionally, we have investigated the intricate interplay between the model genes, PCDI levels, and the immune system. Moreover, we have examined and validated the therapeutic response of the model genes and PCDI to immunotherapy and targeted therapy in the context of LUAD.

## Results

### The workflow of this study

**Fig. 1:**
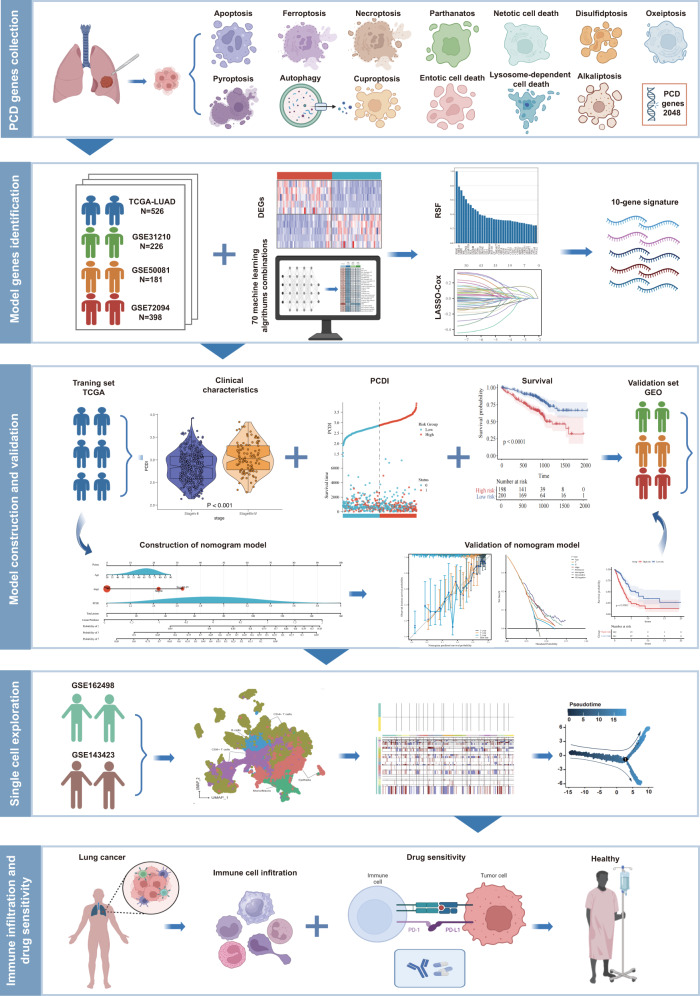
Graphic abstract of this study. Created with BioRender (https://biorender.com/).

We conducted a comprehensive reanalysis of multiple previously published cohorts to train and validate our predictive model. These analyses included three bulk RNA cohorts (TCGA-LUAD, GSE116959, and GSE31210) as well as two single-cell RNA datasets (GSE162498 and GSE143423). In total, we examined thirteen patterns of PCD involving a concatenated set of 2090 genes (Supplementary Table 2). The flowchart depicting the study is presented in Fig. 1.

### Variant landscape of programmed cell death genes in LUAD patients

**Fig. 2:**
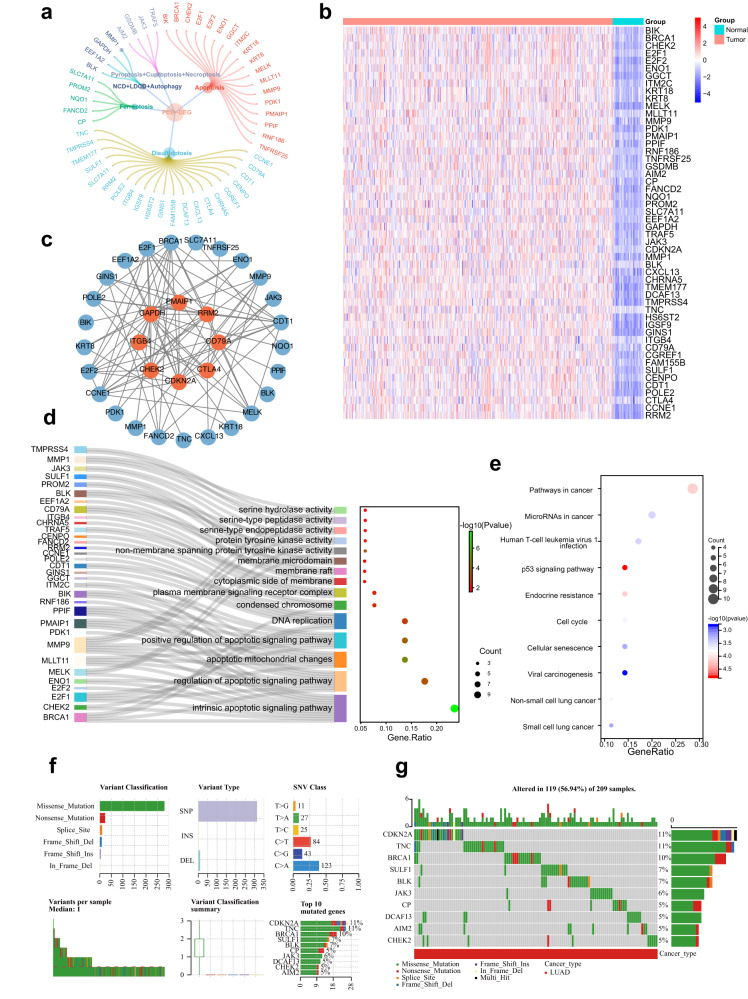
Landscape of PCD-DEGs in LUAD patients. **a** The PCD-DEGs gene list contains 52 genes. **b** Heatmap of the PCD-DEGs between LUAD and normal tissues. **c** PPI network of the interactions of the PCD-DEGs. **d** GO enrichment analyses based on the PCD-DEGs. **e** KEGG enrichment analyses based on the PCD-DEGs. **f**, **g** Diagram of somatic mutations for the PCD-DEGs.

From the TCGA-LUAD and GSE116959 cohorts, we identified 52 DEGs with statistical significance (all adjusted *p* < 0.05 and log2FC > 1), of which 20 were from disulfidocytosis, 18 were from apoptosis, 5 were from ferroptosis, 2 (GSDMB and AIM2) were from pyroptosis, 1 (CDKN2A) was from cuproptosis, 2 (TRAF5 and JAK3) was from necroptosis, 1 (MMP1) was from netotic cell death, 1 (BLK) was from lysosome-dependent cell death, and 2 (EEF1A2 and GAPDH) were from autophagy (Fig. 2a). The complete list of DEGs can be found in Supplementary Table 3. Heatmaps displaying the scaled RNA levels of DEGs are shown in Fig. 2b, while the protein–protein interaction network of the DEGs is depicted in Fig. 2c. Furthermore, Gene Ontology (GO) and Kyoto Encyclopedia of Genes and Genomes (KEGG) enrichment analyses revealed that these DEGs are involved in various carcinogenesis-associated pathways associated carcinogenesis, such as intrinsic apoptotic signaling, p53 signaling, and pathways in cancer (Fig. 2d, e). Additionally, we examined the mutational landscape of PCD-related genes in LUAD patients from the TCGA cohort. The top 10 mutations of PCD-related genes were analyzed and presented, with CDKN2A and TNC exhibiting the highest mutation frequency (11%), while the remaining eight genes demonstrated a relatively lower mutation frequency ranging from 5 to 10% (Fig. 2f).

### Construction of a prognostic gene signature via the machine learning-based integrative procedure for LUAD patients

**Fig. 3:**
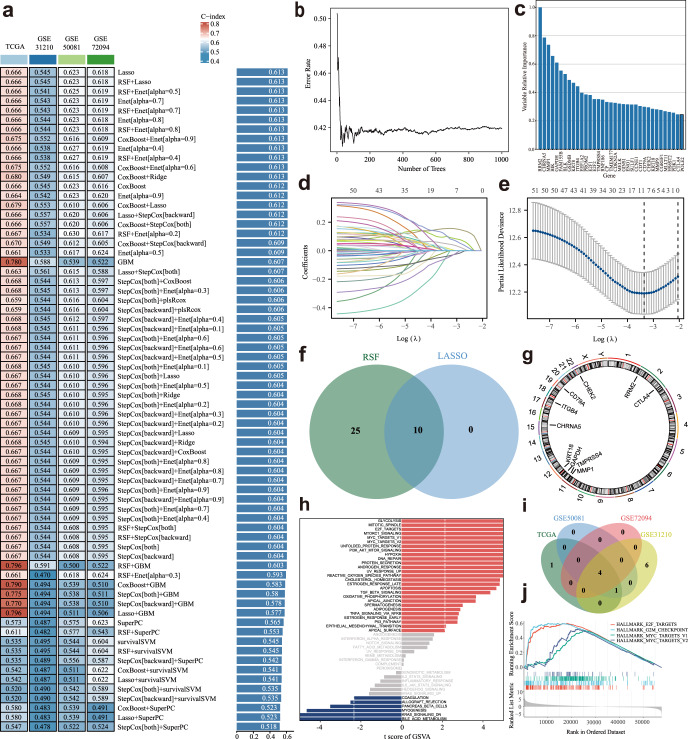
A consensus PCDI was developed and validated via the machine learning-based integrative procedure. **a** A total of 70 kinds of prediction models via a ten-fold cross-validation framework further calculated the C index of each model. **b** The error rate of the RSF result. **c** The variable relative importance of screened genes based on RSF. **d**, **e** Visualization of LASSO regression in the TCGA-LUAD cohort. The optimal λ was obtained when the partial likelihood of deviance reached the minimum value. **f** Venn plot of LASSO results and RSF results. **g** Circos plot depicting the locations and expression levels of 10 model genes. **h** GSVA of the subgroups categorized by PCDI. **i** Venn plot of enrichment biological functions among the TCGA cohort, GSE31210 cohort, GSE50081, and GSE72094 cohort. **j** The 4 common biological pathways.

We employed a machine learning-based integrative procedure to develop a prognostic PCDI using the expression profiles of 52 PCD-related DEGs. In the TCGA-LUAD dataset, we fitted 70 types of predictive models using the LOOCV framework and calculated the C-index for each model (Fig. 3a, Supplementary Table 4). Notably, the top two model combinations with the highest C-index were Lasso and RSF+Lasso. We thereby applied these two algorithms for model gene selection and model construction and the top 35 genes with higher variable importance were identified (Fig. 3b, c). After intersecting these 35 genes with those identified through Lasso regression analysis, resulting in the extraction of 10 genes (Fig. 3d–f). The chromosomal location of each gene is depicted in Fig. 3g.

Subsequently, we constructed the PCDI model using the Lasso regression method and calculated the PCDI for each patient using the following formula: PCDI = (−0.028463286 × CHEK2 exp) + (0.099357443 × KRT18 exp) + (0.156214065 × GAPDH exp) + (0.01656202 × MMP1 exp) + (0.007265954 × CHRNA5 exp) + (−0.003710263 × TMPRSS4 exp) + (0.047539452 × ITGB4 exp) + (−0.037942721 × CD79A exp) + (−0.049874867 × CTLA4 exp) + (0.09887913 × RRM2 exp). Using the median PCDI, we stratified patients with LUAD from the TCGA-LUAD, and three independent GEO cohorts were divided into high-risk and low-risk subgroups. To investigate the underlying biological processes with these subgroups, we performed Gene set variation analysis (GSVA). Figure 3h presents the enriched biological processes specially observed in the TCGA-LUAD dataset, while Fig. 3i, j highlight four common processes identified across all four datasets were identified. Further details of the pathways can be found in Supplementary Table 5.

To assess the significance of the model genes, we compared their expression levels between LUAD tissues and normal samples using the Wilcoxon test (Supplementary Fig. 1). Additionally, We conducted Kaplan‒Meier analysis to investigate their influence on the OS of LUAD (Fig. S1). Remarkably, all model genes, except for CHEK2, TMPRSS4, and CD79A exhibited a significant influence on OS time. The coexpression pattern of the model genes is visually represented in Supplementary Fig. 2.

### Association of PCDI with clinicopathologic features in LUAD patients

**Fig. 4:**
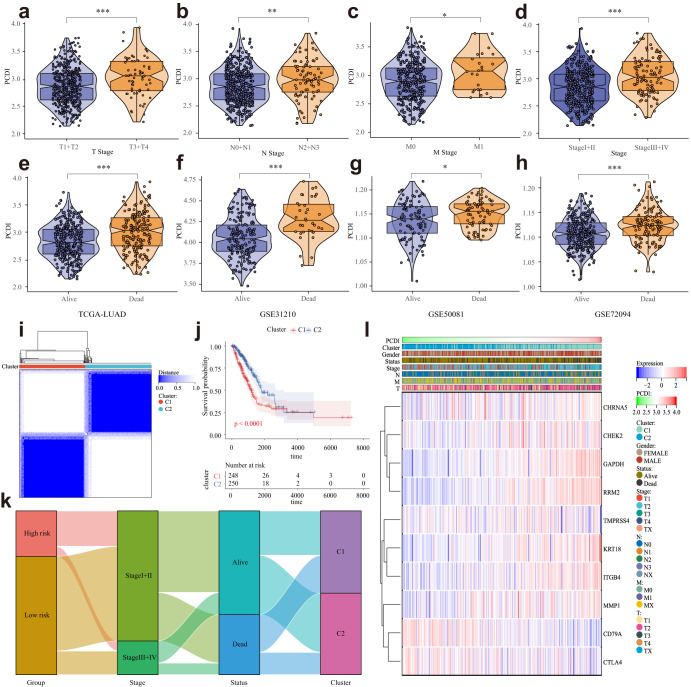
Correlation between the PCDI and Clinical Indicators. **a**–**e** Violin plots of the relationship between PCDI and T stage, N stage, M stage, clinical stage, and survival status in TCGA-LUAD cohort. **f**–**h** Violin plots of the relationship between PCDI and survival status in GSE31210, GSE50081, and GSE72094. **i** TCGA-LUAD cohort patients were grouped into two molecular clusters when *k* = 2 based on the PCD-DEGs profile. **j** Kaplan‒Meier analysis of the prognosis of TCGA-LUAD cohort patients in the two molecular clusters. **k** Alluvial diagram showing the interrelationship between the PCDI group, molecular clusters, clinical status, and survival status in LUAD patients. **l** Heatmap of 10 model genes and clinical factors. **** Means *P* < 0.0001; ** Means *P* < 0.01; * Means *P* < 0.05; ns Means not significant.

We conducted a comparative analysis of variables, including T stage, N stage, M stage, clinical stage, and survival status (alive or dead). Significantly, a significant association was observed between low PCDI and high PCDI groups (Fig. 4a–e, l). Consistent findings were also noted in the GSE31210, GSE50081, and GSE72094 datasets (Fig. 4f–h). Notably, we also conducted a detailed analysis to investigate the influence of diverse histological phenotypes on the levels of PCDI within LUAD. Intriguingly, our results revealed no statistically significant differences in PCDI levels among the different histological types of LUAD, suggesting that PCDI may exhibit minimal variability among the diverse histological subtypes in LUAD (Fig. S4a). Leveraging the model genes, we stratified the LUAD patients in the TCGA cohort into two distinct clusters, with patients in cluster 2 exhibiting a more favorable prognosis. Moreover, the alluvial diagrams and heatmap provided visual evidence that the majority of patients in cluster 1 were characterized by an advanced clinical stage and high PCDI, while the majority of patients in cluster 2 displayed an early clinical stage and low PCDI (Fig. 4k, l).

### Validation of the clinical significance of the prediction model in LUAD datasets

**Fig. 5:**
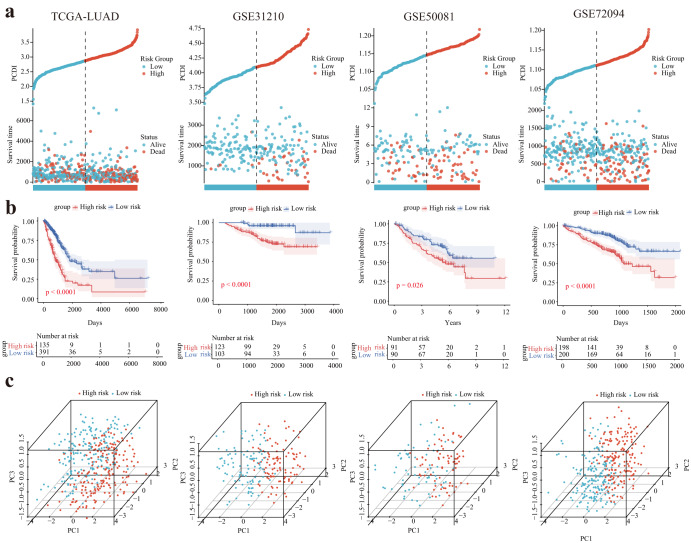
Internal training and external validation of the gene signature prediction model. **a** Distribution of PCDI according to survival status and time in the TCGA, GSE31210, GSE50081, and GSE72094 cohorts. **b** Principal component analysis (PCA) plot based on the PCDI in the TCGA, GSE31210, GSE50081, and GSE72094 cohorts. **c** Overall survival in the low- and high-PCDI group patients in the TCGA, GSE31210, GSE50081, and GSE72094 cohorts.

Utilizing the calculated PCDI value, we stratified LUAD patients in the TCGA-LUAD, GSE31210, GSE50081, and GSE72094 cohorts into PCDI-High and PCDI-Low groups. Our findings revealed a significant association between high PCDI and unfavorable clinical outcomes (Fig. 5a, b). Furthermore, we observed that the PCDI-High and PCDI-Low groups could be effectively distinguished in all four cohorts through PCA (Fig. 5c).

### Development and evaluation of the nomogram survival model

**Fig. 6:**
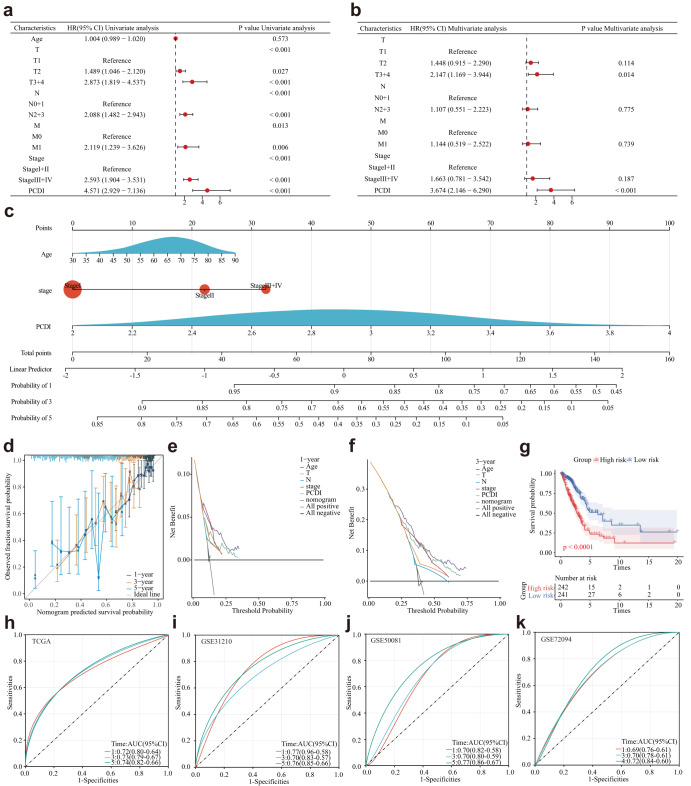
Establishment and assessment of the nomogram survival model. **a** Univariate analysis of the clinicopathologic characteristics and PCDI in the TCGA-LUAD cohort. **b** Multivariate analysis of the clinicopathologic characteristics and PCDI in the TCGA-LUAD cohort. **c** A nomogram was established to predict the prognosis of TCGA-LUAD patients. **d** Calibration plots showing the probability of 1-, 3-, and 5-year overall survival in the TCGA-LUAD cohort. **e–f** Decision curve analysis (DCA) of a nomogram predicting 1- and 3-year overall survival. **g** Kaplan‒Meier analyses for the two LUAD groups based on the nomogram score. **h–k** Receiver operator characteristic (ROC) analysis of the nomogram in the TCGA-LUAD, GSE31210, GSE50081, and GSE72094 cohorts.

To assess the independent prognostic significance of PCDI, we conducted both univariate and multivariate Cox regression analyses. Our findings revealed that PCDI was a significant risk factor in univariate Cox regression analysis (HR = 4.751, 95% CI: 2.929–7.136, *P* < 0.001, Fig. 6a). Furthermore, in the multivariate analysis, PCDI retained its independent prognostic value in LUAD patients even after adjusting for other confounding factors (HR = 3.674, 95% CI: 2.146–6.290, *P* < 0.001, Fig. 6b).

Based on the results from multivariable Cox and stepwise regression analyses, we constructed a prognostic nomogram model in the TCGA cohort to predict the 1-, 3-, and 5-year overall survival (OS) of LUAD patients (Fig. 6c). The calibration curves demonstrated the accurate predictive ability of the nomogram model for the 1-, 3-, and 5-year survival rates (Fig. 6d). Similar findings were also observed in the three validation cohorts (Supplementary Fig. 3). Additionally, DCA confirmed that the nomogram model outperformed other predictors utilized in the study (Fig. 6e, f). Notably, a significant survival difference was observed between the high- and low-risk groups based on the nomogram score (Fig. 6g). To evaluate the performance of the nomogram, we accessed its predictive ability in four independent cohorts. Our results indicated high area under the curve scores for predicting the 1-, 3-, and 5-year survival of LUAD patients (Fig. 6h). Similar results were observed in the validation cohort (Fig. 6i–k).

Subsequently, we conducted a further investigation into the Mixed and NOS subtypes of LUAD, and the findings were consistent with those observed in the overall LUAD patients. Specifically, the low PCDI group exhibited a significantly higher survival rate compared to the high PCDI group within the Mixed and NOS subtypes (Supplementary Fig. 4d, g). To enhance prognostic accuracy, we developed a prognostic nomogram model to predict the 1-, 3-, and 5-year overall survival in Mixed and NOS patients (Supplementary Fig. 4b, c). The calibration and ROC curves demonstrated the model’s proficiency in accurately predicting survival rates at these time intervals (Supplementary Fig. 4e, f, h, i). These findings highlight the potential of the prognostic nomogram model to provide valuable prognostic information for patients with the Mixed and NOS subtypes of LUAD.

### Single-cell analysis suggests the CDIscore correlates with the development of LUAD

**Fig. 7:**
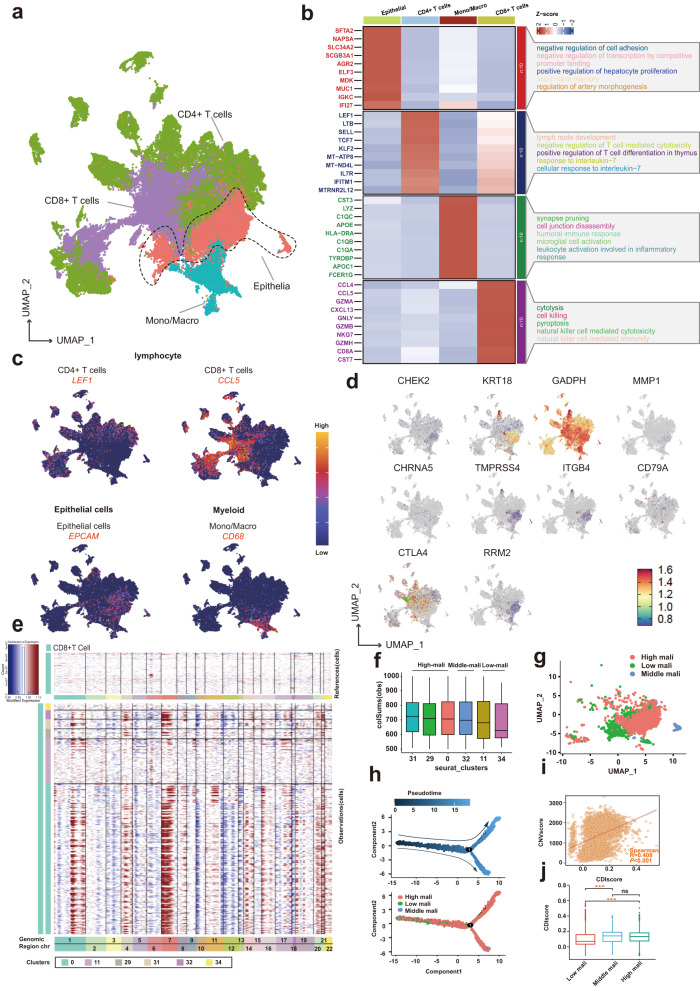
Dissection of tumor microenvironment based on the PCDI signature. **a** UMAP plots for 80059 cells in two LUAD single-cell datasets. **b** Heatmap showing the top differentially expressed genes (Wilcoxon test) in each cell type. **c** Feature plots present the typical marker gene expression for each cell type. **d** The distribution and expressions of ten model genes across different cell types. **e** Heatmap plots showing the CNV signals for each epithelial cluster. **f** Box plots of the score for each epithelial cluster. **g** U-MAP plot showing the three subclusters of epithelial cells. **h** The trajectory of the differentiation process is colored by state and cell type. **i** The correlation between CNVscore and CDIscore. **j** Comparison of the CDIscore between low and middle/high malignant subclusters.

To probe the expression and distribution of PCD-related genes at a single-cell resolution, we scrutinized the scRNA sequencing data of two LUAD datasets. After employing various standard quality control procedures, a total of 80059 cells were included for downstream analysis (Supplementary Fig. 5a, b). The cells were sorted into 38 clusters and eight cell types (Supplementary Fig. 5c, d), with the respective marker genes of each cell type detailed in Fig. 7a–c. The distribution and expressions of ten model genes across different cell types were shown in Fig. 7d. Using the inferCNV algorithm, we detected significant copy number variations in each epithelial cell (Fig. 7e), and the CNV score of each cluster was calculated. As shown in Fig. 7f, g, the epithelial cells were separated into high-malignancy (clusters 31, 29, and 0), middle-malignancy (cluster 32), and low-malignancy (clusters 11 and 34) groups. Pseudotime trajectory analysis using Monocle 2 was then employed to understand the underlying evolution of epithelial cells with diverse CNV scores (Fig. 7h). Finally, we calculated the score of each cell using the “AddModuleScore” function (Supplementary Fig. 5e), and we found that the CDI score was positively correlated with the CNVscore and that the cells with higher CNVscores (intermediate/high malignancy) had higher CDI scores (Fig. 7i, j).

### PCDI correlates with the immune features of LUAD patients

**Fig. 8:**
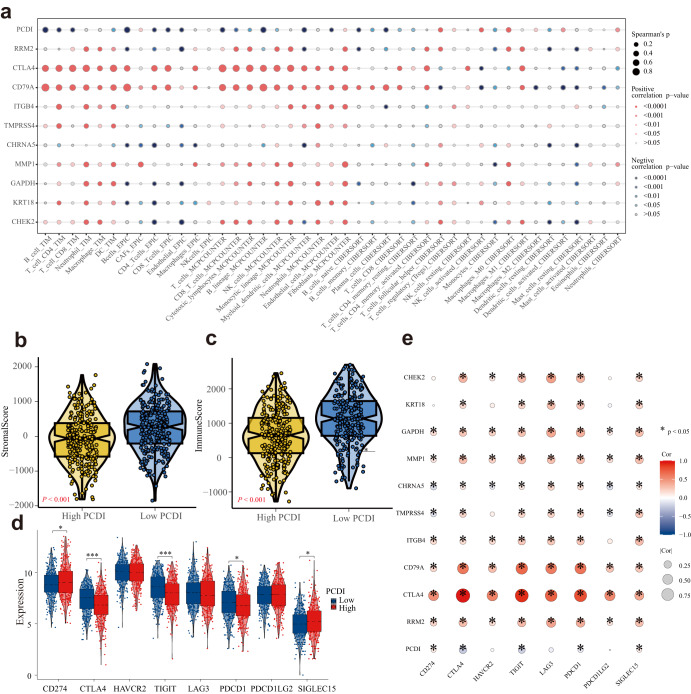
Molecular and immune profiling of PCDI subgroups. **a** Bubble plot of the relationship between immune cells, PCDI, and model genes based on the TIMER, EPIC, MCP-COUNTER, and CIBERSORT algorithms. **b**, **c** Violin plots of the comparison of Stromalscore and Immunescore between high- and low-PCDI groups in TCGA-LUAD. **d** Boxplot of the expression of 8 immune checkpoint genes based on PCDI. **e** Bubble plot of the relationship between immune checkpoint genes, PCDI, and model genes.

We employed a variety of algorithms, including TIMER, EPIC, MCP-COUNTER, ESTIMATE, and CIBERSORT to comprehensively investigate the infiltration of immune cells across different PCDI groups within the TCGA-LUAD cohorts. Notably, we observed a significant negative correlation between PCDI and anticancer immunity-associated cells, such as CD8^+^ T cells, CD4^+^ memory T cells, and myeloid dendritic cells (Fig. 8a). Conversely, PCDI exhibited positive correlations with cell types such as Cancer-Associated Fibroblasts(CAFs), fibroblasts, activated NK cells, M0 macrophages, and neutrophils, as depicted in Fig. 8a. The Stromalscore and Immunescore of the low PCDI group were found to be higher compared to those of the high PCDI group, as shown in Fig. 7b, c. In addition, we explored the relationship between immune checkpoint molecules, PCDI, and the model gene expression levels in the high and low PCDI groups. Our analysis of immune checkpoint molecules revealed downregulation of CTLA-4, TIGIT, and PDCD1 in the high PCDI group, whereas an upregulation was observed for CD274/SIGLEC15, as illustrated in Fig. 8d, e. These findings provide valuable insights into the intricate interplay between PCDI, immune cell infiltration, and immune checkpoint regulation in LUAD.

### Predictive effects of PCDI in immunotherapy

**Fig. 9:**
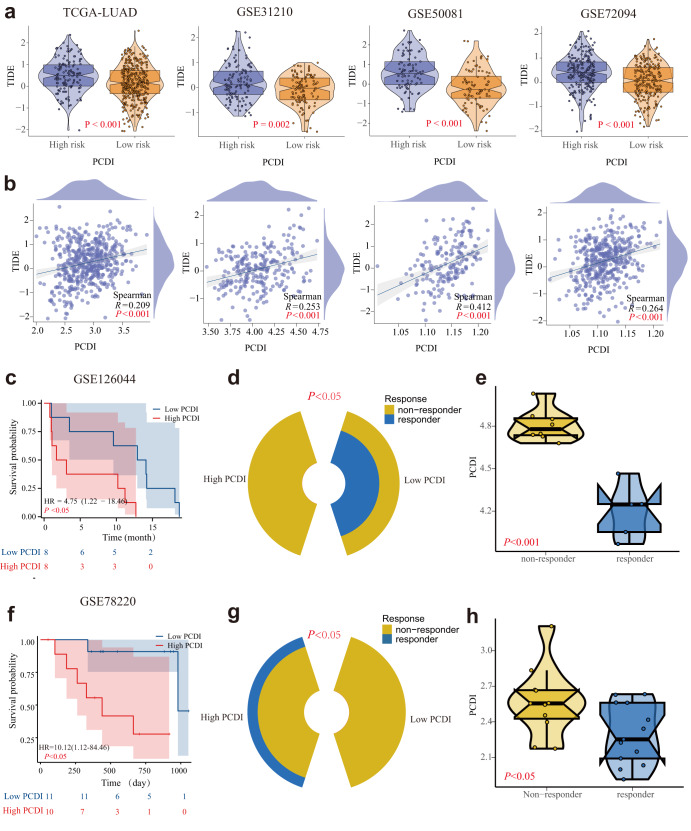
Predictive effect of PCDI in immunotherapy. **a** Violin plots of the comparison of TIDE scores between high- and low-PCDI groups in TCGA-LUAD, GSE31210, GSE50081, and GSE72094 patients. **b** The correlation between the TIDE score and PCDI values in TCGA-LUAD, GSE31210, GSE50081, and GSE72094 patients. **c** Survival curve showing the low-PCDI group had a better prognosis than the high-PCDI group in GSE126044. **d** The proportion of patients with clinical response to anti-PD-L1 treatment in high or low PCDI groups in GSE126044. **e** Violin plots of the PCDI between non-responder and responder GSE126044. **f** The survival curve shows that the low-PCDI group had a better prognosis than the high-PCDI group in GSE78220. **g** The proportion of patients with clinical response to anti-PD-L1 treatment in high or low PCDI groups in GSE78220. **h** Violin plots of the PCDI between non-responder and responder in GSE78220.

Significant positive correlations between TIDE scores and PCDI values across four LUAD cohorts (Fig. 9a, b) were observed, indicating that patients with elevated PCDI may not benefit from immunotherapy. Utilizing the GSE126044 and GSE78220 immunotherapy cohorts, we further evaluated the ability of PCDI to predict the response of LUAD patients to anti-PD-L1 treatment. Patients exhibiting high PCDI displayed worse survival compared to those with low PCDI (Fig. 9c, f). The percentage of patients responding to anti-PD-L1 in the high PCDI group was markedly lower than that in the low PCDI group (Fig. 9d, g). Furthermore, non-responders exhibited higher PCDI than responders (Fig. 9e, h).

### PCDI correlates with the targeted therapy response of LUAD patients

**Fig. 10:**
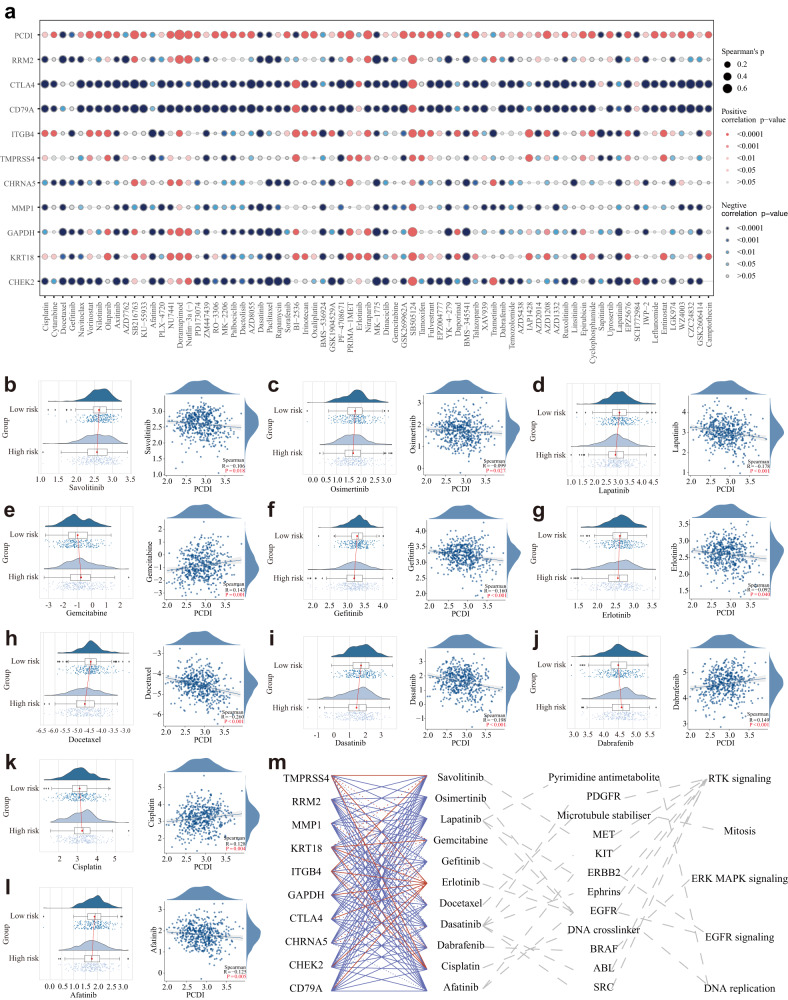
Efficacy of programmed cell death signature in predicting drug sensitivity. **a** Bubble plot of the relationship between drugs, PCDI, and model genes. **b**–**l** Boxplots of the comparison of IC50 of drugs used for treating NSCLC between high- and low-PCDI groups and correlation between the IC50 and PCDI values in the TCGA-LUAD cohort. **m** The correlation between model genes, drugs, classical therapeutic targets, and pathways in TCGA-LUAD.

To explore the potential relationship between our constructed model and drug sensitivity, we analyzed the half-maximal inhibitory concentration (IC50) values for several drugs using the GDSC database in LUAD samples. The correlation and significance between the IC50 of various drugs and the prognostic PCDI are presented in Fig. 10a and Supplementary Table 6. Particularly, we observed higher IC50 values of gemcitabine and cisplatin in the high PCDI group compared to the low PCDI group (Fig. 10e, k). Interestingly, we found that the IC50 values of savolitinib, osimertinib, lapatinib, gefitinib, erlotinib, dasatinib, and afatinib were lower in the high PCDI score group (Fig. 10b–l). Furthermore, we investigated the correlations between the model genes, FDA-approved drugs for lung cancer, classical therapeutic targets, and signaling pathways, as shown in Fig. 10m. These findings suggest a potential association between our model genes and drug sensitivity, providing valuable insights for personalized treatment strategies in LUAD.

### In-vitro experiments validation of signature genes in clinical samples

**Fig. 11:**
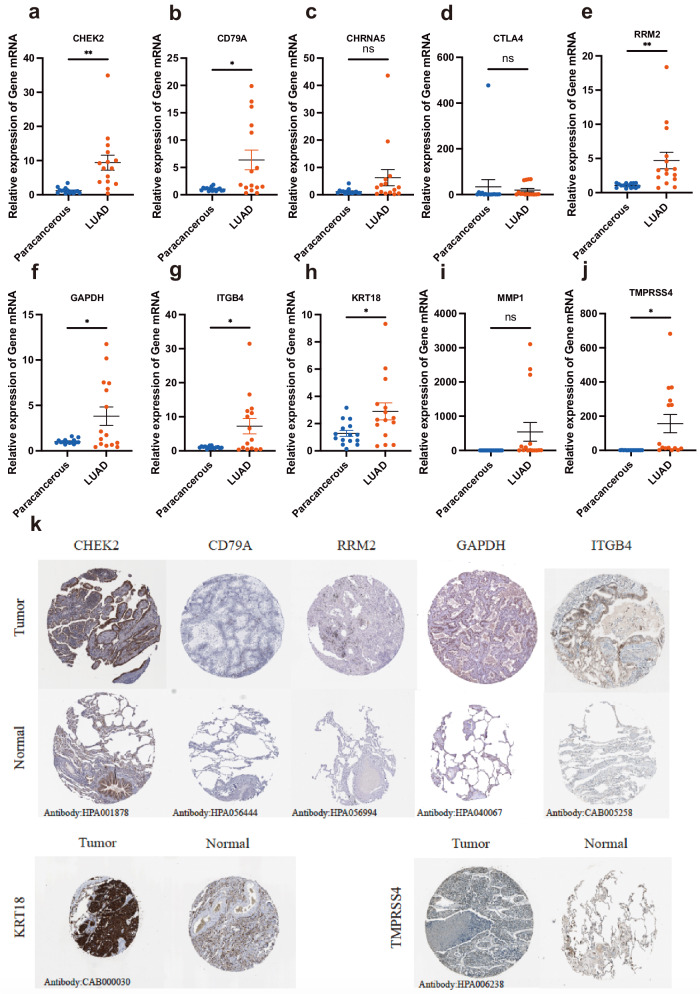
In-vitro experiments validation of signature genes in clinical samples. **a**–**j** RT-qPCR verifying the gene transcription in TCGA-LUAD and precancerous tissues. **k** The protein expression of signature genes between TCGA-LUAD and adjacent normal tissues in the HPA database.

We utilized RT-qPCR analysis to validate the differential mRNA expression of 10 model genes between the adjacent and LUAD tissues from clinical samples. The results showed that CHEK2, CD79A, RRM2, GAPDH, ITGB4, KRT18, and TMPRSS4 were overexpressed in LUAD than in paracancerous samples, whereas the expression of CHRNA5, CTLA4, and MMP1 showed no statistical significance (Fig. 11a–j). Additionally, we verified the expression of the model genes at the protein level between LUAD and adjacent normal tissues using the Human Protein Atlas (HPA) database (available from www.proteinatlas.org) under a Creative Commons Attribution (CC BY) license. Immunohistochemistry analysis demonstrated lower staining in normal tissue, whereas higher staining of CHEK2, RRM2, GAPDH, ITGB4, KRT18, and TMPRSS4 was observed in LUAD samples at the protein level (Fig. 11).

**Fig. 12:**
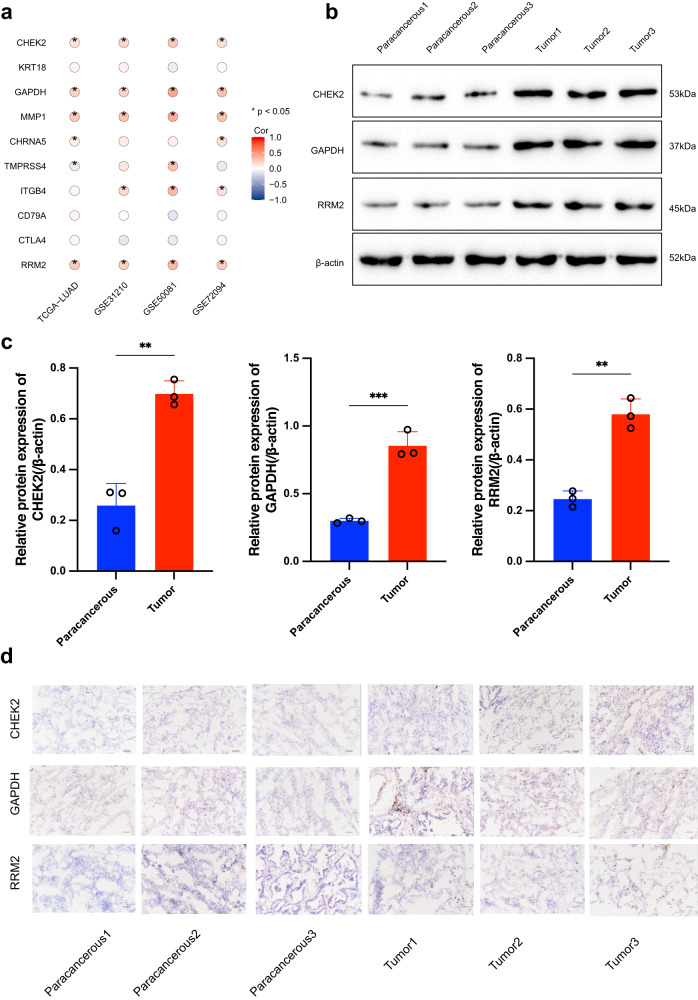
Validation of signature genes in clinical samples through In vitro *e*xperiments. **a** Bubble plot of the relationship between model genes and TIDE scores. **b**, **c** WB analyses of CHEK2, GAPDH, and RRM2 in clinical samples. **d** immunohistochemical analyses of CHEK2, GAPDH, and RRM2 in Clinical Samples. Scale bars: 50 μm.

To clarify the link between gene expression and TIDE, we delved into a thorough analysis of both training and validation datasets. Figure 12a highlights four key genes—CHEK2, GAPDH, MMP1, and RRM2—common to all four cohorts. Considering gefitinib’s role as a prevalent NSCLC therapy, we assessed its sensitivity correlation within the TCGA-LUAD dataset, pinpointing five primary genes: CHEK2, ITGB4, RRM2, GAPDH, and KRT18, as listed in Supplementary Table 6. Cross-referencing these with the quartet from Fig. 12a isolated three candidates—CHEK2, GAPDH, and RRM2—for closer scrutiny. Western blotting confirmed their heightened expression in tumors versus normal tissue (Fig. 12b), quantified in Fig. 12c. Immunohistochemistry further validated the pronounced presence of these genes in tumor samples, depicted in Fig. 12d.

## Discussion

In our study, we identified 52 PCD-related DEGs and developed a consensus prognostic PCDI employing machine learning algorithms. Based on the PCDI and clinical features, we generated several prognostic nomograms across the training and validation LUAD cohorts, which exhibited robust and consistent performance. Importantly, our findings revealed a significant correlation between PCDI and the tumor microenvironment (TME) as well as drug sensitivity in LUAD. These results emphasize the potential clinical applications of the PCDI in guiding personalized treatment decisions.

It has long been established that various PCD patterns are closely associated with the development and metastasis of human tumors^30^. Herein, we developed a signature comprising 10 PCD-related genes (CHEK2, KRT18, GAPDH, MMP1, CHRNA5, TMPRSS4, ITGB4, CD79A, CTLA4, and RRM2) utilizing multiple LUAD datasets and conducted a comprehensive bioinformatic analysis to investigate the genetic landscape and clinical relevance of these model genes in LUAD.

To corroborate our bioinformatic observations, RT-qPCR analysis was employed to juxtapose the expression of model genes across LUAD and precancerous tissues. The results predominantly corroborated our expectations, except for CTLA4, MMP1, and CHRNA5 at the mRNA level, warranting further exploration at the protein level. CHK2 is a critical component involved in various molecular processes, including DNA structure modification, cell cycle progression, and DNA damage response. Disruption of these checkpoints can lead to genomic instability, cell death, and tumor formation^31,32^. KRT18, a type of cytokeratin, is an intermediate filament protein that plays a role in maintaining tissue integrity. Cytokeratins have been identified as diagnostic and prognostic markers for tumor occurrence, progression, and drug response^33^. Luigi et al. reported that KRT18 exhibits potential as a prognostic marker in NSCLC patients^34^. Although GAPDH is commonly considered a housekeeping gene, it is a widely expressed enzyme with unconventional functions, including involvement in glycolysis^35^. GAPDH acts as an irreversible metabolic switch in glycolysis by catalyzing the conversion of glyceraldehyde-3-phosphate to 1,3-diphosphoglycerate, thereby producing NADH^36^. In NSCLC patient cohorts, GAPDH transcription is upregulated and associated with the glycolysis and gluconeogenesis pathways^37^. RNA interference-mediated knockdown of GAPDH induces cellular senescence in A549 cells and enhances the therapeutic efficacy of metabolic drugs^38^. MMP1, a matrix metalloproteinase, plays a critical role in extracellular matrix remodeling and is involved in tumor invasion, metastasis, and angiogenesis^39^. Overexpression of MMP1 has been observed in various cancers, including lung cancer, and is associated with tumor progression and unfavorable clinical outcomes^40^. CHRNA5, a subunit of the nicotinic acetylcholine receptor, has been implicated in cell proliferation, apoptosis, and carcinogenesis^41^. Genetic variations in the CHRNA5-CHRNA3-CHRNB4 gene cluster have been shown to increase susceptibility to lung cancer^42,43^. TMPRSS4 induces epithelial-to-mesenchymal transition and promotes metastasis in colon cancer cells^44^. Importantly, high expression of TMPRSS4 is associated with poor prognosis in patients with squamous cell carcinomas^45^. ITGB4, a cell surface receptor, is involved in cell adhesion, migration, and invasion^46^. ITGB4 disrupts cell adhesion and basement membrane integrity, thereby promoting cancer progression and metastasis^47^. CD79A, a B-cell receptor-associated protein, plays a critical role in B-cell development and function^48^. CD79A is an important target in classical Hodgkin’s lymphoma^49^. CTLA4, a known immune checkpoint molecule, regulates T-cell activation and the immune response. It has emerged as a therapeutic target in cancer immunotherapy, particularly in approaches to block the immune checkpoint^50^. CTLA-4 enhances the antitumor effect of effector T cells, maintains self-tolerance, and suppresses the function of Tregs in liver cancer immunity^51^. RRM2, a subunit of ribonucleotide reductase, plays a critical role in DNA synthesis and repair^52^. A previous study found that high expression of RRM2 is an independent predictive factor for poor prognosis in patients with LUAD^53^. In the current study, we observed a significant association between the expression of these model genes and the clinical outcomes of LUAD patients, suggesting their potential as prognostic biomarkers in LUAD.

To evaluate the clinical relevance of PCDI in LUAD, we developed a nomogram model that combines PCDI with relevant clinical parameters. The effectiveness of this model was validated, demonstrating its clinical usefulness. We also analyzed PCDI across different histological subtypes and found no statistically significant differences among them. Importantly, we observed that patients with low PCDI had a significantly higher survival rate compared to those with high PCDI. The prognostic nomogram model showed strong predictive ability for 1-, 3-, and 5-year overall survival in patients with Mixed and NOS subtypes. However, due to the complex nature of LUAD, which exhibits diverse histological phenotypes, further comprehensive investigations are necessary to understand the role of the PCDI model in different LUAD types. These investigations should include mechanistic studies, animal studies, and additional clinical analyses.

Tumor cells possess the ability to evade immune surveillance and resist the effects of therapeutic drugs, thereby promoting their survival and progression^54^. Our study has revealed significant differences in the immune microenvironments of tumors based on their levels of PCDI. Notably, tumors with high PCDI exhibited reduced infiltration of anti-tumor immune cells, including B cells, CD4^+^ T cells, and CD8^+^ T cells, compared to tumors with low PCDI. Conversely, immunosuppressive cell types such as cancer-associated fibroblasts (CAFs), fibroblasts, M0 macrophages, and neutrophils were upregulated in tumors with high PCDI. This inverse correlation between PCDI and effector immune cells, along with the enrichment of suppressive leukocytes, suggests that tumors with high PCDI exhibit a more immunosuppressed phenotype^55^. Despite the seemingly favorable prognosis indicated by low PCDI, patients in this subgroup demonstrated increased stromal and immune activities, suggesting hidden risks of disease progression potentially attributed to the aggressive TME^56,57^. While this molecular profile may seem counterintuitive, it necessitates careful monitoring due to the tumor-promoting effects of the TME^58^. Moreover, the abundance of immune infiltrates could be strategically leveraged for immunotherapy, offering the potential to mitigate the detrimental effects of the pro-tumoral environment^59^. A comprehensive analysis of immune checkpoint expression has provided further insights into the attenuated immune response within tumors with high PCDI. We observed downregulation of inhibitory receptors, including CTLA-4, TIGIT, and PD-1, likely as a result of T cell exhaustion due to chronic immune suppression. Concurrently, we observed upregulation of their ligand PD-L1, indicating an adaptive immune resistance mechanism employed by tumor cells. Collectively, these findings suggest an escalation of T cell dysfunction^60^.

From a clinical perspective, the elevated TIDE scores observed in the high PCDI group support the notion of immune evasion being associated with high PCDI^61^. In the cohort undergoing immunotherapy, responders were characterized by lower PCDI, while non-responders exhibited elevated PCDI, potentially attributable to the more potent immune response associated with low PCDI, thus rendering the tumor more susceptible to immunotherapy. These findings underscore the potential utility of PCDI as a predictive biomarker for immunotherapy in LUAD, with high PCDI scores being associated with a diminished therapeutic response.

Our study provided insights into the relationship between the PCD signature and drug sensitivity in LUAD patients. Notably, patients with high PCDI exhibited resistance to standard chemotherapies while demonstrating potential sensitivity to other FDA-approved drugs for NSCLC. These results suggest that PCDI may serve as a predictive marker for personalized treatment selection, facilitating the development of more effective therapeutic strategies for LUAD patients.

While our study provides valuable insights into the clinical implications of the PCDI signature, it is important to acknowledge several limitations. Firstly, the analyses heavily relied on retrospective data, highlighting the need for future studies to validate the clinical relevance of our findings. Given the complex nature of LUAD and its diverse histological phenotypes, conducting more comprehensive mechanistic and clinical investigations is crucial to explore the role of PCD genes in different LUAD subtypes. Additionally, although the differential expression of PCDI genes between LUAD and precancerous samples was confirmed by RT-qPCR, the protein levels of these genes were not further validated. Lastly, the decision-making role of the PCDI model in our study lacks verification from phase 3 randomized controlled trials. Therefore, high-quality, adequately followed-up, multicenter randomized controlled trials with large sample sizes are required to confirm our results.

In conclusion, our findings suggest that PCDI has the potential to be a valuable prognostic predictor for LUAD patients. However, further research addressing the aforementioned limitations is necessary to strengthen the validity and applicability of our findings.

## Methods

### Data acquisition

RNA-seq data and corresponding clinicopathological information for LUAD samples were obtained from The Cancer Genome Atlas (TCGA)^62^. In addition, clinicopathological information and genome-wide expression data for four other LUAD cohorts (GSE116959, GSE31210, GSE50081, and GSE72094) along with the immunotherapy cohort (GSE126044 and GSE78220) were obtained from the Gene Expression Omnibus (GEO) database^63–68^. To compile the list of PCD genes, we gathered genes associated with 13 different PCD patterns from reputable scientific sources, including GSEA gene sets, KEGG, review articles, and manual compilation^69^. After eliminating duplicate genes, a total of 2090 PCD-associated genes were included for subsequent analysis.

### Identification of the expression and variation levels of PCD-related genes

The raw transcriptome count data from TCGA-LUAD and GSE116959 were subjected to preprocessing for subsequent analysis. Differential expression analysis was performed using the “edgeR” package to identify genes that play a crucial role in PCD. The criteria for identifying differentially expressed genes (DEGs) were set as an adjusted *P* < 0.05 and log2-fold change > 1^70^. To explore the somatic mutation landscape within the LUAD patients cohort, we utilized the “map tools”^54^. The diverse characteristics of PCD-related genes were visually represented in a circus plot using the “circlize” R package^71^.

### Signature generated from machine learning-based integrative approaches

To establish a consensus on PCD-related genes with high accuracy and stability, we employed a comprehensive by integrating 10 machine-learning algorithms and 70 algorithm combinations. The integrated algorithms encompassed a range of techniques, including random survival forest (RSF), elastic network (Enet), Lasso, Ridge, stepwise Cox, CoxBoost, partial least squares regression forex (plsRcox), supervised principal components (SuperPC), generalized boosted regression modeling (GBM) and survival support vector machine (survival-SVM). The procedure for generating the signatures involved the following steps: (a) The previously identified DEGs were subjected 70 algorithm combinations to construct predictive models using leave-one-out cross-validation (LOOCV) in the TCGA-LUAD cohort, (b) All models were further cross-validated using three independent datasets (GSE31210, GSE50081, and GSE72094). (c) For each model, the Harrell’s concordance index (C-index) was calculated across all validation datasets, and the model with the highest average C-index was deemed optimal.

### Functional enrichment analysis

The “clusterProfiler” R package was utilized to identify potential Gene Oncology (GO) pathways based on the above-identified DEGs^72^. To compare the distinct biological functions between the high-risk group (high PCDI) and the low-risk group (low PCDI), we employed GSVA analysis using the “hall. v2022.1.Hs.symbols.gm” database^73^.

### Unsupervised clustering of PCD-related model genes

For unsupervised clustering of the PCD-related model genes, we utilized the “ConsensusClusterPlus” R package. The clustering was performed using agglomerative kindest clustering with a Spearman correlation distance metric and 80% of the samples were resampled for 10 repetitions. To determine the optimal number of clusters, we employed an empirical cumulative distribution function plot^74^. The overall survival (OS) of LUAD patients across different clusters was compared using Kaplan‒Meier analysis.

### Nomogram building and assessment based on the PCDI

To validate the value of the PCDI as an independent prognostic indicator for LUAD patients, both univariate and multivariate Cox regression analyses were conducted. These analyses assessed the significance of the PCDI in combination with relevant clinical parameters. Subsequently, prognostic nomograms were developed based on the TCGA-LUAD cohort and three GEO cohorts using the R packages “rms” and “replot”. The performance of these nomograms was evaluated through calibration curves, decision curve analysis (DCA), and receiver operating characteristic (ROC) curves^75^.

### Single-cell sequencing analysis of PCD-related genes

The analysis of single-cell RNA sequencing data from the GEO database (GSE162498 and GSE143423) for LUAD was undertaken with the “Seurat” and “SingleR” packages^76^, following a series of standard quality procedures that included the “PercentageFeatureSet”, “SCTransform”, “RunPCA”, “FindNeighbors”, “FindClusters”, “RunUMAP”, and “FindAllMarkers” functions. Cell types were assigned using the “SingleR” function and known markers from the literature^76^. Additionally, the “ClusterGVis” and “org.Hs.eg.db” R packages were used to identify the biological function of the marker genes in each cell type. The CNV scores of epithelial cells were calculated using CD8 + T cells as a reference via the “inferCNV” package^77^. To explore the developmental trajectory of epithelial cells with diverse CNVscores, the Monocle2 algorithm was used^78,79^. Moreover, the “AddModuleScore” function was utilized to calculate the signature-specific score (CDIscore) based on the PCD-related genes’ average expression.

### Tumor microenvironment analysis and drug sensitivity prediction

The expression data of model genes and various immune cell infiltration levels were obtained and calculated using multiple algorithms, including TIMER, EPIC, MCP-COUNTER, ESTIMATE, and CIBERSORT^80,81^. The association between PCDI and immunotherapy as well as targeted therapy response was analyzed using the Tumor Immune Dysfunction and Exclusion (TIDE) algorithm^82^ and the “oncoPredict” tool^83^, respectively.

### RT-qPCR

Total RNA was extracted using the TRIzol lysis method. The RNA was then reverse-transcribed into complementary DNA (cDNA) using the Hifair® III One-Step RT-qPCR SYBR Green Kit (Yeasen, China). RT-qPCR was conducted using the Hieff® qPCR SYBR Green Master Mix (Yeasen, China), according to the manufacturer’s instructions. The 2-∆∆CT method was used to calculate the relative gene expression levels. Primers were synthesized and designed by GenePharma (Shanghai, China), and their detailed sequences are listed in Supplementary Table 1.

### Western blot analysis

Total protein was isolated using radioimmunoprecipitation and lysis buffer. The extracted proteins were treated with 10% SDS-PAGE and transferred to polyvinylidene fluoride membranes. Membranes were blocked with 5% milk and incubated with the primary antibodies overnight at 4 °C, followed by incubation with the appropriate horseradish peroxidase-conjugated secondary antibodies. Signals were detected using an Enhanced Chemiluminescence Detection Kit (Cell Signaling Technology, Danvers, MA, USA). Antibodies against GPX4 (1: 5000, ab125066, Abcam), SLC7A11 (1: 1000, 26864-1- AP, Abcam), ACSL4(1: 50000, ab155282, Abcam), and GAPDH (1: 15000, 60004-I-Ig, Proteintech) were used as primary antibodies. Uncropped scans of the most important blots were seen in Supplementary Fig. 6.

### Immunohistochemical staining

Formalin-fixed, paraffin-embedded lung sections of 5 µm thickness were dewaxed in xylene and rehydrated through graded ethanol. Endogenous peroxidase activity was blocked with 3% H2O2 for 10 min at room temperature. Antigen retrieval was performed using citrate buffer (10 mM, pH 6.0) and microwave heating. Sections were blocked with a 1:10 dilution of goat serum for 30 min at room temperature before overnight incubation at 4 °C with primary antibodies against CHEK2 (rabbit, 1:100, Absin, Abs106836), RRM2 (rabbit, 1:100, Proteintech, 11661-1-AP), and GAPDH (mouse, 1:100, Proteintech, 60004-1-lg). After washing in PBS, sections were incubated with HRP-conjugated secondary antibodies for 30 min at room temperature, followed by a 20-min incubation with HRP-conjugated broad-spectrum secondary antibodies. Hematoxylin counterstaining was performed to visualize nuclei.

### Ethics approval

Since the sequenced data generated from TCGA and GEO were publicly available, additional ethics committee approval was not necessary.

### Statistical analysis

Statistical analyses were performed using R software (version 4.1.0). Continuous variables were reported as the standard error of the mean and compared using either the Student’s *t* test or Wilcoxon rank sum test. Categorical data were assessed using the chi-square test. Statistical significance was defined as *P* < 0.05.

### Reporting summary

Further information on research design is available in the Nature Research Reporting Summary linked to this article.

### Supplementary information

SUPPLEMENTAL MATERIAL

REPORTING SUMMARY

## Data Availability

The datasets used in this paper are available online, as described in the Methods section.

## Code availability

No new algorithms were developed for this article. All code generated for analysis is available from the authors upon request.
