# Supplementary material for: Machine learning reveals diverse cell death patterns in lung adenocarcinoma prognosis and therapy
Source: NPJ Precis Oncol. 2024 Feb 26;8:49. doi: 10.1038/s41698-024-00538-5 (PMC10897292; doi:10.1038/s41698-024-00538-5)
Supplement: Supplementary file 1 — SUPPLEMENTAL MATERIAL [file 41698_2024_538_MOESM1_ESM.pdf]

Supplementary Figures

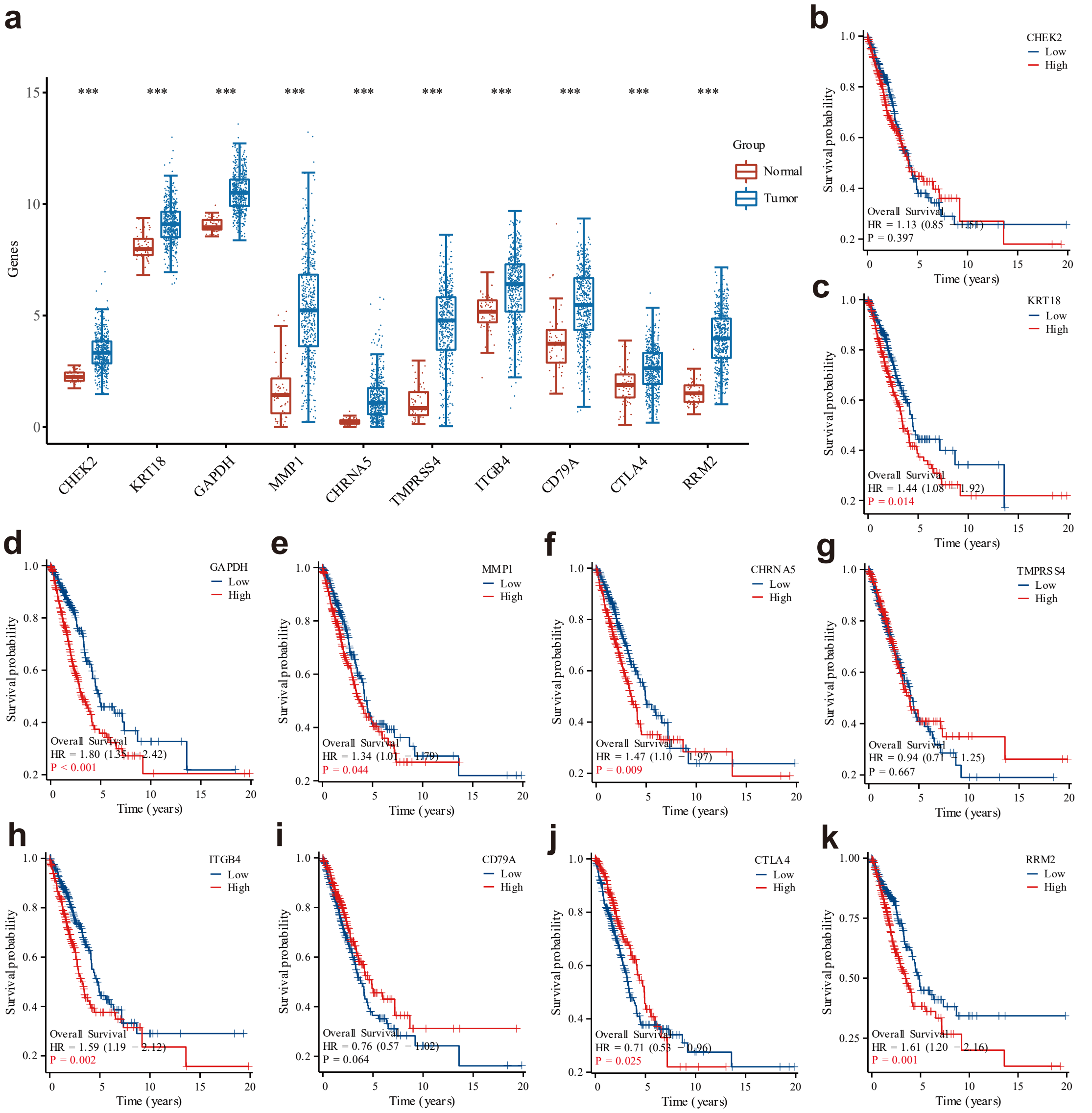

Supplementary Figure 1: Differential Expression and Survival Analysis of PCDS in LUAD. (a) Wilcoxon test of PCDS expression between LUAD tissues (blue) and normal samples (red) in TCGA-LUAD cohort. ****P* < 0.001. (b-k) Kaplan-Meier analysis of PCDS in TCGA-LUAD cohort (blue: low-expression group; red: high-expression group).

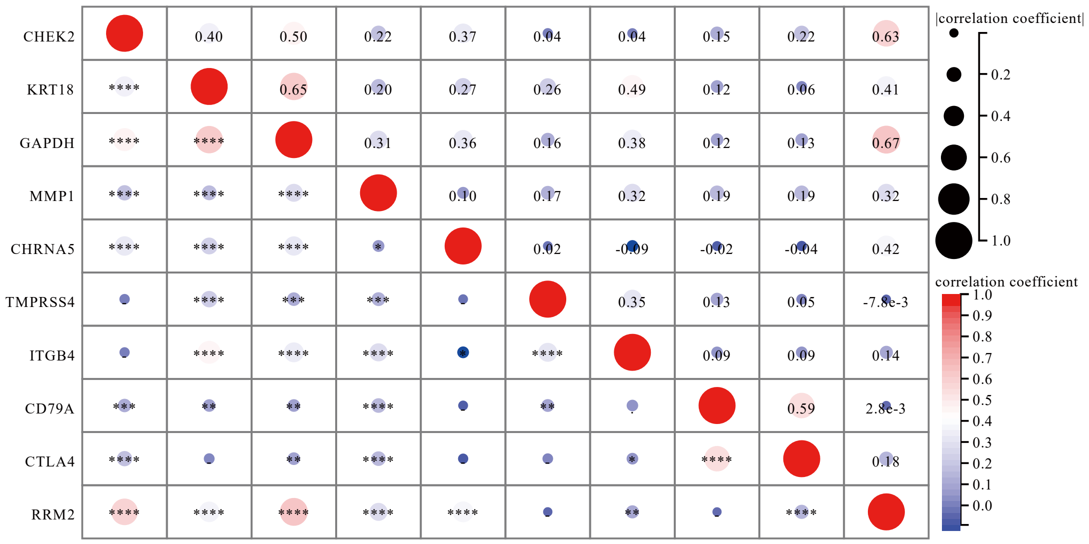

Supplementary Figure 2. The Correlation of Model Genes. Pearson's correlation coefficients (r)for all model genes are shown in the plot. **P*<0.05, ***P*<0.01, *****P* < 0.0001.

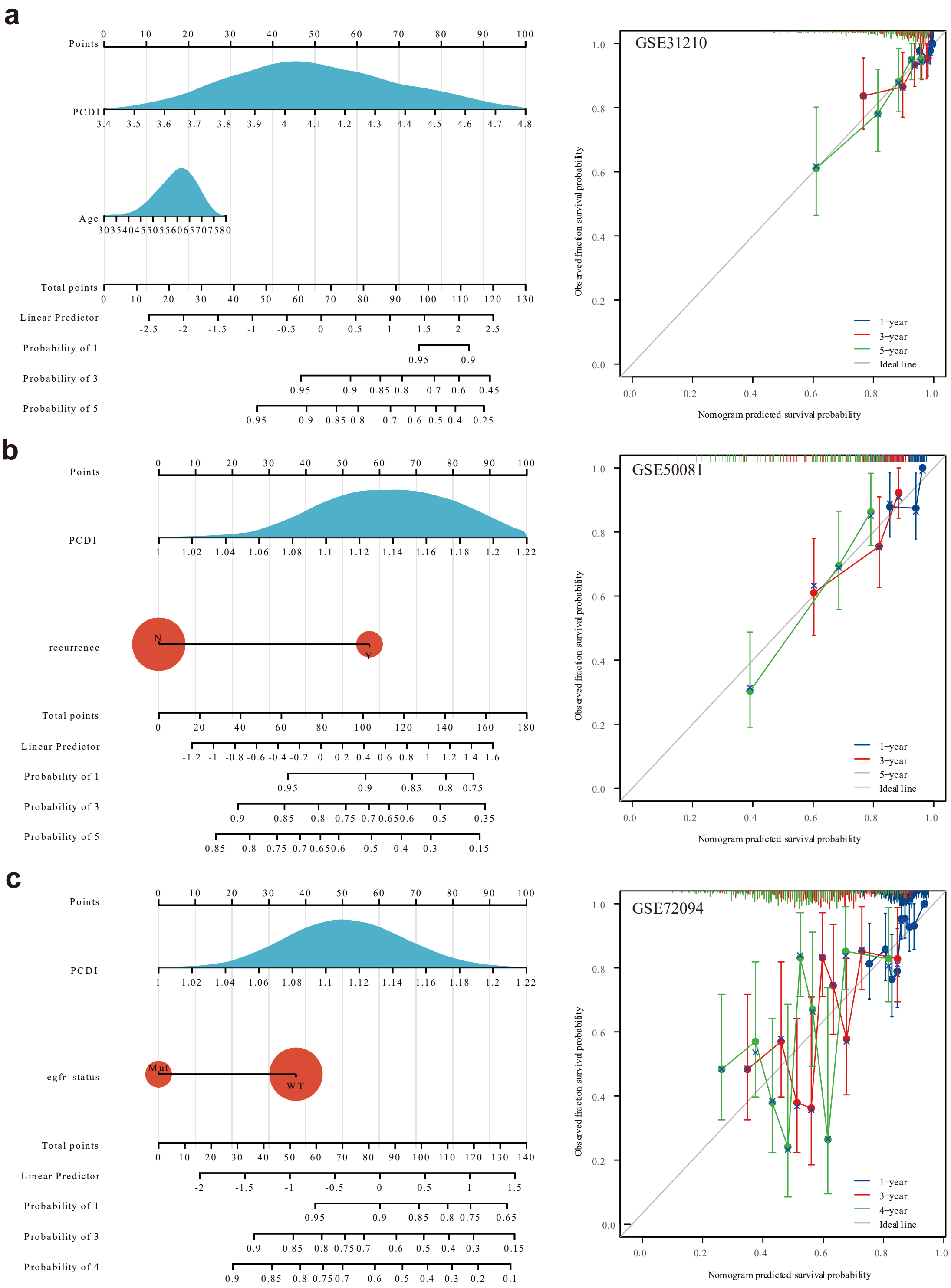

Supplementary Figure 3: Clinical Significance of the PCDI in the Validation Cohort. Construction and validation of the prognostic nomogram model in (a) GSE31210, (b) GSE50081, and (c) GSE72094 cohorts.

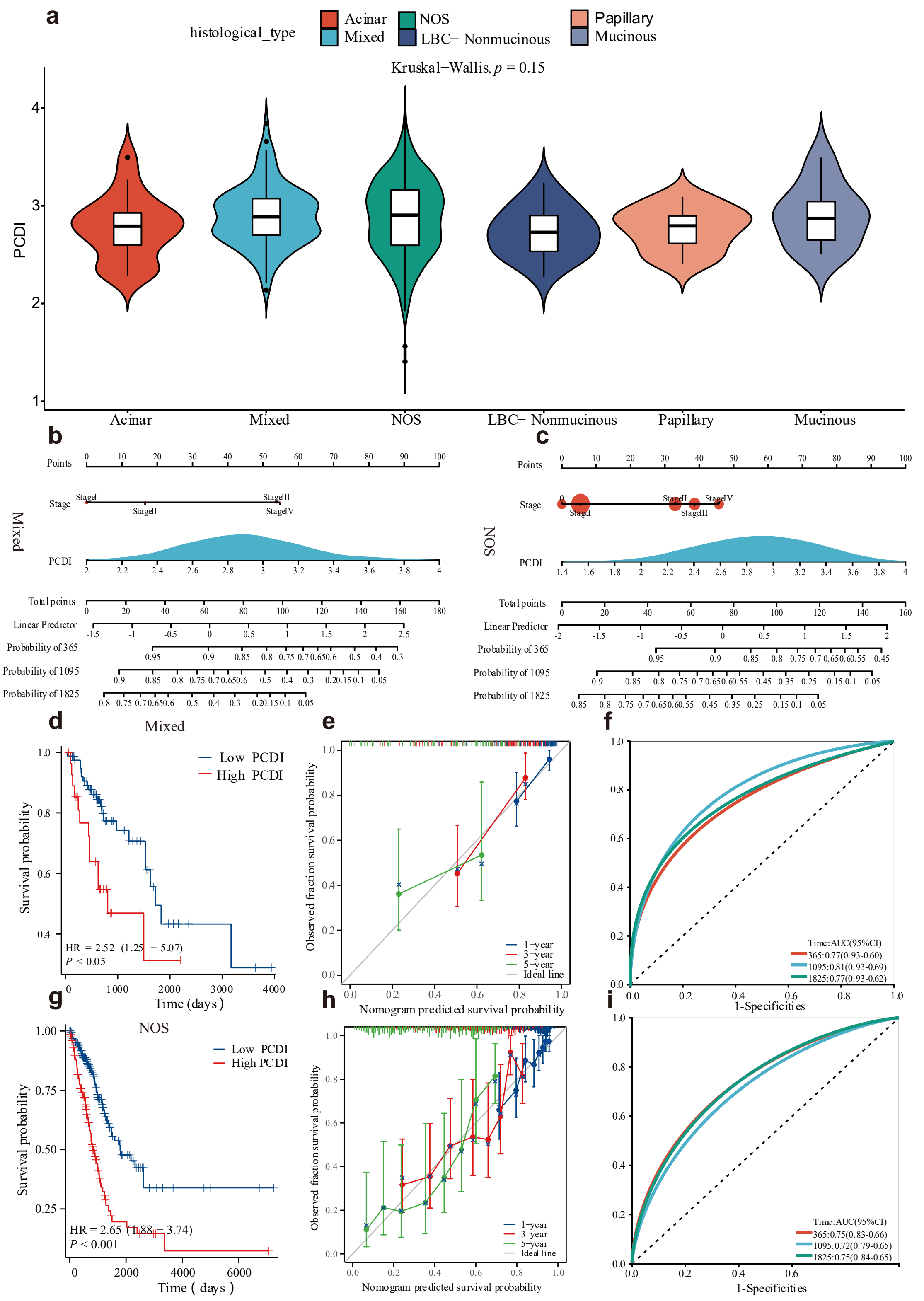

Supplementary Figure 4: Analysis of LUAD subtype. (a) violin plot of PCDI among 6 main LUAD subtypes. (b-c) Construction of nomogram model in Mixed and NOS subtype. (d) Kaplan–Meier analysis of the prognosis of LUAD Mixed patients. (e)

Calibration plots of the probability of 1-, 3-, and 5-year overall survival in the Mixed subtype. (f) Receiver operator characteristic (ROC) analysis of the nomogram in Mixed subtype. (g)Kaplan–Meier analysis of the prognosis of LUAD NOS patients. (h) Calibration plots of the probability of 1-, 3-, and 5-year overall survival in the NOS subtype. (i) Reciver operator characteristic (ROC) analysis of the nomogram in the NOS subtype.

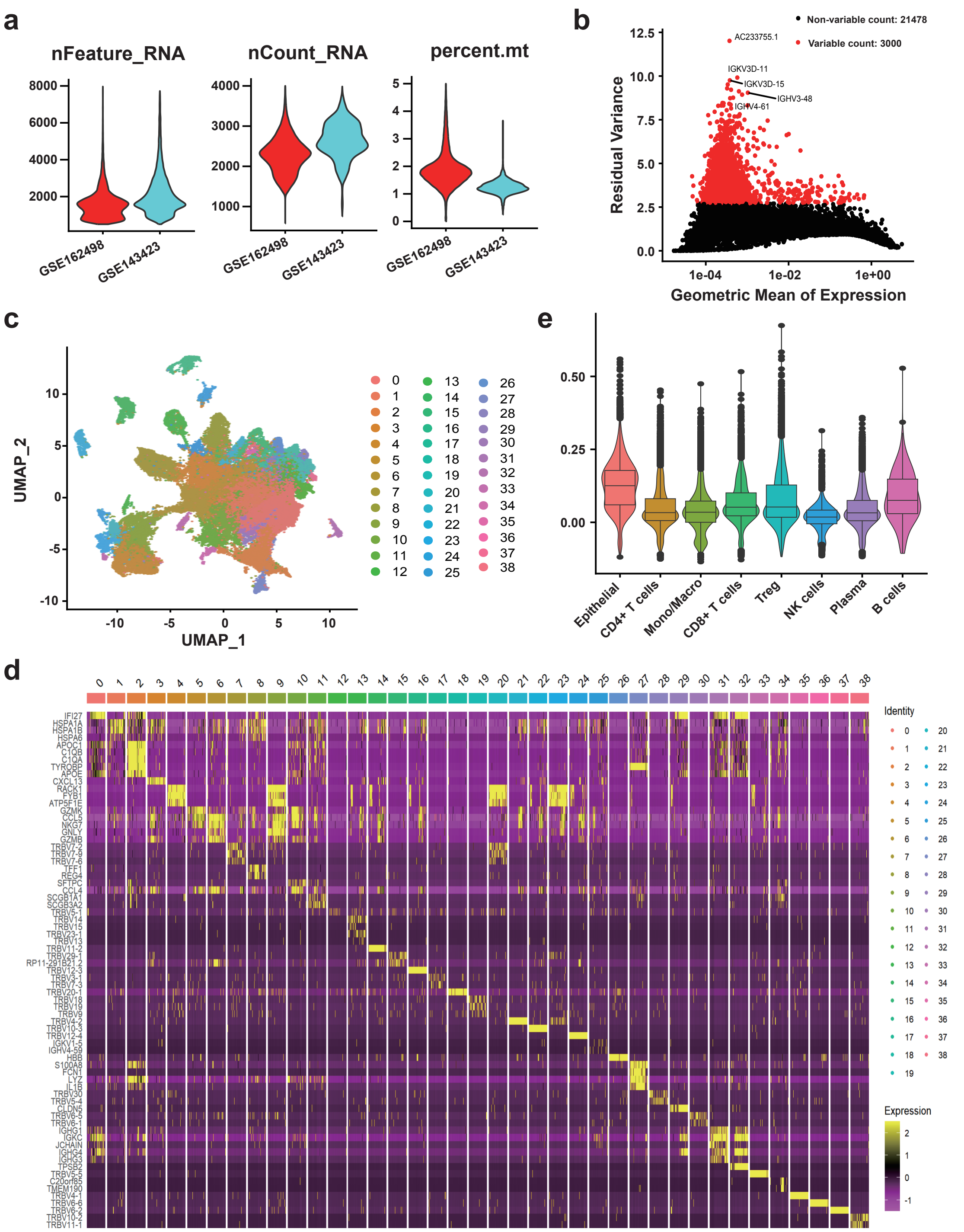

Supplementary Figure 5: Quality control of single-cell RNA sequencing (scRNA) data from two LUAD single-cell datasets. (a) The gene counts per cell (nFeature_RNA), number of unique molecular identifiers (UMIs) per cell (nCount_RNA), and percentage of mitochondrial genes per cell (percent. mt) of the scRNA-seq data. (b) The variance plot showed 21478 genes in all cells, red dots represent the top 3000 highly variable

genes. (c) UMAP plots for the 38 clusters' identification of single cells in LUAD datasets. (d) The Heatmap depicting expressions of the top 5 marker genes among 28 detected cell clusters. (e) Violin-box plot shows the level of CDIscore in eight cell types.

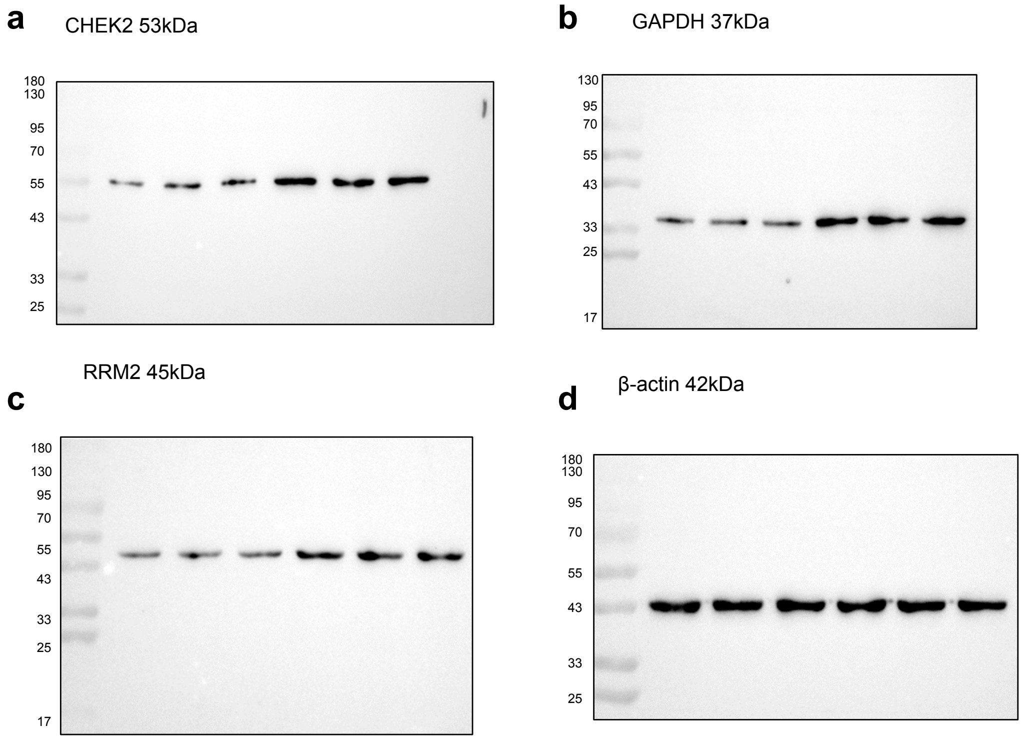

Supplementary Figure 6: Uncropped scans of the most important blots. (a) Uncropped scans of CHEK2. (b) Uncropped scans of GAPDH. (c) Uncropped scans of RRM2. (d) Uncropped scans of β-actin.

Supplementary Table 1 the primer sequence of each medel gene

Gene	Gene ID	primer sequence
CHEK2	11200	TCTCGGGAGTCGGATGTTGAG
		CCTGAGTGGACACTGTCTCTAA
KRT18	3875	TCGCAAATACTGTGGACAATGC
		GCAGTCGTGTGATATTGGTGT
MMP1	4312	GGGGCTTTGATGTACCCTAGC
		TGTCACACGCTTTTGGGGTTT
CHRNA5	1138	AAAGATGGGTTCGTCCTGTGG
		CAAACAAAACGATGTCTGGTGTC
TMPRSS4	56649	ATGCGGAACTCAAGTGGGC
		CTGTTTGTCGTACTGGATGCT
ITGB4	3691	GCTTCACACCTATTTCCCTGTC
		GACCCAGTCCTCGTCTTCTG
CD79a	973	CAAGAACCGAATCATCACAGCC
		TCTGCCATCGTTTCCTGAACA
CTLA4	1493	GCCCTGCACTCTCCTGTTTTT
		GGTTGCCGCACAGACTTCA
RRM2	6241	GTGGAGCGATTTAGCCAAGAA
		CACAAGGCATCGTTTCAATGG
GAPDH	2597	CTGGGCTACACTGAGCACC
		AAGTGGTCGTTGAGGGCAATG
ACTB	60	CATGTACGTTGCTATCCAGGC
		CTCCTTAATGTCACGCACGAT

Supplementary Table 2. The information of 2090 genes related to PCD

Apoptosis	Pyroptosis	Ferroptosis	Autophagy	Necroptosis	Cuproptosis	Parthanatos	Entotic cell death	Netotic cell death	Lysosome-dependent cell death	Alkaliptosis	Disulfidptosis	Oxeiptosis
AATF	BAK1	ABCC1	ABL1	GLUD1	FDX1	PARP	AMPK	ELANE	ABCA2	IKBKB	SLC7A11	PGAM5
ABL1	BAX	ACACA	ABL2	GLUD2	LIAS	MIF	ATG5	MMP1	ABCB9	NFKB1	SLC3A2	KEAP1
ACAA2	CASP1	ACO1	ACER2	ALOX15	LIPT1	AIFM1	ATG7	MPO	ACP2	CA9	RPN1	AIFM1
ACKR3	CASP3	ACSF2	ADRA1A	FTH1	DLD	HSP70	BECN1	CAMP	ACP5	CHUK	NCKAP1	NRF2
ACVR1	CASP4	ACSL1	ADRB2	PYG	DLAT	PAAN	CDC42	PADI4	ADGRE2	IKBKG	ZNF484	AIRE
ACVR1B	CASP5	ACSL3	AKT1	CAPN1	PDHA1	ARH3	CDH1	EIPA	AGA	NFKB1A	CCNC	
ADORA1	CASP6	ACSL4	AMBRA1	CASP1	PDHB	RNF146	CTNNA1	NCX1	AP1B1	RELA	MSH3	
AEN	CASP8	ACSL5	ATF6	GLNA	MTF1	ADPRHL2	CYBB	MIA	AP1G1		CCT5	
AGT	CASP9	ACSL6	ATG101	BAX	GLS	OGG1	MYH14		AP1M1		SKA1	
AGTR2	CHMP2A	AIFM2	ATG13	BCL2	CDKN2A		PI3KC3		AP1M2		ZCCHC14	
AIFM1	CHMP2B	AKR1C1	ATG14	FADD	GCSH		RHOA		AP1S1		SCGB3A2	
AKT1	CHMP3	AKR1C2	ATG2A	RIPK1	ATP7A		RNF146		AP1S2		HOXA2	
ANXA6	CHMP4A	AKR1C3	ATG2B	TNF	ATP7B		ROCK		AP1S3		RABL3	
APAF1	CHMP4B	ALOX12	ATG5	TNFRSF1A	SLC31A1		RUBCN		AP3B1		CPM	
APPL1	CHMP4C	ALOX15	ATG7	TRADD			UVRAG		AP3B2		C12orf75	
AR	CHMP6	ALOX5	ATM	TRAF2					AP3D1		WDR44	
ARHGEF2	CHMP7	ATG5	ATP13A2	PPIA					AP3M1		POU4F1	
ARL6IP5	CYCS	ATG7	ATP6V0A1	CAPN2					AP3M2		AKIRIN1	
ARMC10	ELANE	ATP5MC3	ATP6V0A2	HSP90A					AP3S1		C9orf72	
ARRB2	GPX4	BACH1	ATP6V0B	IL1A					AP3S2		LIX1	
ASAH2	GSDMB	CARS	ATP6V0C	TNFSF6					AP4B1		ELTD1	
ATF3	GSDMC	CBS	ATP6V0D1	TNFRSF6					AP4E1		KIF7	
ATF4	GSDMD	CD44	ATP6V0D2	CASP8					AP4M1		RPRD1A	
ATM	GSDME	CHAC1	ATP6V0E1	JNK					AP4S1		SERPINE2	
ATP2A1	GZMB	CISD1	ATP6V0E2	JAK2					ARF1		IPO9	
ATP2A3	HMGB1	CP	ATP6V1A	CAMK2					ARL8B		MTMR2	
ATP5IF1	IL18	CRYAB	ATP6V1B1	IL1B					ARSA		LONRF1	
AVP	IL1A	CS	ATP6V1B2	IFNG					ARSB		ARC	
BAD	IL1B	CYBB	ATP6V1C1	STAT3					ARSG		NRROS	
BAG3	IRF1	DPP4	ATP6V1C2	IRF9					ASAH1		MBOAT2	
BAG5	IRF2	EMC2	ATP6V1D	TNFSF10					ATP10B		RGAG4	
BAG6	NLRC4	FADS2	ATP6V1E1	TNFRSF10A					ATP13A2		TXNRD1	
BAK1	NLRP1	FANCD2	ATP6V1E2	TNFRSF10B					ATP6AP1		C6orf203	
BAX	NLRP2	FDFT1	ATP6V1G1	CFLAR					ATP6V0A1		DEFB119	
BBC3	NLRP3	FTH1	ATP6V1G2	XIAP					ATP6V0A2		FMO3	
BCAP31	NLRP6	FTL	ATP6V1H	BID					ATP6V0A4		EVI2B	
BCL10	NLRP7	FTMT	AUP1	AIFM1					ATP6V0B		KRT83	
BCL2	NOD1	G6PD	BAD	TRPM7					ATP6V0C		KRTAP20-3	
BCL2A1	PLCG1	GCLC	BAG3	IFNAR1					ATP6V0D1		ACAP2	
BCL2L1	PJVK	GCLM	BCL2	IFNAR2					ATP6V0D2		NUTF2	
BCL2L10	PRKACA	GLS2	BCL2L11	IFNGR1					ATP6V1H		ZNF547	
BCL2L11	PYCARD	GOT1	BECN1	IFNGR2					BLK		TMEM97	
BCL2L12	SCAF11	GPX4	BMF	TLR3					BLOC1S1		AGXT2	
BCL2L14	TINAP	GSS	BNIP3	TIRP					BLOC1S2		FAM186A	
BCL2L2	TNF	HMGCR	BNIP3L	IFNA					BORCS5		WDFY1	
BCL3	TP53	HMOX1	BOK	IFNB					BORCS6		JUP	
BCLAF1	TP63	HSBP1	C9orf72	TRIF					BTK		PDE5A	
BDKRB2	AIM2	HSPB1	CALCOCO2	VDAC1					C12orf4		UBL4B	
BDNF	GSDMA	IREB2	CAMKK2	SLC25A4S					CBL		SCN2B	
BECN1	IL6	KEAP1	CAPN1	PPID					CD164		CEP192	
BID	NOD2	LPCAT3	CAPNS1	CYLD					CD300A		HIST1H3B	
BIK	TIRAP	MAP1LC3A	CASP1	RIPK3					CD63		UGDH	
BIRC6		MAP1LC3B	CASP3	MLKL					CD68		MC4R	
BLOC1S2		MAP1LC3C	CDC37	TRAF5					CD84		MED25	
BMF		MT1G	CDK5	TLR4					CHGA		GAPT	
BMP4		NCOA4	CDK5R1	RBCK1					CLN3		MROH2B	
BMP5		NFE2L2	CHMP4A	HMGB1					CLN5		ISG20	
BMPR1B		NFS1	CHMP4B	JAK1					CLNK		SNX10	
BNIP3		NOX1	CISD2	JAK3					CLTA		SLCO1C1	
BNIP3L		NQO1	CLEC16A	TYK2					CLTB		CALB1	
BOK		NRF2	CLN3	STAT1					CLTC		CFDP1	
BRCA1		OTUB1	CLU	STAT2					CLTCL1		VPS35	
BRCA2		PCBP1	CPTP	STAT4					CLU		HRASLS	
BRSK2		PCBP2	CSNK2A2	STAT5A					CPLX2		PSMA6	
BTK		PEBP1	CTSA	STAT5B					CTNS		PLD3	
CAAP1		PGD	CTTN	STAT6					CTSA		EIF3F	
CASP1		PHKG2	DAP	H2A					CTSB		TM2D3	
CASP10		PRNP	DAPK1	TNFAIP3					CTSC		ACACB	
CASP12		PROM2	DAPK2	RNF31					CTSD		CMAS	
CASP2		PTGS2	DAPK3	CHMP2A					CTSE		ZNF20	
CASP3		RPL8	DAPL1	CHMP2B					CTSF		B2M	
CASP4		SAT1	DCN	VPS24					CTSG		TMC4	
CASP5		SAT2	DDIT3	CHMP4A					CTSH		CDKN2AIP	
CASP8		SLC11A2	DDRGK1	CHMP4B					CTSK		ADD1	
CASP8AP2		SLC1A5	DEPDC5	CHMP6					CTSL		BHMT2	
CASP9		SLC39A14	DEPP1	VPS4					CTSO		TOX4	
CAV1		SLC39A8	DHRSX	CHMP1					CTSS		SLC25A23	
CCAR2		SLC3A2	DNM1L	CHMP5					CTSV		ARHGAP31	
CCK		SLC40A1	DRAM1	SMPD1					CTSW		CCDC37	
CD14		SLC7A11	DRAM2	PYCARD					CTSZ		FNDC3B	
CD24		SQLE	EEF1A1	NLRP3					DEF8		UBE2D2	
CD27		STEAP3	EEF1A2	ZBP1					DNASE2		CDC23	
CD28		TF	EIF2AK4	IL33					DNASE2B		DDX5	
CD38		TFRC	EIF4G1	FTL					ENTPD4		THSD7B	
CD3E		TP53	EIF4G2	SQSTM1					FAM98A		RP11-93B14.6	
CD44		VDAC2	ELAPOR1	VDAC2					FER		PDCD6	
CD5		VDAC3	EP300	VDAC3					FES		GPX4	
CD70		ZEB1	EPM2A	CHMP7					FGR		RUVBL2	
CD74			ERCC4	PGAM5					FLCN		ZNF691	
CDIP1			ERN1	BIRC2					FOXF1		ANO8	
CDKN1A			EXOC1	BIRC3					FTH1		NOP10	
CDKN2D			EXOC4	EIF2AK2					FTL		BANF2	
CEBPB			EXOC7	PLA2G4					FUCA1		PFKFB3	
CFLAR			EXOC8	DNM1L					GAA		SPO11	
CHAC1			FBXL2	SPATA2					GAB2		OLFM1	
CHCHD10			FBXO7	FAF1					GALC		HIST4H4	
CHEK2			FBXW7	SHARPIN					GALNS		SCN11A	
CIB1			FEZ1	NOX2					GATA2		MEGF8	
CIDEB			FEZ2	USP21					GBA		FAM163B	
CLU			FLCN	PARP1					GCC2		USP19	
COA8			FOXK1	CHMP4C					GGA1		OR1N2	
COL2A1			FOXK2						GGA2		CXCL13	
CRADD			FOXO1						GGA3		SAP30BP	
CREB3			FOXO3						GLA		ATP6V0A2	
CREB3L1			FTH1						GLB1		PSMB4	
CRH			FTL						GM2A		APRT	
CRIP1			FYCO1						GNPTAB		POLR1D	
CSF2			FZD5						GNPTG		KRT73	
CSNK2A1			GAPDH						GNS		GPR61	
CSNK2A2			GATA4						GUSB		CPZ	
CTH			GBA						HDAC6		SPINT3	
CTNNA1			GFAP						HEXA		EFCAB7	
CTSC			GNAI3						HEXB		ZNF639	
CTTN			GOLGA2						HGS		HIST1H2BB	
CUL1			GPR137						HGSNAT		LSM4	
CUL2			GPR137B						HMOX1		LRRC10	
CUL3			GPSM1						HPS6		PLD2	

CUL4A
CUL5
CX3CL1
CX3CR1
CXCL12
CYLD
CYP1B1
DAB2IP
DAP
DAP3
DAPK1
DAPK2
DAPK3
DAPL1
DAXX
DBH
DCC
DDIAS
DDIT3
DDIT4
DDX3X
DDX47
DDX5
DEDD
DEDD2
DELE1
DEPTOR
DIABLO
DIDO1
DNAJA1
DNAJC10
DNM1L
DPF2
DYRK2
E2F1
E2F2
EDA2R
EIF2AK3
ELL3
ENO1
EP300
EPHA2
EPO
ERBB3
ERCC6
ERN1
ERN2
ERO1A
ERP29
EYA1
EYA2
EYA3
EYA4
FADD
FAF1
FAIM
FAIM2
FAM162A
FAS
FASLG
FASTK
FBH1
FBXW7
FEM1B
FGA
FGB
FGF10
FGFR1
FGFR3
FGG
FHIT
FIGNL1
FIS1
FNIP2
FXN
FYN
FZD9
G0S2
GABARAP
GATA1
GATA4
GCLM
GDNF
GFRAL
GGCT
GHITM
GNAI2
GNAI3
GPER1
GPX1
GRINA
GSDME
GSK3A
GSK3B
GSKIP
GSTP1
GZMB
HDAC1
HERPUD1
HGF
HIC1
HIF1A
HINT1
HIP1
HIP1R
HIPK1
HIPK2
HMGB2
HMOX1
HNRNPK
HRAS
HRK
HSPA1A
HSPA1B
HSPB1
HTRA2
HTT
HYAL2
HYOU1
GSK3A
GSK3B
HAX1
HDAC6
HERC1
HGF
HIF1A
HMGB1
HMOX1
HSP90AA1
HSPA8
HSPB1
HSPB8
HTR2B
HTRA2
HTT
HUWE1
IFI16
IFNG
IKBKG
IL10
IL10RA
IL4
IRGM
ITPR1
KAT5
KAT8
KDM4A
KDR
KEAP1
KIF25
KLHL22
KLHL3
LACRT
LAMP1
LAMP2
LAMP3
LAMTOR1
LAMTOR2
LAMTOR3
LAMTOR4
LAMTOR5
LARP1
LEP
LEPR
LGALS8
LRRK2
LRSAM1
LZTS1
MAP1LC3A
MAP1LC3B
MAP1LC3C
MAP3K7
MAPK15
MAPK3
MAPK8
MAPT
MCL1
MEFV
MET
MFN2
MFSD8
MID2
MIR199A1
MIRLET7B
MLST8
MT3
MTCL1
MTDH
MTM1
MTMR3
MTMR4
MTMR8
MTMR9
MTOR
NCOA4
NEDD4
NLRP6
NOD1
NOD2
NPC1
NPRL2
NRBP2
NUPR1
OPTN
ORMDL3
OSBPL7
PAFAH1B2
PARK7
PHB2
PHF23
PIK3C2A
PIK3C3
PIK3CA
PIK3CB
PIK3R2
PIM2
PINK1
PIP4K2A
PIP4K2B
PIP4K2C
PJVK
PLEKHF1
PLK2
PLK3
POLDIP2
PRKAA1
PRKAA2
PRKAB1
PRKAB2
PRKACA
PRKAG1
PRKAG2
PRKAG3
PRKD1
PRKN
PSAP
PTPN22
PYCARD
HSPA8
HYAL1
IDS
IDUA
IGF2R
IL13
IL13RA2
IL4
IL4R
KIF1B
KIT
KXD1
LAMP1
LAMP2
LAMP3
LAMTOR1
LAPTM4A
LAPTM4B
LAPTM5
LAT
LAT2
LGALS9
LGMN
LIPA
LRRK2
LYN
M6PR
MAN2B1
MANBA
MAP1LC3A
MAP6
MCOLN1
MFSD8
MILR1
MRGPRX2
MT3
MYH9
NAGA
NAGLU
NAGPA
NAPSA
NCOA4
NDEL1
NEDD4
NEU1
NPC1
NPC2
NR4A3
PDPK1
PIK3C3
PIK3CD
PIK3CG
PIP4K2A
PIP4K2B
PIP4P1
PLA2G15
PLA2G3
PLEKHM1
PLEKHM2
PPT1
PPT2
PSAP
PSAPL1
PTGDR
PTGDS
RAB34
RAB3A
RAB7A
RAC2
RUBCNL
S100A13
SCARB2
SGSH
SLC11A1
SLC11A2
SLC17A5
SMPD1
SNAP23
SNAPIN
SNX16
SNX4
SORL1
SORT1
SPAG9
SPHK2
SQSTM1
STXBP1
STXBP2
SUMF1
SYK
SYTL4
TCIRG1
TFEB
TMEM106B
TPP1
UNC13D
VAMP7
VAMP8
VPS33A
VPS33B
VPS4A
WASH3P
ZFYVE16
LMO7
CCT6A
APBA1
WFDC1
CER1
UBR4
DPP7
PTGES3L-AARSD1
OR1G1
PRKACA
FBXW5
PHGDH
DDIT4
KLHL11
HNRNPA1
SGCD
LRBA
AP3B1
FAM86B1
KCTD19
EXOC7
IFNA7
MSH6
SIRT7
MGAT2
FAM126A
XPOT
HIST1H3A
CASP14
C16orf96
TNFAIP2
LIPH
LTB
ALDH3A2
HEXDC
SIAE
SEMA3D
RNPS1
NEK11
SLC1A6
NPC1
PROL1
SDE2
IL1RAP
CENPH
ZNF296
CCDC149
TADA2A
GDPD3
NPTN
FBXW11
TMEM101
SUPT16H
LAMTOR1
CYLC1
ARL3
FBXW12
CCDC108
ITPA
TOMM6
SLC17A3
H3F3B
HACL1
ATP7B
PKIB
KLHL10
SS18
ZNF812
DIS3L2
TRPT1
PGRMC2
CDYL
OR8B12
ENGASE
RANBP3
SLC39A5
NOL8
ADAM22
SLC52A2
ZNF280D
HIST1H3E
PRKCSH
PFDN2
SLC7A6OS
POMZP3
PRMT3
DHODH
CEP85L
C12orf68
NSMF
TIMM17B
DDX52
BAI3
KLHL28
BACH2
PRKAG2
RPS11
MAP1LC3B
SLC8A3
SLFN14
SECISBP2L
NECAB2
ATP6AP1
FOSB
LACC1
LMOD1
ZNF221
SLC35E3
ZNF543
CBY3
CHRNA5
M6PR
FANCG
TRIB1
ABCC12
SPCS2
TMEM177
ACTR1A
ANKHD1-EIF4EBP3

ICAM1	QSOX1	ELMO3
IFI16	RAB39B	TCTN3
IFI27	RAB3GAP1	APEX2
IFI27L1	RAB3GAP2	TOMM22
IFI27L2	RAB7A	ZNF446
IFI6	RAB8A	ANTXR1
IFNB1	RALB	B4GALT3
IFNG	RASIP1	DCAF13
IGF1	RB1CC1	JUN
IKBKE	RETREG1	TXNDC9
IL12A	RETREG3	PEX16
IL19	RHEB	UBAC2
IL1A	RIPK2	HCN1
IL1B	RMC1	ASB10
IL2	RNF152	ST6GALNAC3
IL20RA	RNF41	FAM124A
IL33	RNF5	ZMAT1
IL4	ROCK1	ERVV-2
IL6R	RPTOR	OBP2A
IL7	RRAGA	VBP1
INCA1	RRAGB	TTLL2
ING2	RRAGC	CENPL
ING5	RRAGD	CCDC70
INHBA	RUBCN	TNMD
INHBB	RUFY4	EBP
INS	SCFD1	LYPD3
ITGA6	SCOC	GGA3
ITGAM	SEC22B	ASB9
ITGAV	SESN1	NOP16
ITM2C	SESN2	OR10AD1
ITPR1	SESN3	ANTXR2
ITPRIP	SH3BP4	HNRNPR
IVNS1ABP	SH3GLB1	AFAP1
JAK2	SIRT1	EIF6
JMY	SIRT2	TAS2R41
JUN	SLC38A9	MYPN
KDM1A	SMCR8	HIST1H4A
KITLG	SMG1	LRRK1
KRT18	SNCA	GEMIN2
KRT8	SNRNP70	GRN
LCK	SNX32	SAT2
LGALS12	SNX5	LRRC7
LGALS3	SNX6	GDPD1
LRRK2	SOGA1	CYB5B
LTBR	SOGA3	URB2
LY96	SPTLC1	ACADVL
MADD	SPTLC2	PKD2
MAEL	SQSTM1	UQCRB
MAGEA3	SREBF1	NGLY1
MAP2K5	SREBF2	CISD1
MAP3K5	STAT3	NAPB
MAPK7	STBD1	CCL7
MAPK8	STING1	COL4A3BP
MAPK8IP1	STK11	CYHR1
MAPK8IP2	STUB1	KIAA0232
MAPK9	SUPT5H	PIGK
MARCHF7	SVIP	TMPRSS4
MAZ	SYNPO2	RBMX2
MCL1	TAB2	TMEM168
MDM2	TAB3	WDR7
MELK	TBC1D14	MRPL4
MFF	TBC1D25	DAZAP1
MIF	TBK1	R3HCC1L
MIR132	TEX264	C12orf66
MIR15A	TFEB	PELI2
MIR16- 1	TICAM1	CYB561D1
MIR17	TIGAR	LEFTY1
MIR198	TLK2	PRKG2
MIR21	TMEM150A	GBAS
MIR210	TMEM150B	KIAA1731
MIR221	TMEM150C	MAP2
MIR222	TMEM39A	YPEL1
MIR26B	TMEM39B	WDR87
MIR27B	TMEM59	SLC25A38
MIR449A	TOMM7	NXT1
MKNK2	TP53	GABPB2
MLH1	TP53INP1	OSCP1
MLLT11	TP53INP2	RFC2
MMP9	TPCN1	TNC
MNT	TPCN2	BTG2
MOAP1	TREM2	TCF4
MPV17L	TRIB3	SCAMP4
MSH2	TRIM13	STAM2
MSH6	TRIM14	CCDC61
MSX1	TRIM21	ZNF259
MUC1	TRIM22	FOXP4
MUL1	TRIM27	WNT9A
MYBBP1A	TRIM34	MYRIP
NACC2	TRIM38	KIF18A
NANOS3	TRIM5	CDK18
NBN	TRIM6	SLC46A3
NCK1	TRIM65	TLE4
NCK2	TRIM68	HEXB
NDUFA13	TRIM8	COG5
NDUFS3	TRIML1	NRBP1
NFATC4	TRIML2	FAM89B
NFE2L2	TSC1	PRRG1
NGF	TSC2	ASB6
NGFR	TSPO	RPL36A
NKX3- 1	UBA5	INHBC
NLE1	UBQLN1	RRP1
NME5	UBQLN2	RAVER1
NMT1	UBQLN4	PCLO
NOC2L	UCHL1	KRT72
NOG	UFC1	UCHL1
NOL3	UFL1	DIS3L
NONO	UFM1	ZNF182
NOS3	ULK1	ZBTB8A
NOX1	USP10	KIAA1551
NR4A2	USP13	CHD1L
NUPR1	USP30	MSX1
OPA1	USP33	CEPT1
P2RX4	USP36	RTF1
P2RX7	UVRAG	CNIH4
P4HB	VDAC1	IFI44
PAK2	VPS13C	IL2
PAK5	VPS13D	RNF14
PARK7	VPS26A	SLC6A1
PARP1	VPS26B	ZFP30

PARP2
PAWR
PCGF2
PDCD10
PDCD5
PDCD6
PDIA3
PDK1
PDK2
PDPK1
PDX1
PEA15
PELI3
PERP
PF4
PHIP
PHLDA3
PIAS4
PIDD1
PIH1D1
PIK3R1
PINK1
PLAGL2
PLAUR
PLEKHF1
PLSCR3
PMAIP1
PML
POLB
POU4F1
POU4F2
PPARD
PPIA
PPIF
PPM1F
PPP1CA
PPP1R13B
PPP1R15A
PPP2R1B
PPP3CC
PPP3R1
PRDX2
PRELID1
PRKCA
PRKCD
PRKDC
PRKN
PRKRA
PRODH
PSEN1
PSMD10
PSME3
PTEN
PTGIS
PTH
PTPMT1
PTPN1
PTPN2
PTPRC
PTTG1IP
PYCARD
QARS1
RACK1
RAF1
RB1
RB1CC1
RBCK1
RELA
RET
RFFL
RHOT1
RHOT2
RIPK1
RIPK3
RNF183
RNF186
RNF34
RNF41
RPL11
RPL26
RPS27L
RPS3
RPS6KB1
RPS7
RRP8
RTKN2
RTL10
S100A8
S100A9
SCG2
SCN2A
SCRT2
SELENOK
SELENOS
SENP1
SEPTIN4
SERINC3
SERPINE1
SFN
SFPQ
SFRP1
SFRP2
SGMS1
SGPL1
SGPP1
SH3RF1
SHH
SHISA5
SIAH1
SIAH2
SIRT1
SIVA1
SKIL
SLC25A5
SLC35F6
SLC9A3R1
SMAD3
SNAI1
SNAI2

VPS35
WAC
WASHC1
WDFY3
WDR24
WDR41
WDR6
WDR81
WIPI2
ZC3H12A
ZKSCAN3
ZMPSTE24

ZDHHC23
EID1
F7
CST7
SPATA21
ZNF280A
GTF2F1
IDH2
RNF32
CDC25B
CTIF
TEAD2
PROX1
SRSF6
LAX1
MED16
STAT3
PDZK1
LYPD4
CES3
AMACR
PDAP1
CCM2
PEX5L
FAM210A
PAPSS1
SNX12
C20orf202
MICU2
LSM5
PF4
ZNF506
RD3L
HOXB13
CCDC158
FAM166A
C12orf61
DYNC1H1
UTS2B
TM2D2
CAPSL
TNN
UBAC1
FABP12
GTF2E2
EML6
GP9
TCF20
NOB1
TMED4
TRIM47
GNG12
C15orf60
CYLC2
ATPIF1
EXT1
YARS
NMNAT2
GARNL3
DEFB108B
KRCC1
SERPINE3
PTGR2
SHISA6
FEM1C
TRA2A
MDP1
SNRPB2
TTC17
PKIA
AP1M1
DHRS11
IFI27L2
CRCP
HIST1H2BM
CLDN18
AMPD1
OR51I2
CDK6
AFF1
ITGB8
RPS21
MCPH1
AP1G1
DDN
ACOT2
HIST2H2AC
LRRC32
HS6ST2
TFAP2C
RBMS2
LAPTM5
TSACC
FBXL20
ZNF317
TWIST2
TNXB
PNN
ZRANB1
EIF4B
RBM34
HCAR3
TMPRSS3
TMX2
SERPINB1
FAM161A
EFCC1
OR1A1
CENPW
ZNF697
RAB9B
SH2D2A
TMEM88
TNFRSF17
UBP1
CORO1C
C20orf195
DIXDC1
IGSF9

SNW1
SOD1
SOD2
SORT1
SP100
SRC
SRPX
SST
SSTR3
ST20
STK11
STK24
STK25
STK3
STK4
STRADB
STX4
STYXL1
SYVN1
TAF9
TAF9B
TCF7L2
TERT
TFDP1
TFDP2
TFPT
TGFB1
TGFB2
TGFBR1
THBS1
TICAM1
TICAM2
TIMM50
TIMP3
TLR3
TLR4
TM2D1
TMBIM1
TMBIM6
TMC8
TMEM102
TMEM109
TMEM117
TMEM14A
TMEM161A
TNF
TNFAIP3
TNFRSF10A
TNFRSF10B
TNFRSF10C
TNFRSF12A
TNFRSF1A
TNFRSF1B
TNFRSF25
TNFSF10
TNFSF12
TOPORS
TP53
TP53BP2
TP63
TP73
TPD52L1
TPT1
TRADD
TRAF1
TRAF2
TRAF7
TRAP1
TRIAP1
TRIB3
TRIM32
TRIM39
TXNDC12
TYROBP
UACA
UBB
UBE2K
UBE4B
UBQLN1
UMOD
UNC5B
URI1
USP28
USP47
VDAC2
VNN1
WDR35
WNT4
WWOX
XBP1
YAP1
YBX3
YWHAB
YWHAE
YWHAG
YWHAH
YWHAQ
YWHAZ
ZC3HC1
ZDHHC3
ZMYND11
ZNF205
ZNF385A
ZNF385B
ZNF622
ZSWIM2

AQR
CACFD1
IMPG2
PMVK
ZFP90
PGLYRP4
AKAP14
KLF3
PRELID1
VPS18
SQLE
PSME1
C20orf112
KATNBL1
C8orf37
MPZ
CYP2R1
C7orf55
PEX14
BCL2L2
C7orf50
RRP12
ZNF160
TRHR
NCAPH
ZC3H7B
WDR70
RSPH3
HSPB7
METTL13
C21orf33
DPYS
EIF4EBP3
WBSCR28
ABCG1
TSR1
FBXL2
SMAD4
KRT78
AKT2
MXD3
ADAM15
C1orf74
ATXN1L
SLC29A3
LHFPL3
CA11
DEGS1
WFDC3
ZNF606
ARHGEF7
GPR125
APIP
JDP2
ARHGAP12
MED12
MUC22
NUP133
TMCO5A
MAPK8IP1
SNRK
GNAT2
ATP5L2
NDST1
TMED1
KRTAP10-9
SMIM9
TPI1
GINS1
ANKRD39
TYROBP
KDR
USMG5
FAM219B
SOAT2
P4HA3
ZNF181
TOMM5
PLSCR4
ZNF678
GABRG3
TBC1D13
NUP43
COL4A3
STMN4
SERPINA4
IWS1
WFDC8
BCLAF1
ATG13
MLLT10
DTNB
CIB4
SEMA5B
NFATC4
ITGB4
C19orf45
ATRX
PNPLA1
RP11-389E17.1
STMN3
CD79A
TRIM69
DPPA5
GALNTL6
RAB32
SRRM5
RPP14
GATA5
SPHKAP
EPM2AIP1
RAB4A
EGFL6
CGREF1
C3orf14
RPLP0
GDI1
RNF220
ZNF865

POLR2C
FDXACB1
KRTAP4-3
TBX18
ZNF180
JKAMP
LAIR1
SPTSSA
MYH14
DET1
EBI3
RNF26
CTBP1
SLAIN2
PLEKHA5
TNPO3
STK16
DIEXF
C7orf62
45261
KRT222
HLCS
TGIF1
NECAP1
SYT5
FEM1A
FGF3
ZNF766
NRF1
DIP2B
TXN
44996
ANKRD16
DCN
C9orf117
ZDHHC4
UNC50
PIFO
NTN1
BCAS3
IL6ST
IGF2BP2
PRKRIR
REG3A
PEX12
POLR1B
RNASE2
FAM155B
DDI1
PIGL
RAD23B
GPR88
MYOZ3
SYNE2
CCL8
PIK3IP1
ZCCHC4
ELL3
C2orf47
PCYOX1
SARM1
ATP6V1A
ZNF749
ZNF449
ZEB1
SAV1
NKTR
TMPRSS11D
RNASE13
FAM150A
PIGW
OCA2
SFI1
GLO1
PCDHGA5
ZNF565
PEX6
GPC1
DHRS13
ART1
GRHPR
SDR42E1
DNMT1
LTA
EXOC1
RPS12
TAL2
CST9L
TAPT1
KLF1
GRAMD1A
HLA-B
NAV1
C16orf92
AFTPH
FAM83E
CLIP2
CXorf57
FUBP1
UBE2E1
AK5
ZNF653
TNRC6B
RAB37
OR52N1
SEC14L6
ZNF23
SULF1
C7orf41
STK17A
TARM1
RAB2A
CHST4
MAT2A
SLC35G2
UCMA
AFF2
TAX1BP3
COG2

PIK3CD
NME2
HIST1H2AB
TAGLN
MIP
SCRN1
ATAD3A
DDX1
C15orf53
KRTAP10-4
MMD2
MTHFS
ARSD
DCLRE1C
HSP90AB1
ITGAE
PWP2
HIST1H2AD
LRP5
USP18
RETNLB
CNTRL
HOXD9
CNTNAP1
COPE
PSD3
ZNF10
OSGIN2
EBLN1
TMEM44
DOK4
TRAF1
CENPO
SESN1
TAF9
RBBP7
ZNF419
DHX15
GML
CD97
RFWD3
RER1
TNFSF10
CORO2B
ICOSLG
MAP3K1
RGS6
ZNF407
CIRBP
SMYD2
FOLR3
C17orf85
STPG2
TSSC4
FZD1
MTMR4
AGBL3
GTF3C3
TTLL11
UBXN2A
TGFBR2
CLIC5
ACSM5
OSCAR
TMEM57
MKI67IP
KIAA0087
CCDC30
ECI1
TM9SF4
INF2
CDRT1
HM13
RAP1B
GSTA5
KATNAL2
MAS1L
CACNA1C
CDT1
HDGFL1
GDAP2
SYCP1
STK3
BPI
GPR15
C7orf57
FAM217A
CDC42BPA
APOL4
TBC1D14
TTF2
LEPROTL1
HS3ST3A1
EDF1
WDR20
TMEM41A
FAM120B
IQCK
CENPI
PAK1IP1
CWC22
AEN
LSM7
GSK3B
ADAMTSL3
STK32C
POLE2
ESPNL
LILRA2
IFT88
KBTBD4
PLEKHO1
MBOAT1
MSANTD2
VCP
BFAR
NFIC
GAR1
ENDOV

CCDC144NL
UBE3C
PRPF3
OR9Q2
IFI35
ELF2
CCDC42B
CCDC176
ZBTB44
GRPEL1
PDC
ANKLE2
IGF2R
NRARP
ATP1A3
CSNK2B
NSF
PSMB6
MS4A13
PARD3
SND1
DND1
WBP5
OR6C74
KAL1
MTPN
ADAM17
SFN
POFUT1
MPND
MAGED1
NKRF
ATP5L
KCNMB2
CERS3
CTLA4
ZFP62
ZNF155
GTF2H1
SBF2
NT5M
CCNE1
FRG1
AQP6
ZBED4
GRB2
SERPINA3
ADM
AMZ2
CD200R1
C8B
IQGAP2
OR2M5
TTC4
TMEM102
INPP4A
RPS3A
RRM2
LPCAT3
WIBG
ANGPTL6
HIGD2B
PRKD1
FAM186B
PROSC
EMILIN3
TMEM219
MRPS36
TCEA3
KCNJ6
SNN

Supplementary Table 3 the information of 52 PCD genes in LUAD

gene_name	log2FoldCha	lfcSE	stat	pvalue	padj
BIK	2.00106575	0.16494956	12.1313796	7.2026E-34	1.8063E-32
BRCA1	1.47609135	0.1298307	11.3693554	5.9425E-30	1.1945E-28
CHEK2	1.47900828	0.11448219	12.9191119	3.5117E-38	1.1144E-36
E2F1	1.45776262	0.14270982	10.2148726	1.701E-24	2.4179E-23
E2F2	2.33705661	0.14668775	15.9321871	3.7887E-57	3.1126E-55
ENO1	1.18092634	0.0969699	12.1782778	4.0576E-34	1.0333E-32
GGCT	1.70031974	0.10841757	15.683065	1.9749E-55	1.5002E-53
ITM2C	1.19768671	0.11930083	10.0392153	1.0249E-23	1.3857E-22
KRT18	1.1940012	0.13106233	9.11017848	8.2247E-20	8.3628E-19
KRT8	1.27035247	0.13196229	9.62663268	6.1719E-22	7.3722E-21
MELK	3.61234179	0.17091169	21.1357206	3.734E-99	2.2388E-96
MLLT11	2.11705228	0.23467274	9.02129602	1.8588E-19	1.8361E-18
MMP9	1.99249621	0.20775452	9.59062751	8.7551E-22	1.0344E-20
PDK1	1.70997329	0.11719393	14.5909714	3.206E-48	1.7387E-46
PMAIP1	1.64251139	0.17565899	9.35056828	8.7178E-21	9.5872E-20
PPIF	1.07564269	0.09290903	11.5773752	5.3664E-31	1.1462E-29
RNF186	5.02292709	0.41123819	12.2141553	2.612E-34	6.7214E-33
TNFRSF25	1.93587869	0.15253312	12.6915301	6.5886E-37	1.9749E-35
GSDMB	1.72832504	0.14655752	11.7928102	4.2512E-32	9.6566E-31
AIM2	2.81494372	0.23369958	12.0451382	2.0573E-33	5.0425E-32
CP	3.48864787	0.2213479	15.7609259	5.778E-56	4.4813E-54
FANCD2	1.54039002	0.09936755	15.501943	3.3656E-54	2.416E-52
NQO1	3.03745694	0.21520204	14.1144432	3.0945E-45	1.4315E-43
PROM2	2.60571419	0.15844105	16.4459542	8.9661E-61	8.2867E-59
SLC7A11	3.06709536	0.23907255	12.8291405	1.1261E-37	3.5057E-36
EEF1A2	6.35492205	0.32779182	19.3870672	9.9236E-84	2.5301E-81
GAPDH	1.66196889	0.12132864	13.6980759	1.0425E-42	4.2006E-41
TRAF5	1.3295247	0.11439563	11.6221629	3.1798E-31	6.8712E-30
JAK3	1.10929702	0.11317958	9.80121167	1.1124E-22	1.406E-21
CDKN2A	3.56299729	0.25871076	13.7721264	3.7501E-43	1.5592E-41
MMP1	4.92906306	0.28866086	17.0756197	2.2542E-65	2.5446E-63
BLK	1.24472433	0.22844929	5.44858051	5.0773E-08	1.8939E-07
CXCL13	2.64081676	0.24769246	10.6616761	1.538E-26	2.4757E-25
CHRNA5	3.63368293	0.23142646	15.7012424	1.4831E-55	1.1324E-53
TMEM177	1.57023601	0.08119442	19.3392115	2.5129E-83	6.0917E-81
DCAF13	1.33993053	0.09251592	14.4832426	1.5463E-47	8.0703E-46
TMPRSS4	4.82128759	0.21660475	22.2584577	9.341E-110	1.219E-106
TNC	1.55322054	0.21252172	7.30852613	2.7009E-13	1.6737E-12
HS6ST2	3.68510737	0.19440693	18.9556377	3.9672E-80	8.341E-78
IGSF9	3.23840393	0.16456882	19.6781138	3.3214E-86	9.823E-84
GINS1	3.03731533	0.14949818	20.3167375	9.1456E-92	3.8276E-89
ITGB4	1.67562812	0.1840665	9.10338454	8.756E-20	8.8827E-19
CD79A	2.01179515	0.21099868	9.53463385	1.5042E-21	1.7423E-20
CGREF1	4.2381889	0.24986005	16.9622513	1.5624E-64	1.7114E-62
FAM155B	3.20183609	0.23512578	13.6175458	3.1498E-42	1.2366E-40
SULF1	2.5454902	0.18184263	13.9983141	1.5961E-44	7.0738E-43

CENPO	1.10082225	0.10011498	10.9955796	4.0133E-28	7.1704E-27
CDT1	2.75137083	0.15787966	17.4270126	5.1465E-68	6.5987E-66
POLE2	2.34753114	0.13736272	17.0900159	1.7613E-65	2.0035E-63
CTLA4	1.03014025	0.15961947	6.45372537	1.0913E-10	5.3574E-10
CCNE1	3.28161507	0.1847596	17.76154	1.4033E-70	2.0411E-68
RRM2	3.19820731	0.1608815	19.8792735	6.1518E-88	2.0067E-85

Supplementary Table 4 the C-index of each model

TCGA	TCGA	GSE31210	GSE50081	GSE72094
Lasso	0.66576869	0.54465146	0.62315975	0.61824201
RSF+Lasso	0.66576869	0.54465146	0.62315975	0.61824201
RSF+Enet[alp	0.665707	0.54127851	0.62546693	0.61923523
Enet[alpha=0	0.66593321	0.54336653	0.62294001	0.61916901
RSF+Enet[alp	0.66593321	0.54336653	0.62294001	0.61916901
Enet[alpha=0	0.66568644	0.54433023	0.62294001	0.61837444
RSF+Enet[alp	0.66568644	0.54433023	0.62294001	0.61837444
CoxBoost+En	0.67496093	0.55187922	0.61590859	0.60857474
Enet[alpha=0	0.6662828	0.53822679	0.62656559	0.61940076
RSF+Enet[alp	0.6662828	0.53822679	0.62656559	0.61940076
CoxBoost+En	0.67498149	0.55155798	0.61579873	0.6079457
CoxBoost+Ri	0.67962902	0.54850626	0.6145902	0.60741599
CoxBoost	0.66617998	0.54481208	0.62272028	0.61595762
Enet[alpha=0	0.66387678	0.54240283	0.62294001	0.62029465
CoxBoost+La	0.67919717	0.55316415	0.61019556	0.60632346
Lasso+StepC	0.66626224	0.55701895	0.6197539	0.60562821
CoxBoost+St	0.66626224	0.55701895	0.6197539	0.60562821
RSF+Enet[alp	0.66679691	0.53356891	0.62997144	0.61691773
CoxBoost+St	0.67014889	0.54914873	0.61228302	0.60533024
Enet[alpha=0	0.66095665	0.53292644	0.61711712	0.62393643
GBM	0.77985934	0.58801799	0.53911228	0.52178447
Lasso+StepC	0.66346549	0.56103437	0.61535926	0.58814766
StepCox[both	0.66764004	0.54449085	0.61338167	0.59731833
StepCox[both	0.66788681	0.54465146	0.61283235	0.59711968
StepCox[both	0.65853007	0.54449085	0.61557899	0.60350935
StepCox[back	0.65853007	0.54449085	0.61557899	0.60350935
StepCox[back	0.6677223	0.54465146	0.61228302	0.59665618
StepCox[back	0.66794851	0.54465146	0.61107449	0.59629201
StepCox[both	0.66745496	0.54449085	0.61107449	0.5962589
StepCox[back	0.66745496	0.54449085	0.61107449	0.5962589
StepCox[back	0.66745496	0.54416961	0.61107449	0.59642443
StepCox[both	0.66776343	0.54465146	0.60986596	0.59635822
StepCox[both	0.66722876	0.54449085	0.61041529	0.59596093
StepCox[both	0.66731101	0.54449085	0.61019556	0.59592783
StepCox[both	0.66835979	0.54465146	0.60964623	0.59513326
StepCox[both	0.66755779	0.54449085	0.60964623	0.59592783
StepCox[back	0.66745496	0.54449085	0.60964623	0.59602715
StepCox[back	0.66735214	0.54416961	0.60997583	0.59602715
StepCox[back	0.66735214	0.54449085	0.6094265	0.59546433
StepCox[back	0.6682981	0.54481208	0.60964623	0.59377587
StepCox[back	0.66724932	0.54433023	0.60953637	0.59536501
StepCox[both	0.66739327	0.54433023	0.60931663	0.59539811
StepCox[back	0.66739327	0.54433023	0.60931663	0.59539811
StepCox[back	0.66729045	0.54433023	0.60920677	0.59539811
StepCox[both	0.66731101	0.54433023	0.60898704	0.59553054
StepCox[back	0.66747553	0.54416961	0.60898704	0.59543122

StepCox[bot	0.66747553	0.54416961	0.60898704	0.59539811
StepCox[bot	0.66745496	0.54416961	0.60887717	0.59546433
RSF+StepCc	0.66706424	0.54433023	0.60799824	0.5953319
RSF+StepCc	0.66706424	0.54433023	0.60799824	0.5953319
StepCox[bot	0.66706424	0.54433023	0.60799824	0.5953319
StepCox[bac	0.66706424	0.54433023	0.60799824	0.5953319
RSF+GBM	0.79587892	0.59139094	0.50043946	0.5223804
RSF+Enet[al	0.66087439	0.46980405	0.61810591	0.62403576
CoxBoost+C	0.79044995	0.49365564	0.53883762	0.51014733
StepCox[bot	0.77535576	0.49365564	0.53845309	0.51082602
StepCox[bac	0.77031751	0.49365564	0.53845309	0.51046184
Lasso+GBM	0.7963519	0.49365564	0.51054713	0.50554544
SuperPC	0.57304434	0.48715066	0.57547792	0.62264526
RSF+SuperF	0.61071811	0.48168969	0.57734564	0.54259229
survivalSVM	0.53539113	0.49469965	0.54372665	0.60440324
RSF+surviva	0.53539113	0.49469965	0.54372665	0.60440324
StepCox[bac	0.53495928	0.48939929	0.55603164	0.58652541
CoxBoost+s	0.54217735	0.48747189	0.51087673	0.62214865
Lasso+survi	0.54217735	0.48747189	0.51087673	0.62214865
StepCox[bot	0.52033808	0.48988114	0.54207866	0.58854494
StepCox[bac	0.52033808	0.48988114	0.54207866	0.58854494
CoxBoost+S	0.57964547	0.48297462	0.53900242	0.49084589
Lasso+Supe	0.57964547	0.48297462	0.53900242	0.49084589
StepCox[bot	0.54700995	0.47767427	0.52208306	0.52418474

Supplementary Table 5 GSVA results of each cohort

GSVA enrichment HALLMARK functions between high and low-PCDscoregroup in TCGA-LUAD

Tag	logFC	AveExpr	t	P.Value	adj.P.Val	B
G2M_CHECKPOINT	0.42998304	-0.0113618	12.6597597	4.34E-32	2.17E-30	61.9658964
GLYCOLYSIS	0.24251821	-0.0075697	12.3299912	1.03E-30	2.57E-29	58.8253401
MITOTIC_SPINDLE	0.30093883	-0.0047231	12.0151667	2.03E-29	3.38E-28	55.8687043
E2F_TARGETS	0.4334935	-0.0149688	11.7240958	3.07E-28	3.84E-27	53.1729615
MTORC1_SIGNALING	0.30668872	-0.0095757	11.3986298	6.15E-27	6.15E-26	50.2036044
MYC_TARGETS_V1	0.35919288	-0.0116238	9.9626753	1.84E-21	1.53E-20	37.7234268
MYC_TARGETS_V2	0.35153665	-0.0169624	9.73714142	1.21E-20	8.61E-20	35.8631201
UNFOLDED_PROTEIN_RESPONSE	0.22773726	-0.0064129	7.94971935	1.23E-14	7.72E-14	22.2122447
BILE_ACID_METABOLISM	-0.1239946	-0.0011298	-7.7473424	5.20E-14	2.89E-13	20.7985643
PI3K_AKT_MTOR_SIGNALING	0.15120251	-0.0073391	7.59457627	1.51E-13	7.55E-13	19.7504499
HYPOXIA	0.14897921	-0.0033061	7.4873665	3.16E-13	1.44E-12	19.0247976
DNA_REPAIR	0.1951959	-0.0067379	6.92268604	1.35E-11	5.64E-11	15.3408238
KRAS_SIGNALING_DN	-0.1260078	0.01010012	-6.4612133	2.45E-10	9.43E-10	12.5080213
PROTEIN_SECRETION	0.18100196	-0.0087568	6.24340183	9.10E-10	3.25E-09	11.2283771
ANDROGEN_RESPONSE	0.11459792	-0.0059022	5.39597773	1.05E-07	3.50E-07	6.61338642
UV_RESPONSE_UP	0.08345384	-0.0055775	5.12888863	4.16E-07	1.30E-06	5.28243715
REACTIVE_OXYGEN_SPECIES_PATHWAY	0.14434185	-0.0117754	5.00779339	7.63E-07	2.24E-06	4.69903531
CHOLESTEROL_HOMEOSTASIS	0.10623138	-0.0105162	4.83578366	1.76E-06	4.90E-06	3.89207295
ESTROGEN_RESPONSE_LATE	0.06875318	-0.007343	4.81700295	1.93E-06	5.08E-06	3.80551937
MYOGENESIS	-0.0948045	0.01335622	-4.6605996	4.04E-06	9.81E-06	3.09667202
APOPTOSIS	0.09373766	0.00393648	4.65633405	4.12E-06	9.81E-06	3.07764014
TGF_BETA_SIGNALING	0.11946048	0.00495005	4.35734017	1.60E-05	3.63E-05	1.78363209
OXIDATIVE_PHOSPHORYLATION	0.13485073	-0.0193127	3.65428447	0.00028493	0.00061941	-0.9425714
APICAL_JUNCTION	0.07593965	0.00695298	3.6378114	0.00030328	0.00063183	-1.0010349
PANCREAS_BETA_CELLS	-0.0939544	0.00324361	-3.5080215	0.00049183	0.00098366	-1.4528837
SPERMATOGENESIS	0.05428515	-0.0057223	3.00166792	0.00281817	0.00541955	-3.0653778
ADIPOGENESIS	0.06602383	-0.0057969	2.88314184	0.00410549	0.00760276	-3.4079259
TNFA_SIGNALING_VIA_NFKB	0.08016855	0.00163177	2.81181799	0.00511819	0.00913962	-3.607622
ESTROGEN_RESPONSE_EARLY	0.04424839	-0.0009256	2.75661871	0.00605192	0.01043243	-3.7588438
P53_PATHWAY	0.04974804	0.00139366	2.74540434	0.00625946	0.01043243	-3.7892108
ALLOGRAFT_REJECTION	-0.0848171	0.0087736	-2.5106537	0.01236269	0.01993982	-4.3972388
COAGULATION	-0.0506896	0.00809967	-2.3386001	0.01974601	0.03085314	-4.8092199
EPITHELIAL_MESENCHYMAL_TRANSITION	0.07868264	0.00266501	2.18576903	0.02929182	0.04438154	-5.1511681
APICAL_SURFACE	0.04443095	0.0070855	2.13203239	0.03348694	0.0492455	-5.2660148
KRAS_SIGNALING_UP	-0.0370139	0.01123759	-1.5994111	0.11035575	0.15458011	-6.251914
ANGIOGENESIS	0.04742117	0.01004451	1.59518433	0.11129768	0.15458011	-6.2586256
HEDGEHOG_SIGNALING	-0.0398238	0.01279106	-1.5514624	0.12141842	0.16407895	-6.327018
INTERFERON_ALPHA_RESPONSE	0.05717493	0.00368903	1.53022236	0.1265889	0.16656434	-6.3595635
NOTCH_SIGNALING	0.03039098	0.00995452	1.36722344	0.17216455	0.22072379	-6.5945156
IL6_JAK_STAT3_SIGNALING	-0.0388118	0.01547518	-1.3143251	0.18933447	0.23666809	-6.6651264
FATTY_ACID_METABOLISM	0.02847593	-0.0150587	1.26919339	0.20495772	0.24994844	-6.7231825
INFLAMMATORY_RESPONSE	-0.0299335	0.01428739	-1.0410861	0.29833461	0.35516025	-6.9857468
IL2_STAT5_SIGNALING	-0.0179283	0.00960439	-0.8588946	0.39080684	0.45442655	-7.1583645
UV_RESPONSE_DN	0.01510063	0.00811982	0.61950097	0.53586625	0.60893892	-7.3349738
XENOBIOTIC_METABOLISM	-0.0046306	-0.0050399	-0.2714782	0.7861344	0.87348267	-7.4898098
WNT_BETA_CATENIN_SIGNALING	-0.0053808	0.01080693	-0.244905	0.80662968	0.87677139	-7.4966639
HEME_METABOLISM	0.00319401	-0.0026309	0.19988756	0.84164912	0.8953714	-7.5066659
INTERFERON_GAMMA_RESPONSE	0.00392394	0.00991427	0.1146049	0.90880393	0.94272364	-7.520064
COMPLEMENT	0.00225769	0.00858394	0.09560892	0.92386917	0.94272364	-7.5220589
PEROXISOME	0.00036487	-0.0055697	0.02029711	0.98381439	0.98381439	-7.5264196

GSVA enrichment HALLMARK functions between high and low-PCDscoregroup in GSE50081

Tag	logFC	AveExpr	t	P.Value	adj.P.Val	B
MYC_TARGETS_V2	0.40454067	-0.0218388	10.1125869	1.82E-19	9.08E-18	33.5068961
E2F_TARGETS	0.4542404	-0.0324492	9.93925806	5.69E-19	1.42E-17	32.3768029
G2M_CHECKPOINT	0.39131737	-0.0223526	9.27730921	4.23E-17	7.05E-16	28.1196815
DNA_REPAIR	0.22437481	-0.0034042	7.33850811	6.33E-12	7.91E-11	16.3789228
GLYCOLYSIS	0.18534653	0.00473505	7.1953282	1.45E-11	1.45E-10	15.5671874
MTORC1_SIGNALING	0.26476318	-0.0001034	6.96182188	5.47E-11	4.55E-10	14.2629756
SPERMATOGENESIS	0.13705404	-0.0060736	6.84779986	1.04E-10	7.41E-10	13.6352906
MYC_TARGETS_V1	0.30548907	-0.0126652	6.73285571	1.97E-10	1.23E-09	13.008826
HYPOXIA	0.16560044	0.0061039	6.09035592	6.23E-09	3.46E-08	9.63090015
MITOTIC_SPINDLE	0.15126204	-0.0060318	5.2671278	3.77E-07	1.89E-06	5.6434051
UV_RESPONSE_UP	0.12084831	0.00347828	5.12768226	7.27E-07	3.30E-06	5.00969055
WNT_BETA_CATENIN_SIGNALING	0.12861425	-0.0004052	4.95076589	1.64E-06	6.83E-06	4.22421595
UNFOLDED_PROTEIN_RESPONSE	0.14989728	0.00489822	4.7716914	3.66E-06	1.41E-05	3.45081795
COAGULATION	-0.1177664	0.01808585	-4.0717475	6.87E-05	0.00024544	0.64814748
PI3K_AKT_MTOR_SIGNALING	0.09626966	0.00689624	3.74365221	0.00024101	0.00080338	-0.5379462

OXIDATIVE_PHOSPHORYLATION	0.15983546	0.00257858	3.70825231	0.00027465	0.00085827	-0.6608261
PANCREAS_BETA_CELLS	-0.0908402	0.00379563	-3.671425	0.00031431	0.00092445	-0.7875907
BILE_ACID_METABOLISM	-0.0851669	0.00110505	-3.5439368	0.00049747	0.00138185	-1.217932
HEME_METABOLISM	-0.0671888	0.0066789	-3.3548526	0.00096076	0.00252832	-1.8316055
CHOLESTEROL_HOMEOSTASIS	0.09404753	0.00375391	3.06572281	0.00249165	0.00622912	-2.7119347
KRAS_SIGNALING_UP	-0.0942171	0.01565082	-3.0135074	0.00293898	0.00699756	-2.863299
MYOGENESIS	-0.0770796	0.00837093	-2.7257852	0.00702189	0.01595885	-3.6546981
ALLOGRAFT_REJECTION	-0.1121135	0.00681413	-2.5527552	0.01148268	0.02475192	-4.0953148
APICAL_SURFACE	0.06964485	0.01044256	2.54043549	0.01188092	0.02475192	-4.1256613
REACTIVE_OXYGEN_SPECIES_PATHWAY	0.08625064	0.01095226	2.31909102	0.02146628	0.04293257	-4.6474128
UV_RESPONSE_DN	-0.0760233	0.01577033	-2.13201	0.03430572	0.06597254	-5.0533439
ESTROGEN_RESPONSE_LATE	0.04586458	0.00316417	2.04852074	0.04190057	0.07759365	-5.2240135
COMPLEMENT	-0.0688953	0.01452027	-1.8888201	0.06045753	0.10795988	-5.5322725
INTERFERON_GAMMA_RESPONSE	-0.0769053	0.0093865	-1.6105409	0.10895957	0.18786133	-6.011679
P53_PATHWAY	0.04184566	0.00392074	1.48662591	0.13879081	0.23131802	-6.2012956
ADIPOGENESIS	0.04480044	0.00963237	1.43334193	0.1534231	0.24745662	-6.2782763
FATTY_ACID_METABOLISM	0.0432274	0.01027545	1.30118142	0.19479071	0.30436048	-6.4573306
IL6_JAK_STAT3_SIGNALING	-0.0465006	0.01290892	-1.1488071	0.25209681	0.38196486	-6.6426508
APICAL_JUNCTION	0.03059531	0.00864547	1.11210333	0.26751554	0.38253949	-6.6838955
INFLAMMATORY_RESPONSE	-0.0434631	0.01356554	-1.1114923	0.26777764	0.38253949	-6.684571
INTERFERON_ALPHA_RESPONSE	-0.0520514	0.00513076	-1.0077681	0.31486268	0.43730928	-6.7939138
IL2_STAT5_SIGNALING	-0.0289072	0.01251047	-0.9355302	0.35071669	0.47394148	-6.8638088
TNFA_SIGNALING_VIA_NFKB	0.03045472	0.00770569	0.744868	0.45728278	0.60168787	-7.0235261
TGF_BETA_SIGNALING	-0.0269244	0.01479187	-0.6749166	0.50055896	0.64174226	-7.0730917
EPITHELIAL_MESENCHYMAL_TRANSITION	0.03122729	0.01094246	0.6319758	0.52817138	0.66021423	-7.1011105
PROTEIN_SECRETION	-0.0262293	0.01990055	-0.5966939	0.55143049	0.66146549	-7.1227598
XENOBIOTIC_METABOLISM	-0.0137783	0.01061572	-0.5854544	0.55894519	0.66146549	-7.1293963
APOPTOSIS	0.01588059	0.00974914	0.57073699	0.56886032	0.66146549	-7.1378963
KRAS_SIGNALING_DN	0.012086	0.00258651	0.48211778	0.63028377	0.71623155	-7.1845173
PEROXISOME	-0.009759	0.00261412	-0.3654055	0.71521999	0.79468888	-7.2339684
HEDGEHOG_SIGNALING	-0.0103351	0.00843458	-0.320666	0.7488201	0.79768747	-7.2493188
ANGIOGENESIS	0.01404232	0.01350775	0.31933666	0.74982623	0.79768747	-7.2497443
ESTROGEN_RESPONSE_EARLY	0.00200161	0.00758029	0.08052791	0.93590317	0.97489913	-7.2975159
ANDROGEN_RESPONSE	0.00133096	0.01443675	0.03999766	0.96813749	0.98743965	-7.2999604
NOTCH_SIGNALING	-0.0004665	0.00279287	-0.0157637	0.98743965	0.98743965	-7.3006366

GSVA enrichment HALLMARK functions between high and low-PCDscoregroup in GSE72094

Tag	logFC	AveExpr	t	P.Value	adj.P.Val	B
GLYCOLYSIS	0.21710328	-0.0059096	14.3845921	7.93E-39	3.97E-37	77.3308448
MYC_TARGETS_V2	0.35724782	-0.0181254	12.2438258	6.34E-30	1.58E-28	56.9627948
G2M_CHECKPOINT	0.30665288	-0.0173738	11.6562407	1.34E-27	2.24E-26	51.6454177
MYC_TARGETS_V1	0.2684288	-0.015591	11.5378618	3.89E-27	4.87E-26	50.590782
E2F_TARGETS	0.33545895	-0.0226865	11.4986279	5.53E-27	5.53E-26	50.2425272
MTORC1_SIGNALING	0.20777102	-0.006153	11.4602404	7.79E-27	6.49E-26	49.9024088
DNA_REPAIR	0.15891522	-0.0110652	9.89802454	5.12E-21	3.66E-20	36.6282953
MITOTIC_SPINDLE	0.13127992	-0.008993	8.60849483	1.27E-16	7.96E-16	26.624335
UNFOLDED_PROTEIN_RESPONSE	0.13411516	-0.0063133	7.0604341	6.38E-12	3.55E-11	15.9739545
OXIDATIVE_PHOSPHORYLATION	0.14000901	-0.0114242	6.25885245	9.10E-10	4.55E-09	11.1198978
SPERMATOGENESIS	0.08030376	-0.0049354	5.94343803	5.62E-09	2.56E-08	9.34380679
ESTROGEN_RESPONSE_LATE	0.06905733	-0.0022501	5.71522399	2.00E-08	8.35E-08	8.10766535
BILE_ACID_METABOLISM	-0.0829384	0.00096038	-5.5688042	4.43E-08	1.71E-07	7.33659054
ALLOGRAFT_REJECTION	-0.1437355	0.01401155	-4.8829511	1.46E-06	5.21E-06	3.9599172
HYPOXIA	0.0842771	0.00031816	4.67018078	3.99E-06	1.33E-05	2.99306521
KRAS_SIGNALING_DN	-0.0469789	0.0068902	-4.468917	9.98E-06	3.12E-05	2.11450914
HEDGEHOG_SIGNALING	-0.0913385	0.00529667	-4.429016	1.19E-05	3.50E-05	1.94453034
REACTIVE_OXYGEN_SPECIES_PATHWAY	0.09825984	-0.0043763	4.27935369	2.29E-05	6.37E-05	1.31943595
KRAS_SIGNALING_UP	-0.0830934	0.0163604	-4.2252653	2.89E-05	7.62E-05	1.09839178
IL6_JAK_STAT3_SIGNALING	-0.1053056	0.01622959	-3.8265279	0.00014847	0.00037117	-0.4505068
MYOGENESIS	-0.0695615	0.00553376	-3.5989193	0.00035527	0.00084589	-1.2702205
UV_RESPONSE_DN	-0.0675196	0.00571081	-3.572143	0.00039253	0.00089211	-1.3635402
HEME_METABOLISM	-0.0396568	0.00064675	-3.4399678	0.00063638	0.00138344	-1.8145324
CHOLESTEROL_HOMEOSTASIS	0.06201232	-0.0013304	3.31714997	0.00098365	0.00199013	-2.2191329
IL2_STAT5_SIGNALING	-0.0657022	0.01273005	-3.312192	0.00100082	0.00199013	-2.2351724
INFLAMMATORY_RESPONSE	-0.0857084	0.0151908	-3.3025889	0.00103487	0.00199013	-2.2661739
UV_RESPONSE_UP	0.04417687	0.00029316	3.26428057	0.00118169	0.00218832	-2.3889915
PROTEIN_SECRETION	0.05112901	-0.0031184	3.22326058	0.00136016	0.00242886	-2.5189877
PI3K_AKT_MTOR_SIGNALING	0.0404931	0.00291661	2.88598284	0.00409087	0.00705322	-3.528099
COMPLEMENT	-0.0589269	0.0134598	-2.6770296	0.00770107	0.01283512	-4.0993966
APICAL_SURFACE	-0.0451841	0.00774715	-2.4779077	0.01358394	0.02190958	-4.6050983
FATTY_ACID_METABOLISM	0.04144669	-0.0061453	2.29511976	0.02218869	0.03438816	-5.0358288
EPITHELIAL_MESENCHYMAL_TRANSITION	0.07455196	0.00378558	2.28642747	0.02269618	0.03438816	-5.0555098

ANGIOGENESIS	0.05691404	0.00874816	2.17375336	0.03024784	0.04448211	-5.3040179
TGF_BETA_SIGNALING	-0.0394669	0.00478329	-1.8165594	0.06995427	0.09993467	-6.0103428
COAGULATION	-0.0310036	0.01303114	-1.651746	0.09928865	0.1379009	-6.2942443
INTERFERON_GAMMA_RESPONSE	-0.0480124	0.01126827	-1.5313757	0.12638401	0.1707892	-6.4847434
PEROXISOME	-0.021117	-0.0018099	-1.4895197	0.13705582	0.1803366	-6.5476446
ESTROGEN_RESPONSE_EARLY	0.02020657	0.00047156	1.46365769	0.14399039	0.18460306	-6.5856476
APICAL_JUNCTION	0.02216599	0.00865736	1.15499329	0.24871026	0.31088783	-6.988233
ADIPOGENESIS	0.01307159	-0.0005836	0.83805422	0.40244817	0.49079045	-7.3033605
TNFA_SIGNALING_VIA_NFKB	-0.0214662	0.00237599	-0.7707944	0.44123602	0.52528098	-7.357389
PANCREAS_BETA_CELLS	-0.0124738	0.00066424	-0.7340097	0.46332769	0.53875313	-7.3850316
P53_PATHWAY	-0.0109465	0.00169943	-0.7163995	0.47411881	0.53877138	-7.3977879
NOTCH_SIGNALING	-0.0083827	0.00302563	-0.4278185	0.66898935	0.7433215	-7.562764
ANDROGEN_RESPONSE	-0.0045704	0.00171808	-0.3083337	0.75797209	0.80754753	-7.6067311
WNT_BETA_CATENIN_SIGNALING	-0.0054064	0.00235797	-0.3068576	0.75909468	0.80754753	-7.607185
INTERFERON_ALPHA_RESPONSE	0.00928355	0.01103864	0.26370867	0.79212594	0.82513119	-7.6194928
XENOBIOTIC_METABOLISM	-0.0020464	0.0031522	-0.1386375	0.88979905	0.90795821	-7.644652
APOPTOSIS	0.00119311	0.00997577	0.06645864	0.9470424	0.9470424	-7.6520538

GSVA enrichment HALLMARK functions between high and low-PCDscoregroup in GSE31210

Tag	logFC	AveExpr	t	P.Value	adj.P.Val	B
GLYCOLYSIS	0.38301869	-0.0045916	17.8020909	1.88E-46	9.40E-45	94.8974394
MTORC1_SIGNALING	0.42344306	-0.0113756	17.6310654	7.27E-46	1.82E-44	93.5474998
UNFOLDED_PROTEIN_RESPONSE	0.34561066	-0.0073474	15.6996736	3.39E-39	5.65E-38	78.2334782
MYC_TARGETS_V1	0.42612733	-0.0212983	14.9228096	1.65E-36	2.06E-35	72.0662094
E2F_TARGETS	0.47213655	-0.0312316	14.5898449	2.32E-35	2.32E-34	69.4277178
G2M_CHECKPOINT	0.39655946	-0.0246535	13.2063169	1.30E-30	1.08E-29	58.5345272
MYC_TARGETS_V2	0.39270014	-0.0242504	12.9347971	1.09E-29	7.80E-29	56.4166747
DNA_REPAIR	0.31020966	-0.009591	12.8536326	2.06E-29	1.29E-28	55.7852668
PROTEIN_SECRETION	0.29239919	-0.0016918	12.0273194	1.25E-26	6.92E-26	49.4088469
OXIDATIVE_PHOSPHORYLATION	0.33493972	-0.0122518	10.4670109	1.60E-21	7.99E-21	37.7162661
PI3K_AKT_MTOR_SIGNALING	0.19667989	-0.0029553	9.75058898	2.90E-19	1.32E-18	32.5554611
REACTIVE_OXYGEN_SPECIES_PATHWAY	0.20242751	-0.0036705	8.42099907	2.86E-15	1.19E-14	23.4464963
ANDROGEN_RESPONSE	0.14535941	-0.0028583	7.97689748	5.28E-14	2.03E-13	20.5665689
UV_RESPONSE_UP	0.15540818	-0.0011948	7.66841791	3.79E-13	1.35E-12	18.62101
HYPOXIA	0.18402293	-0.0014396	7.64554161	4.38E-13	1.46E-12	18.47861
MITOTIC_SPINDLE	0.14216575	-0.0077348	7.59127946	6.17E-13	1.93E-12	18.1419017
MYOGENESIS	-0.1515978	0.00011706	-6.8971616	4.26E-11	1.25E-10	13.9725988
INTERFERON_ALPHA_RESPONSE	0.27746548	0.00092603	6.56941935	2.88E-10	7.99E-10	12.0983408
UV_RESPONSE_DN	-0.1280173	0.00528306	-5.672464	3.85E-08	1.01E-07	7.31075993
INTERFERON_GAMMA_RESPONSE	0.20768953	0.00866199	5.58260145	6.12E-08	1.53E-07	6.8604878
APOPTOSIS	0.1158059	0.00641402	5.41669029	1.42E-07	3.37E-07	6.04396056
ADIPOGENESIS	0.13161947	0.0019125	5.24065913	3.39E-07	7.69E-07	5.19900919
FATTY_ACID_METABOLISM	0.12832255	-0.0023642	5.22506571	3.65E-07	7.94E-07	5.1252379
ESTROGEN_RESPONSE_LATE	0.08322242	3.23E-05	4.80282386	2.69E-06	5.40E-06	3.19596218
SPERMATOGENESIS	0.07049199	-0.0017604	4.80195319	2.70E-06	5.40E-06	3.19212245
BILE_ACID_METABOLISM	-0.0802853	0.00543095	-4.5548726	8.17E-06	1.57E-05	2.12609756
IL6_JAK_STAT3_SIGNALING	0.13524161	0.0026333	4.37084088	1.81E-05	3.35E-05	1.36316316
COMPLEMENT	0.11520645	0.00507262	4.26182781	2.87E-05	5.12E-05	0.92397897
ALLOGRAFT_REJECTION	0.13121237	0.00329676	3.84941746	0.00015017	0.00025891	-0.64966
CHOLESTEROL_HOMEOSTASIS	0.09831607	-0.0040412	3.54883425	0.00046148	0.00076914	-1.7067581
INFLAMMATORY_RESPONSE	0.10111057	0.00590991	3.36890676	0.00087296	0.00137376	-2.3023258
NOTCH_SIGNALING	-0.0843372	0.00600555	-3.3668512	0.0008792	0.00137376	-2.3089667
XENOBIOTIC_METABOLISM	0.06042633	0.00276771	3.34769422	0.00093947	0.00142343	-2.3706785
EPITHELIAL_MESENCHYMAL_TRANSITION	0.1214046	0.00095902	3.19789579	0.00156152	0.00229636	-2.8420887
PEROXISOME	0.06462652	-0.00096	3.12804665	0.00196663	0.00280947	-3.0551028
KRAS_SIGNALING_DN	-0.0370435	0.00315107	-2.9773046	0.00319137	0.00443246	-3.4999392
WNT_BETA_CATENIN_SIGNALING	-0.0599334	0.00279638	-2.8038671	0.00544264	0.00735491	-3.9863665
IL2_STAT5_SIGNALING	0.049473	0.00298012	2.17498421	0.03056194	0.04021308	-5.5175639
P53_PATHWAY	0.0384629	-0.0009502	2.12658799	0.03442681	0.04413693	-5.6200318
PANCREAS_BETA_CELLS	0.04134868	-0.0019837	1.88773119	0.0602116	0.0752645	-6.0930626
TGF_BETA_SIGNALING	-0.0567822	-0.0003828	-1.8110654	0.07132201	0.08697806	-6.2332982
ANGIOGENESIS	0.05254435	0.00296081	1.57357599	0.11684075	0.13909613	-6.6316631
COAGULATION	0.02866824	0.00410528	1.45452451	0.14704597	0.17098368	-6.8107335
HEDGEHOG_SIGNALING	-0.0287901	0.00047397	-1.184954	0.23715247	0.26949144	-7.1649164
HEME_METABOLISM	0.01237505	0.00255379	0.86834661	0.38603123	0.42892359	-7.4893903
KRAS_SIGNALING_UP	0.01868133	0.00612333	0.81331536	0.41680542	0.45304936	-7.5356489
ESTROGEN_RESPONSE_EARLY	-0.0147261	0.00113379	-0.7953492	0.42715929	0.45442478	-7.550099
TNFA_SIGNALING_VIA_NFKB	0.0104004	-0.0028786	0.27954801	0.78005372	0.81255596	-7.8276235
APICAL_SURFACE	0.00447475	0.00846065	0.19531419	0.84530445	0.86255556	-7.8476691
APICAL_JUNCTION	0.00213137	0.00190307	0.10581898	0.91581021	0.91581021	-7.8611768

Supplementary Table 6 The correlation between the IC50 of various drugs and PCDI

	CHEK2	KRT18	GAPDH	MMP1	CHRNA5	TMPRSS4	ITGB4	CD79A	CTLA4	RRM2	PCDI
Camptothecin_1003	-0.0277197	0.21147061	0.03807721	-0.0599118	-0.0078515	0.07258063	0.16814124	-0.3263178	-0.4105553	-0.016624	0.24060552
Cisplatin_1005	-0.3014577	0.13877372	-0.1137259	-0.2116566	-0.1376721	0.12009934	0.23027804	-0.3820834	-0.4682946	-0.269967	0.12822277
Cytarabine_1006	-0.1228067	0.10009897	0.00613326	-0.057495	-0.1727008	0.06298496	0.18530429	-0.2381881	-0.3043414	-0.0378578	0.17869708
Docetaxel_1007	-0.3708936	-0.1575163	-0.4047335	-0.2409157	-0.3019893	0.06475126	-0.0297939	-0.129629	-0.0692159	-0.4133552	-0.2595271
Gefitinib_1010	-0.3289532	-0.1808259	-0.1973529	-0.0830007	-0.1552752	-0.0394872	-0.2689281	-0.0531831	-0.073148	-0.2159908	-0.1601608
Navitoclax_1011	-0.4257151	-0.1465191	-0.2062069	-0.0960176	-0.2226856	-0.1362883	-0.064148	-0.3846643	-0.5037999	-0.2535308	0.15576992
Vorinostat_1012	-0.2151488	0.12500688	0.10377596	0.0451534	-0.1010756	0.15473963	0.268215	-0.3295218	-0.3204994	0.01565773	0.34237699
Nilotinib_1013	-0.3748422	0.04080437	-0.1341602	-0.0286712	-0.1904229	0.06870403	0.20066015	-0.3492303	-0.4195549	-0.1444665	0.27588086
Olaparib_1017	-0.0583499	0.3022778	0.19137643	-0.040632	-0.0195933	0.19119625	0.28809323	-0.3649252	-0.3294522	0.10340985	0.37059867
Axitinib_1021	-0.4124311	-0.2053416	-0.2635549	-0.1894873	-0.1171377	-0.2675695	-0.2133281	-0.4782055	-0.5474287	-0.175795	0.22124766
AZD7762_1022	-0.2952852	0.01522821	-0.2876414	-0.2574199	-0.0681878	0.13087336	-0.0297172	-0.2677386	-0.4035883	-0.4114073	-0.1409257
SB216763_1025	0.1272441	0.21934127	0.18336618	-0.1434112	0.26705817	-0.0216762	-0.0612562	-0.5516313	-0.6256218	0.21956315	0.47420601
KU-55933_1030	0.04758155	0.16856047	-0.0114773	-0.3226634	0.16492299	-0.0319197	-0.0697398	-0.490972	-0.4588715	-0.0390492	0.18280736
Afatinib_1032	-0.3429652	-0.2830414	-0.2761091	-0.1255792	-0.04475	-0.2860722	-0.4493504	-0.0952129	-0.1055171	-0.1657597	-0.1252732
PLX-4720_1036	-0.3005547	-0.1020228	-0.2383449	-0.278177	-0.0438952	-0.1454581	-0.2371357	-0.4401521	-0.4658228	-0.1282739	0.11424827
NU7441_1038	0.14341335	0.31061004	0.19084615	-0.1973803	0.21534308	0.11879603	0.08195872	-0.4413436	-0.3608888	0.15472449	0.33455333
Doramapimod_1042	0.16636933	0.34506768	0.43789079	0.11937237	0.22479945	-0.0061706	0.22607524	-0.4529194	-0.3373685	0.46690222	0.70087288
Nutlin-3a (-)_1047	0.25005362	0.27097474	0.30796818	-0.026692	0.10258895	0.09129576	0.08238341	-0.3084728	-0.1616701	0.42323303	0.42734069
PD173074_1049	-0.337604	-0.0159348	-0.1080423	-0.1352999	-0.1620865	-0.0441806	0.00139367	-0.4470499	-0.4501916	-0.1184216	0.24544349
ZM447439_1050	-0.3771918	-0.1798519	-0.2466623	-0.2329426	-0.052997	-0.2551585	-0.2773652	-0.4695758	-0.5535211	-0.2223149	0.13588029
RO-3306_1052	-0.1610234	0.11774988	0.00537315	-0.1282227	-0.0139345	-0.0371138	-0.0062172	-0.3802036	-0.3955929	-0.0391429	0.28103798
MK-2206_1053	-0.3212011	-0.1600476	-0.1885728	-0.0894827	-0.1220665	-0.2191229	-0.1632907	-0.4647321	-0.3192614	0.02645143	0.29819633
Palbociclib_1054	-0.1970075	-0.1642713	-0.1983832	-0.116156	-0.1318079	-0.178303	-0.1206853	-0.3537631	-0.3271859	0.04634263	0.22025061
Dactolisib_1057	-0.1698423	-0.1361408	-0.1741217	-0.1143722	-0.1247409	-0.0469309	-0.0853758	-0.3138296	-0.208913	0.03556505	0.18278772
AZD8055_1059	-0.2359608	-0.096765	-0.2274847	-0.194474	0.01189759	-0.1038273	-0.1155196	-0.5779018	-0.6053584	-0.095145	0.3031285
Dasatinib_1079	-0.2144958	-0.1684553	-0.3462612	-0.3974917	0.07893424	-0.0665365	-0.3699857	-0.3327124	-0.4172416	-0.317197	-0.1978192
Paclitaxel_1080	-0.5865981	-0.2939354	-0.487601	-0.2211782	-0.3330026	-0.0334875	-0.11098	-0.2245567	-0.2420883	-0.4803678	-0.1858105
Rapamycin_1084	-0.5523774	-0.287413	-0.4050557	-0.1509988	-0.284199	-0.0835913	-0.0835924	-0.3847978	-0.380851	-0.2764471	0.10321643
Sorafenib_1085	-0.3753892	-0.0563088	-0.1256252	0.02389747	-0.2689114	-0.016138	0.12623486	-0.3191844	-0.2624195	-0.1026959	0.24606538
BI-2536_1086	0.02687951	0.26796386	0.17383359	0.06518288	-0.0854916	0.32565163	0.37086332	0.39948565	0.45562019	-0.1110485	-0.2339054
Irinotecan_1088	-0.0741568	0.19835609	0.06749791	-0.0517816	-0.0248552	0.03566093	0.17878663	-0.3692143	-0.3926381	0.03431427	0.31180042
Oxaliplatin_1089	-0.0873808	0.12536836	0.03973475	-0.0086275	-0.0766268	-0.0007775	0.1724065	-0.3503792	-0.3267436	0.06302105	0.30109968
BMS-536924_1091	-0.1208741	-0.2878781	-0.3180231	-0.2703974	-0.1617891	-0.1297059	-0.3748197	-0.2382168	-0.1241596	-0.1031754	-0.1310854
GSK1904529A_1093	-0.4388571	-0.0567293	-0.1291898	0.05637147	-0.2923928	0.07866465	0.1963655	-0.2172034	-0.1790327	-0.188665	0.14296416
PF-4708671_1129	-0.3190767	-0.2441043	-0.2427395	-0.1631264	-0.0424856	-0.3073561	-0.2901456	-0.532464	-0.5808514	-0.0871819	0.25308408
PRIMA-1MET_1131	0.07838335	0.32802524	0.28609589	-0.0009399	0.29930523	0.18262504	0.12340439	-0.3947586	-0.5109173	0.22137079	0.47778522
Erlotinib_1168	0.13718414	0.21749063	0.13188175	0.0617878	0.06969945	0.24349619	0.09172509	0.14297505	0.18009192	0.02450599	-0.0917484
Niraparib_1177	0.07140338	0.32214259	0.28671798	0.05338558	0.0721365	0.15085776	0.25082335	-0.3409469	-0.3504848	0.29158163	0.49028175
MK-1775_1179	-0.5717767	-0.3397528	-0.5238106	-0.234828	-0.3713942	-0.0722608	-0.201241	-0.2551298	-0.397283	-0.5801197	-0.2440999
Dinaciclib_1180	-0.3549613	-0.096433	-0.1888302	-0.2037468	-0.1163941	-0.028828	-0.098145	-0.4058694	-0.3709944	-0.1739125	0.10378723
Gemcitabine_1190	-0.0636418	0.14825993	-0.0367003	-0.1463507	-0.0080621	0.05956283	0.0740288	-0.3374989	-0.4628606	-0.1149328	0.14338973
GSK269962A_1192	-0.2580109	-0.1337514	-0.1202522	-0.1561709	-0.0216621	-0.2059719	-0.1649784	-0.5639795	-0.5149784	0.03029698	0.36765154
SB505124_1194	0.53059374	0.43695547	0.58167508	0.29573509	0.16547041	0.23191779	0.42373742	0.36483873	0.54218374	0.53380435	0.19816972
Tamoxifen_1199	-0.1206625	0.15932373	0.0234707	0.06235868	-0.1544163	0.17178167	0.30137564	-0.2178422	-0.0746031	0.01490149	0.21035645
Fulvestrant_1200	-0.3269822	0.13000195	0.02569402	-0.0210256	-0.1094127	0.10508132	0.21507545	-0.37972	-0.3970621	-0.076634	0.31118109
EPZ004777_1237	-0.3106507	0.12711819	-0.0124196	-0.0914726	-0.0739911	0.06794677	0.10449374	-0.4966123	-0.5132459	-0.0950251	0.31315418
YK-4-279_1239	-0.4559584	-0.2339708	-0.4410561	-0.2604067	-0.2730775	-0.0587928	-0.172119	-0.3426605	-0.4075887	-0.4046008	-0.0959561
Daporinad_1248	-0.1413959	0.19708194	0.17328344	0.03326987	-0.0794574	0.05615669	0.23418284	-0.1388559	-0.0969175	-0.0020907	0.20625789
BMS-345541_1249	-0.5177258	-0.4059038	-0.5011478	-0.1612714	-0.3674142	-0.1713522	-0.2968315	-0.2389862	-0.3188027	-0.3826901	-0.1171059
Talazoparib_1259	-0.1087286	0.14497762	0.06487503	-0.0472603	-0.1449804	0.06196493	0.20673609	-0.3380487	-0.3275079	0.06095955	0.33029617
XAV939_1268	-0.1918474	0.01674988	-0.107352	-0.2033078	-0.0322733	0.09787392	-0.0906039	-0.4001275	-0.4816931	-0.1430299	0.10778211
Trametinib_1372	0.36935812	-0.0174672	0.01663268	-0.1961666	0.22031076	-0.0944165	-0.3832133	0.06686467	0.05819882	0.20708371	-0.1358696
Dabrafenib_1373	-0.2070888	-0.0474304	-0.153856	-0.1865404	-0.1729331	-0.0381284	0.01187443	-0.4211633	-0.3531226	-0.1194903	0.14867022
Temozolomide_1375	-0.2706478	0.06356931	-0.0908165	-0.1154833	-0.1231508	0.0786245	0.08610547	-0.4116314	-0.4137463	-0.2115924	0.1361973
AZD5438_1401	-0.2069344	0.01078886	-0.0976212	-0.2150812	-0.0894353	-0.0055218	0.02451142	-0.4162667	-0.3627673	-0.084347	0.17008525
IAP_5620_1428	-0.096152	0.26754001	0.11545755	0.03824162	0.0027803	0.31288597	0.40505164	-0.1210279	-0.1087557	-0.065398	0.13685253
AZD2014_1441	-0.015587	-0.0904356	-0.1044965	-0.2313582	0.0121927	-0.1014063	-0.301142	-0.3736152	-0.2000355	0.13228026	0.15941876
AZD1208_1449	-0.1410731	0.23870015	0.15862625	0.02180018	-0.0941443	0.12544793	0.23961292	-0.3849627	-0.3757237	0.05454507	0.40379169
AZD1332_1463	-0.1601289	-0.0928128	-0.1372288	-0.288812	0.03586774	-0.1200603	-0.2953631	-0.40291	-0.39671	-0.0209198	0.12859419
Ruxolitinib_1507	-0.2932896	0.03551429	-0.0963706	-0.1102966	-0.0670396	0.04770246	0.00856058	-0.3905573	-0.5987498	-0.1866739	0.20297018
Linsitinib_1510	-0.3958807	-0.2270882	-0.2022813	-0.0180784	-0.3106215	-0.084951	-0.0761454	-0.3158402	-0.2103956	-0.1044472	0.13110564
Epirubicin_1511	0.03152348	0.23767845	0.15137505	-0.0602686	0.08279156	0.16422856	0.14572271	-0.317931	-0.3158861	0.11308605	0.29470738
Cyclophosphamide_1	-0.2896536	0.1546426	0.02401092	-0.0495687	-0.0872785	0.12609269	0.22896384	-0.3999638	-0.3943467	-0.067247	0.30947982
Sapitinib_1549	-0.1180685	-0.2013092	-0.1267395	-0.0650085	0.07940717	-0.1940239	-0.4488041	0.02970899	0.10300694	-0.0259975	-0.1956667
Uprosertib_1553	-0.1927794	-0.0657224	-0.1065692	-0.0707687	0.01522752	-0.1726248	-0.2129491	-0.3425369	-0.216227	0.12174419	0.26917097
Lapatinib_1558	-0.5070329	-0.3511463	-0.40912	-0.127796	-0.3072722	-0.1272407	-0.2315658	-0.1495692	-0.1285243	-0.3437011	-0.1781544
EPZ5676_1563	-0.2434989	0.18184491	0.01304947	-0.047625	-0.0830963	0.14791334	0.17471008	-0.3785253	-0.4425576	-0.1154802	0.26073149
SCH772984_1564	0.27637337	-0.015508	-0.0308547	-0.1916494	0.11904367	-0.0468794	-0.2982433	0.11089062	0.09352661	0.0942668	-0.2193997
IWP-2_1576	-0.3923308	-0.0312623	-0.2008842	-0.1212206	-0.1980808	0.05738098	0.03040269	-0.4090452	-0.5249751	-0.2657211	0.12557762
Leflunomide_1578	-0.2158472	0.0455806	0.00198831	0.01470206	-0.042586	0.03230109	0.0891186	-0.368572	-0.3802388	0.00513634	0.30008921
Entinostat_1593	-0.0587053	0.28466076	0.06954789	-0.0941603	-0.0110834	0.24897073	0.27478867	-0.3259402	-0.4036794	-0.0646736	0.207324
LGK974_1598	-0.3532382	-0.1063772	-0.1409596	-0.0225384	-0.2917569	-0.1087744	0.09198158	-0.3265975	-0.2686678	-0.1726741	0.1555757
WZ4003_1614	-0.0788913	0.04043864	-0.0771689	-0.223275	0.09929975	0.01858221	-0.112886	-0.5317966	-0.4968433	0.07062608	0.27381809
CZC24832_1615	-0.2598519	0.08642416	-0.108332	-0.2005296	0.03069893	0.03880825	0.01404956	-0.5533052	-0.6467746	-0.1426373	0.2850782
GSK2606414_1618	-0.2694532	-0.147794	-0.2544125	-0.2236638	-0.0704999	-0.1340585	-0.2569616	-0.4049342	-0.4623555	-0.1361797	0.11605231
PFI3_1620	-0.2860129	0.13527323	-0.0472008	-0.139232	-0.0031626	0.12391357	0.09820985	-0.4717383	-0.5726917	-0.1525145	0.26834813
PCI-34051_1621	-0.0441906	0.2840442	0.25067046	-0.0397671	0.07084381	0.20260312	0.23004154	-0.4701704	-0.4545255	0.16664382	0.48990161
Wnt-C59_1622	-0.4411029	-0.1108899	-0.1528466	-0.0027301	-0.2429532	-0.0208614	0.09405102	-0.3990509	-0.4057715	-0.1481203	0.26498083
I-BET-762_1624	0.04184103	0.21737686	-0.0663597	-0.1940736	0.11905597	0.23066666	0.05034031	-0.3533845	-0.360626	-0.0021065	0.15666618
RVX-208_1625	-0.2549406	0.07465195	-0.1961042	-0.2363205	-0.1056708	0.12819464	0.07582823	-0.4797433	-0.539563	-0.2317798	0.12995805
OTX015_1626	0.12250147	0.22317328	0.03499389	-0.1653971	0.18984527	0.29406001	0.00971555	-0.2370508	-0.2089697	0.10018816	0.10997602
GSK343_1627	-0.1426799	0.24214161	0.07642123	-0.1041296	0.05568455	0.15514973	0.1307202	-0.4870491	-0.5518751	0.00622529	0.36439494
ML323_1629	-0.5311818	-0.249094	-0.2990649	-0.011815	-0.4174129	-0.1076729	0.08911671	-0.278501	-0.2396656	-0.2688879	0.10201833
PRT062607_1631	-0.1104652	-0.0027255	-0.0700459	-0.1764975	0.08161356	-0.0985877	-0.2086721	-0.5598318	-0.5758984	0.06454228	0.30343783
Ribociclib_1632	-0.2171699	-0.1321718	-0.1884271	-0.1793387	-0.0064759	-0.2320682	-0.1822425	-0.6185128	-0.6890525	-0.0571473	0.33367817
AGI-6780_1634	-0.235078	0.16863453	0.05639194	0.01414028	-0.1396567	0.12229582	0.30031639	-0.346812	-0.3089905	-0.0458333	0.31752123
Picolinici-acid_1635	-0.1308239	0.2090943	0.05996347	-0.0908542	-0.0272543	0.11479507	0.16795767	-0.4669043	-0.4940557	0.00902607	0.34874179
AZD5153_1706	0.06632862	0.18422346	0.10157223	-0.0632745	0.10839696	0.24149765	0.04936232	-0.328715	-0.2623527	0.16835513	0.26492775
CDK9_5576_1708	-0.1972408	-0.0438514	-0.0568382	-0.1692856	-0.1334924	-0.0536032	-0.0509878	-0.377217	-0.3112712	0.01387188	0.20802158
CDK9_5038_1709	-0.2420695	0.02851594	0.00099661	-0.1277094	-0.0729038	0.03190781	0.04649165	-0.4129337	-0.2933852	0.02044266	0.2441226
ERK_2440_1713	0.33932715	0.11230008	0.05238598	-0.2223479	0.32850078	-0.0250834	-0.2880763	-0.2319032	-0.324108	0.2600894	0.15578206
ERK_6604_1714	0.26969614	-0.034111	-0.060468	-0.2622408	0.11660363	-0.0111225	-0.2830069	0.0635097	0.04456072	0.07080149	-0.2410609
IRAK4_4710_1716	-0.1266907	0.15501506	0.06617676	-0.01815	-0.1152704	0.12839223	0.21029922	-0.2768021	-0.2200904	-0.0065993	0.23488514
JAK1_8709_1718	0.03981774	0.15445029	0.18446268	0.03475662	0.01983464	0.10797014	0.13878449	-0.3542685	-0.2620618	0.28365751	0.43184301
AZD5991_1720	-0.2960936	0.14632767	0.01806303	0.06336004	-0.1531729	0.19003254	0.31385653	-0.2789606	-0.2417609	-0.0731655	0.29110867
PAK_5339_1730	-0.3908868	-0.1202606	-0.1986467	-0.1224787	-0.1752827	-0.0174472	-0.0833041	-0.474164	-0.4307337	-0.1522828	0.16994913

TAF1_5496_1732	-0.1000162	0.15462521	0.22243482	0.16186572	-0.2268938	0.02773541	0.37645887	-0.1359319	0.03413446	0.13938343	0.35080849
JAK_8517_1739	0.01070863	0.16190048	0.0644485	-0.2159946	0.11931502	0.05865134	-0.1253858	-0.3219625	-0.3058083	0.0609285	0.14581813
AZD4547_1786	-0.2285584	0.05935227	-0.0526386	-0.0409755	-0.2954323	0.08800339	0.24255436	-0.2669188	-0.0874726	-0.0717677	0.1585501
Zoledronate_1802	-0.2447564	0.09769494	0.00958223	-0.0441162	-0.082386	0.06816259	0.09785971	-0.4100961	-0.4497548	-0.0158908	0.35049527
Oxaliplatin_1806	0.02195937	0.21149385	0.17682132	0.05388806	0.02710091	0.06738403	0.16628365	-0.3260506	-0.2703958	0.22988837	0.38090181
Carmustine_1807	-0.3145705	0.06763343	-0.0303745	-0.0008047	-0.190296	0.11550581	0.15596117	-0.2644103	-0.3533693	-0.1427949	0.17926225
Topotecan_1808	-0.0187625	0.2248393	0.08072274	-0.0307688	0.04259046	0.0832327	0.16440652	-0.3441662	-0.4799709	0.01348155	0.28545711
Teniposide_1809	-0.1093864	0.15113512	-0.0407801	-0.0954119	-0.0262152	0.11031333	0.13049086	-0.4225848	-0.5524853	-0.1078454	0.23862044
Mitoxantrone_1810	0.23223636	0.42326677	0.27463525	0.03582853	0.15306198	0.23475719	0.26463574	-0.2656711	-0.3223069	0.21854716	0.3440166
Dactinomycin_1811	-0.113034	0.05491626	-0.0853975	-0.0735445	-0.048769	0.04735438	0.00262288	-0.2357633	-0.3155931	-0.0304751	0.1172423
Fludarabine_1813	-0.0648572	0.16869225	-0.0537378	-0.2113234	0.10977897	0.11114636	0.02811607	-0.3911301	-0.5715646	-0.0571196	0.20277515
Nelarabine_1814	-0.1881396	0.15238827	0.05413296	-0.0810922	-0.0319125	0.08307957	0.0667574	-0.3856965	-0.4897917	-0.066936	0.26549064
Fulvestrant_1816	-0.5091264	-0.1787651	-0.2318575	0.01691778	-0.3580782	-0.0140457	0.09779733	-0.274222	-0.3199798	-0.2770848	0.09674767
Docetaxel_1819	-0.3727593	-0.1992663	-0.3946443	-0.2619882	-0.252197	0.07493687	-0.1092043	-0.2655111	-0.2324171	-0.4035652	-0.2006404
Dihydrorotenone_182	-0.3295266	-0.1049405	-0.0274829	0.11981172	-0.358633	-0.0809313	0.23524192	-0.1130666	0.03589766	-0.0515694	0.17277058
Gallibiscoquinazole_1	-0.4895026	-0.1111952	-0.2307576	-0.0762523	-0.299572	-0.0345167	0.07909262	-0.3594092	-0.4245243	-0.3067887	0.13830101
Elephantin_1835	-0.0905699	0.19845092	0.19212407	0.0232257	0.15610568	0.14726844	0.11714022	-0.3480886	-0.3301177	0.14265423	0.37469661
Sinularin_1838	-0.26438	0.03805758	0.10311372	0.12872996	-0.105918	0.02535527	0.0838366	-0.1521811	-0.170156	0.04619639	0.27736149
Sabutoclax_1849	-0.2150834	0.17833773	0.05534815	0.04546928	-0.0457239	0.17874339	0.23055983	-0.3421915	-0.3488243	-0.0167976	0.30898012
LY2109761_1852	-0.1303236	0.15987145	0.09205695	-0.029635	-0.0503716	0.0125987	0.07329922	-0.3601542	-0.3898774	0.03253172	0.32721192
OF-1_1853	-0.1038562	0.15619797	0.22245749	0.18633178	-0.2210082	0.06963265	0.43403566	-0.1099522	0.04209485	0.16471099	0.37536142
MN-64_1854	-0.3540218	-0.0007157	-0.1125968	-0.1141623	-0.0786537	0.04106496	-0.0537685	-0.2951629	-0.3871353	-0.1794585	0.13209998
KRAS (G12C) Inhibitor	-0.3921341	-0.0476327	-0.0746157	0.00891529	-0.229198	-0.0202181	0.14579133	-0.2741592	-0.2581254	-0.1024708	0.22505463
Venetoclax_1909	-0.1463323	0.23272709	0.07811638	-0.0908839	-0.037257	0.12836886	0.26244857	-0.4477299	-0.4148352	-0.0144763	0.35444277
ABT737_1910	-0.1046448	0.27900187	0.17821029	-0.004434	0.01003696	0.15550475	0.25810578	-0.3335226	-0.3710959	-0.0015562	0.32002001
Dactinomycin_1911	-0.0047497	0.15798101	0.04957316	-0.0505247	0.00888228	0.15063101	0.11284346	-0.1743181	-0.0669797	0.07702809	0.12986414
Afuresertib_1912	-0.2954228	-0.0657279	-0.118413	-0.093794	-0.0939464	-0.2084502	-0.1009493	-0.3578599	-0.192318	0.0509424	0.25189532
AGI-5198_1913	-0.1939331	0.21054485	0.02338724	-0.0443822	-0.0807156	0.16245009	0.28062164	-0.3870318	-0.3740703	-0.0637075	0.2924694
AZD5363_1916	-0.4131431	-0.2015161	-0.3068913	-0.2015806	-0.1452874	-0.219226	-0.2163318	-0.4034378	-0.3107998	-0.1253622	0.11793163
AZD6738_1917	-0.4835789	-0.2831137	-0.5067418	-0.3091126	-0.3247267	-0.0627217	-0.2029248	-0.2569479	-0.3983306	-0.5159192	-0.2349452
AZD8186_1918	-0.122481	-0.105849	-0.2009776	-0.2467904	0.09788827	-0.1983322	-0.3590327	-0.437167	-0.4002473	-0.0030543	0.14831213
Osimertinib_1919	-0.4461068	-0.3757618	-0.3538791	-0.1226982	-0.1914873	-0.2380922	-0.3955924	-0.1436178	-0.2023087	-0.2449139	-0.0989127
Ipatasertib_1924	-0.4772454	-0.2564592	-0.3328474	-0.1468562	-0.2130676	-0.289271	-0.210748	-0.3464276	-0.2669697	-0.1496433	0.10934959
GNE-317_1926	-0.1015389	-0.1450002	-0.1432881	-0.1581862	-0.059358	-0.145334	-0.1825915	-0.4326091	-0.2416558	0.12300103	0.23637668
GSK2578215A_1927	-0.4424645	0.05003009	-0.1304864	-0.0679639	-0.2049693	0.14304293	0.25083626	-0.422361	-0.4541744	-0.2201869	0.24679261
I-BRD9_1928	-0.3972668	-0.0859848	-0.2346467	-0.1028387	-0.1894624	-0.0999901	-0.0321377	-0.440372	-0.4584602	-0.1560569	0.23525456
MIRA-1_1931	-0.4076419	0.03840341	-0.0542266	-0.036135	-0.1689547	0.08489503	0.1649025	-0.4348361	-0.487401	-0.1525971	0.28479529
NVP-ADW742_1932	-0.2449702	-0.1737265	-0.2259945	-0.1424614	-0.2340641	-0.1062967	-0.1659484	-0.3481325	-0.2019219	-0.0595204	0.10253227
P22077_1933	-0.5293775	-0.1171051	-0.2314114	0.02058112	-0.2919125	0.06611555	0.19059591	-0.2669502	-0.3213646	-0.2880112	0.15350506
Savolitinib_1936	-0.1225208	0.07672051	-0.1750437	-0.1809415	-0.1538578	0.1950356	0.12273771	-0.1403369	-0.2178893	-0.3001705	-0.1058341
UMI-77_1939	-0.3892387	0.00379134	-0.1343877	-0.1130936	-0.1632633	0.23730745	0.18137652	-0.160289	-0.1909129	-0.3899024	-0.1167299
WIKI4_1940	-0.4269364	-0.2746204	-0.3661981	-0.207301	-0.1746506	0.06062771	-0.2593204	-0.1851951	-0.35537	-0.4466158	-0.2337981
Sepantronium bromic	-0.2541227	-0.0917521	-0.1119212	0.04414079	-0.0607132	0.22704765	0.14674994	0.10902143	0.10208534	-0.2947576	-0.2037709
BIBR-1532_2043	-0.1369043	0.30123809	0.20618896	0.05154205	-0.0325469	0.22601567	0.33669148	-0.3080938	-0.2430575	0.09918364	0.37762927
Pyridostatin_2044	-0.3935225	0.0762573	-0.0840074	-0.0640067	-0.15797	0.07272089	0.13480458	-0.3203417	-0.2982233	-0.1368864	0.19646755
AMG-319_2045	-0.1926582	0.23173136	0.00144634	-0.1209715	0.09538418	0.09365166	0.11041949	-0.5104454	-0.623113	-0.0829403	0.32082896
VX-11e_2096	-0.065264	-0.2580529	-0.3163684	-0.3045153	-0.0993801	-0.1438072	-0.4000393	-0.1802242	-0.1840007	-0.0914727	-0.1313399
Uprosertib_2106	-0.4323998	-0.1941407	-0.2765995	-0.1223893	-0.1564509	-0.208142	-0.1597125	-0.3693683	-0.2508607	-0.0771707	0.18428234
LJI308_2107	-0.2248029	0.179973	0.02730527	-0.1093194	-0.0108698	0.1732744	0.15445961	-0.4745482	-0.5051213	-0.0678281	0.33279747
AZ6102_2109	-0.1439468	-0.0463563	-0.0567297	-0.1687269	-0.0791151	-0.0706682	-0.1617213	-0.3958934	-0.2830359	0.07165393	0.22230006
GSK591_2110	-0.1047585	0.23308729	0.11584498	0.0453997	-0.057185	0.19242265	0.3639323	-0.3027011	-0.2828613	0.08409599	0.36482792
AZD6482_2169	0.1335602	0.31973069	0.2051529	-0.0350309	0.28809883	-0.047246	0.06377273	-0.4988251	-0.4600697	0.23838077	0.49887883
AT13148_2170	-0.4732921	-0.0847364	-0.2506972	-0.1472667	-0.2184197	0.04912345	0.1059927	-0.3369491	-0.2952198	-0.2115445	0.15234922
BMS-754807_2171	-0.0735246	0.0045091	0.00690945	-0.0992627	0.05972144	-0.1594055	-0.104712	-0.5460462	-0.5420622	0.15045193	0.44601174
JQ1_2172	-0.1416749	0.08475792	0.05159152	-0.0927692	0.02799339	-0.0790296	0.00376177	-0.5264319	-0.6052987	0.03267593	0.41362064

The significance between the IC50 of various drugs and PCDI

	CHEK2	KRT18	GAPDH	MMP1	CHRNA5	TMPRSS4	ITGB4	CD79A	CTLA4	RRM2	PCDI
Camptothecin_1003	0.53023248	1.2838E-06	0.38838555	0.17461243	0.85892083	0.09991109	0.00012618	3.0483E-14	2.3251E-22	0.70664348	4.8209E-08
Cisplatin_1005	2.7933E-12	0.00159449	0.00982727	1.256E-06	0.00173851	0.00635752	1.2617E-07	2.4003E-19	1.9743E-29	4.7351E-10	0.0040071
Cytarabine_1006	0.00525899	0.02309885	0.88953205	0.19268493	8.1713E-05	0.15349332	2.3192E-05	4.4713E-08	1.69E-12	0.39125123	5.6675E-05
Docetaxel_1007	3.0653E-18	0.00033277	0	3.0998E-08	2.5473E-12	0.14226465	0.49990631	0.00320827	0.11668918	1.1325E-22	3.5973E-09
Gefitinib_1010	1.8415E-14	3.6641E-05	6.696E-06	0.05980098	0.00040521	0.37117301	5.5485E-10	0.22826625	0.09728091	7.4893E-07	0.00031481
Navitoclax_1011	4.3591E-24	0.00085305	2.4957E-06	0.02935191	3.2991E-07	0.00193629	0.14602671	1.3152E-19	1.6873E-34	5.3741E-09	0.00046042
Vorinostat_1012	8.2876E-07	0.00449559	0.01852351	0.30643769	0.02178616	0.00042457	6.1838E-10	1.6507E-14	9.1161E-14	0.72297258	2.9807E-15
Nilotinib_1013	1.2619E-18	0.35541683	0.00229812	0.51620624	1.3567E-05	0.11942432	4.4453E-06	3.2276E-16	2.248E-23	0.00100987	3.2108E-10
Olaparib_1017	0.18614203	2.4227E-12	1.2726E-05	0.35745462	0.65732898	1.2496E-05	2.6686E-11	1.1459E-17	1.673E-14	0.01890815	8.6606E-18
Axitinib_1021	1.4371E-22	2.6172E-06	1.4424E-09	1.498E-05	0.00779123	6.8197E-10	1.0302E-06	8.7067E-31	1.3343E-41	6.046E-05	5.5297E-07
AZD7762_1022	8.0384E-12	0.73027237	3.5416E-11	3.0706E-09	0.12223362	0.00292435	0.50101108	6.6472E-10	1.3523E-21	1.8694E-22	0.00154854
SB216763_1025	0.00382339	4.9849E-07	2.9202E-05	0.00110047	7.3679E-10	0.62358576	0.16512202	2.4256E-42	2.6898E-57	4.8512E-07	1.6474E-29
KU-55933_1030	0.2811302	0.0001213	0.79492098	6.0822E-14	0.00017034	0.46980485	0.11394031	1.3402E-32	3.4978E-28	0.37650698	3.7817E-05
Afatinib_1032	1.1623E-15	6.0755E-11	2.184E-10	0.00431416	0.31078379	3.7161E-11	5.8386E-27	0.03074288	0.01660063	0.00015764	0.00494071
PLX-4720_1036	3.2656E-12	0.02057553	4.8192E-08	1.3209E-10	0.32012543	0.00093106	5.1439E-08	8.1533E-26	4.233E-29	0.00354575	0.01041358
NU7441_1038	0.00110028	5.5603E-13	1.346E-05	6.3959E-06	8.0964E-07	0.00695642	0.06309129	5.8207E-26	2.7523E-17	0.00042513	1.3597E-14
Doramapimod_1042	0.00014895	7.5856E-16	0	0.00668561	2.533E-07	0.88890081	2.1569E-07	2.0538E-27	3.5638E-15	3.0362E-29	2.1758E-75
Nutlin-3a (-)_1047	8.7952E-09	4.0576E-10	1.164E-12	0.54559557	0.01988008	0.03834729	0.06173269	8.1469E-13	0.0002294	8.4762E-24	1.056E-23
PD173074_1049	3.4013E-15	0.71827759	0.01419757	0.00208995	0.00022089	0.31698636	0.97483045	1.1375E-26	4.5693E-27	0.00713758	2.5331E-08
ZM447439_1050	7.3981E-19	4.0415E-05	1.5775E-08	8.9327E-08	0.22990089	4.2566E-09	1.5015E-10	1.3263E-29	1.118E-42	3.4547E-07	0.00228066
RO-3306_1052	0.00024322	0.00747318	0.90315071	0.0035591	0.7524038	0.40063398	0.88806876	3.7076E-19	9.6971E-21	0.37536099	1.4484E-10
MK-2206_1053	7.9979E-14	0.00026556	1.7086E-05	0.0423743	0.00554096	5.1199E-07	0.00019792	5.9149E-29	1.1474E-13	0.54922312	9.0842E-12
Palbociclib_1054	6.6635E-06	0.00018089	5.9826E-06	0.00832663	0.00272638	4.7183E-05	0.00610367	1.2544E-16	2.5834E-14	0.2938606	6.2341E-07
Dactolisib_1057	0.00010744	0.00195855	7.299E-05	0.00938393	0.00458222	0.28776834	0.05282927	3.1091E-13	1.7327E-06	0.42059287	3.7891E-05
AZD8055_1059	6.0105E-08	0.0281086	1.9517E-07	8.785E-06	0.78765818	0.01842995	0.00869096	3.2301E-47	8.2883E-53	0.03086279	3.9576E-12
Dasatinib_1079	8.9622E-07	0.00012251	7.2386E-16	6.1031E-21	0.07349512	0.13156609	3.7528E-18	8.8974E-15	4.1261E-23	1.6799E-13	7.9933E-06
Paclitaxel_1080	6.2524E-49	1.0095E-11	0	3.9769E-07	8.4082E-15	0.44826024	0.01172857	2.6114E-07	2.6445E-08	4.3468E-31	2.7981E-05
Rapamycin_1084	1.7876E-42	2.9841E-11	0	0.00058578	5.039E-11	0.05800007	0.05799668	1.2747E-19	3.193E-19	1.7348E-10	0.02072173
Sorafenib_1085	1.1148E-18	0.20204102	0.00432329	0.58845281	5.5626E-10	0.71483975	0.00411447	1.1639E-13	1.475E-09	0.01975103	2.3297E-08
BI-2536_1086	0.54277579	6.424E-10	7.505E-05	0.13961886	0.0525074	3.4598E-14	3.0861E-18	3.741E-21	9.2385E-28	0.01167651	1.1493E-07
Irinotecan_1088	0.09274116	5.7434E-06	0.12603915	0.24077862	0.57359173	0.41934063	4.4963E-05	4.4546E-18	1.9814E-20	0.43712841	8.8351E-13
Oxaliplatin_1089	0.04748363	0.00438021	0.36805714	0.84514431	0.08233991	0.98595749	8.4066E-05	2.5437E-16	2.8108E-14	0.15325736	5.5808E-12
BMS-536924_1091	0.00602384	2.7647E-11	1.9297E-13	4.4333E-10	0.00022694	0.00319002	1.2683E-18	4.4541E-08	0.00477685	0.01918146	0.00325679
GSK1904529A_1093	1.1742E-25	0.19868753	0.00333506	0.20153886	1.3077E-11	0.07448675	7.1493E-06	6.4682E-07	4.3871E-05	1.6336E-05	0.00131957
PF-4708671_1129	1.1874E-13	2.0087E-08	2.6845E-08	0.00020091	0.33592782	9.9343E-13	1.9014E-11	4.7692E-39	8.5885E-48	0.04799291	8.915E-09
PRIMA-1MET_1131	0.07553303	2.2003E-14	4.5412E-11	0.98302466	4.0499E-12	3.0531E-05	0.00504083	1.1874E-20	1.3733E-35	3.8834E-07	5.4356E-30
Erlotinib_1168	0.00180603	6.2467E-07	0.00273006	0.1614776	0.11415027	2.183E-08	0.0374427	0.00114006	3.9453E-05	0.57898944	0.03989189
Niraparib_1177	0.10554853	6.7064E-14	4.1095E-11	0.22649756	0.10200906	0.00059285	7.8916E-09	1.7456E-15	2.4886E-16	1.4975E-11	1.0203E-31
MK-1775_1179	4.8463E-46	2.2173E-15	0	6.9786E-08	2.741E-18	0.10141821	4.1653E-06	4.2742E-09	6.4228E-21	1.1945E-47	3.0329E-08
Dinaciclib_1180	9.7459E-17	0.02865516	1.6633E-05	3.1391E-06	0.00819387	0.51391265	0.02593175	7.6331E-22	2.9971E-18	7.2665E-05	0.0200251

Gemcitabine_1190	0.14924186	0.00073803	0.4057808	0.00086501	0.85517803	0.17714123	0.09330767	3.4729E-15	1.0472E-28	0.00903946	0.00127588
GSK269962A_1192	2.8179E-09	0.00235321	0.00631782	0.00037466	0.62381227	2.4347E-06	0.00016947	1.4041E-44	3.1955E-36	0.49269129	1.6384E-17
SB505124_1194	9.7418E-39	2.0006E-25	0	7.4487E-12	0.00016192	1.0207E-07	7.4081E-24	1.1678E-17	1.0833E-40	2.8506E-39	7.6973E-06
Tamoxifen_1199	0.00611335	0.00028336	0.59501553	0.15763205	0.00043668	8.9273E-05	2.8333E-12	5.9855E-07	0.09078776	0.73584135	1.9878E-06
Fulvestrant_1200	2.6858E-14	0.00312065	0.56060065	0.63404565	0.01297709	0.01705649	8.3608E-07	4.1446E-19	6.779E-21	0.08231102	9.8499E-13
EPZ004777_1237	5.5199E-13	0.00385866	0.77850655	0.03797253	0.09347494	0.12356254	0.0176885	2.0029E-33	5.9666E-36	0.03107555	6.9599E-13
YK-4-279_1239	8.3547E-28	7.8096E-08	0	1.9852E-09	2.9339E-10	0.18281709	8.6425E-05	1.2362E-15	4.9458E-22	1.0497E-21	0.0315921
Daporinad_1248	0.00129464	6.6092E-06	7.9136E-05	0.45121711	0.07160164	0.20326459	7.5955E-08	0.0015842	0.0278607	0.96224989	3.1627E-06
BMS-345541_1249	1.1784E-36	7.5675E-22	0	0.00023783	6.6341E-18	9.3028E-05	6.183E-12	4.0186E-08	1.2492E-13	2.0847E-19	0.00863186
Talazoparib_1259	0.01355775	0.00096851	0.14146376	0.28439507	0.00096828	0.16027695	2.2298E-06	3.1139E-15	2.4293E-14	0.16718188	3.0501E-14
XAV939_1268	1.1656E-05	0.70452582	0.01483071	3.2994E-06	0.46489633	0.02634755	0.03984411	3.1935E-21	2.8328E-31	0.00113501	0.01569559
Trametinib_1372	4.3147E-18	0.69250344	0.70640438	7.3066E-06	4.4257E-07	0.03217484	1.8457E-19	0.12966863	0.18728613	2.1422E-06	0.0022825
Dabrafenib_1373	2.1409E-06	0.28266294	0.000465	2.0402E-05	7.99E-05	0.38787224	0.78806182	1.4696E-23	1.4349E-16	0.00663136	0.0008339
Temozolomide_1375	4.2664E-10	0.14970648	0.03940797	0.00871219	0.00513239	0.07463535	0.05082858	1.7649E-22	1.0237E-22	1.2655E-06	0.00222668
AZD5438_1401	2.1794E-06	0.80703694	0.02677465	8.355E-07	0.04248426	0.90051882	0.57890527	5.322E-23	1.8334E-17	0.05576117	0.00012853
IAP_5620_1428	0.0291249	6.8501E-10	0.00875792	0.38646361	0.94981272	3.6894E-13	9.3749E-22	0.00595951	0.01353431	0.13831447	0.00211879
AZD2014_1441	0.72417228	0.04021545	0.01772064	1.0974E-07	0.78252173	0.02135671	2.9503E-12	1.6648E-18	4.7664E-06	0.00263103	0.00033593
AZD1208_1449	0.00132852	4.1755E-08	0.00030653	0.6216019	0.03267702	0.00435518	3.6946E-08	1.2264E-19	1.0333E-18	0.21655636	4.1392E-21
AZD1332_1463	0.00026363	0.03523158	0.00181488	2.3706E-11	0.41664694	0.00637475	7.9331E-12	1.6016E-21	7.3875E-21	0.63575388	0.00390163
Ruxolitinib_1507	1.1252E-11	0.42125668	0.02879272	0.01225931	0.12866602	0.27990813	0.84633102	3.2632E-20	2.0589E-51	2.0121E-05	4.5598E-06
Linsitinib_1510	9.0416E-21	1.8972E-07	3.8861E-06	0.68231937	5.5489E-13	0.0540241	0.08428954	2.1549E-13	1.4569E-06	0.01773942	0.00325198
Epirubicin_1511	0.47533808	4.7856E-08	0.00057486	0.17205476	0.06044981	0.0001816	0.00091101	1.4675E-13	2.1368E-13	0.01021874	1.6197E-11
Cyclophosphamide_1	2.0629E-11	0.00042817	0.58656545	0.26150112	0.04774495	0.00415704	1.4937E-07	3.3251E-21	1.3119E-20	0.12748481	1.3261E-12
Sapitinib_1549	0.00731226	4.1335E-06	0.0039891	0.14068329	0.07178163	9.2237E-06	6.8437E-27	0.50112892	0.01937989	0.55610024	1.0063E-05
Uprosertib_1553	1.0548E-05	0.136365	0.01557852	0.10869043	0.73028405	8.2315E-05	1.0777E-06	1.2674E-15	7.279E-07	0.00566791	8.8256E-10
Lapatinib_1558	5.4389E-35	2.1686E-16	0	0.00367222	1.0083E-12	0.00382434	1.0683E-07	0.00066118	0.00348104	1.0014E-15	5.9745E-05
EPZ5676_1563	2.1822E-08	3.3052E-05	0.76758723	0.28069023	0.05950645	0.00075972	6.7235E-05	5.4535E-19	4.1234E-26	0.00871399	3.0276E-09
SCH772984_1564	1.755E-10	0.72551487	0.48463161	1.1906E-05	0.00683888	0.28829851	4.859E-12	0.01179683	0.03384175	0.03245019	6.9026E-07
IWP-2_1576	2.1334E-20	0.47900517	4.5403E-06	0.00587974	5.9208E-06	0.19357055	0.49118236	3.4175E-22	8.1094E-38	9.0123E-10	0.004836
Leflunomide_1578	7.62E-07	0.30187936	0.96408567	0.73924745	0.33478535	0.46451165	0.04322469	5.1363E-18	3.6777E-19	0.90743093	6.6162E-12
Entinostat_1593	0.18347043	4.6756E-11	0.1149201	0.0326473	0.80187762	1.0238E-08	2.2488E-10	3.2752E-14	1.3219E-21	0.14274461	2.8053E-06
LGK974_1598	1.4006E-16	0.01573202	0.00135343	0.60984127	1.4543E-11	0.01351815	0.03691096	2.8902E-14	5.7727E-10	8.1924E-05	0.00046812
WZ4003_1614	0.07365228	0.35974899	0.08019108	3.0656E-07	0.02422346	0.67396753	0.01035435	6.1569E-39	1.8515E-33	0.10940662	4.3944E-10
CZC24832_1615	2.1536E-09	0.04997484	0.01393909	4.5106E-06	0.48696726	0.37946062	0.75042716	1.2217E-42	2.3808E-62	0.00117162	7.6739E-11
GSK2606414_1618	5.1217E-10	0.00076732	5.3748E-09	2.9203E-07	0.11004354	0.0022987	3.2815E-09	9.6553E-22	1.2211E-28	0.00195266	0.0092544
PFI3_1620	3.7522E-11	0.00209424	0.28489875	0.00153788	0.94292363	0.00486145	0.02583315	6.7518E-30	3.2454E-46	0.00051461	9.9716E-10
PCI-34051_1621	0.3168764	5.1669E-11	9.0789E-09	0.36778891	0.10831493	3.5732E-06	1.3007E-07	1.1021E-29	1.2782E-27	0.00014519	1.1543E-31
Wnt-C59_1622	6.2314E-26	0.01179742	0.0005071	0.95071847	2.3509E-08	0.63669651	0.03285072	4.1636E-21	7.8236E-22	0.00074669	1.6364E-09
I-BET-762_1624	0.34331695	6.3335E-07	0.13256425	9.1743E-06	0.00683309	1.2E-07	0.25414029	1.3582E-16	2.9126E-17	0.96196536	0.00042639
RVX-208_1625	4.3919E-09	0.09057586	7.6696E-06	5.7312E-08	0.01644241	0.00356644	0.08559422	5.3153E-31	3.0423E-40	1.0391E-07	0.00353555
OTX015_1626	0.00537366	3.1047E-07	0.42797038	0.00016303	1.4424E-05	9.8852E-12	0.82590986	5.2023E-08	1.7213E-06	0.0229762	0.01368635
GSK343_1627	0.00116759	2.6255E-08	0.08316857	0.01809039	0.20709567	0.00040967	0.00295802	4.9239E-32	2.1956E-42	0.88792336	3.2889E-17
ML323_1629	7.786E-39	1.0063E-08	5.3753E-12	0.78909741	3.9453E-23	0.01449884	0.04322914	1.255E-10	3.6685E-08	5.5826E-10	0.02225273
PRT062607_1631	0.0121264	0.95080008	0.112339	5.6423E-05	0.06421355	0.02526491	1.7819E-06	8.1174E-44	7.8825E-47	0.14355939	3.7547E-12
Ribociclib_1632	6.4945E-07	0.00265265	1.7348E-05	4.2549E-05	0.88344254	1.0009E-07	3.1744E-05	1.1012E-55	9.3799E-74	0.19539448	1.6071E-14
AGI-6780_1634	6.7527E-08	0.00012045	0.2013036	0.74886962	0.00148706	0.00545217	3.4027E-12	5.3107E-16	7.4291E-13	0.29920479	3.1971E-13
Picolinici-acid_1635	0.00293518	1.6964E-06	0.17418304	0.03929699	0.53716194	0.00912302	0.00012838	3.0342E-29	4.7615E-33	0.83808729	8.3985E-16
AZD5153_1706	0.13277871	2.5925E-05	0.02117918	0.15160817	0.01384743	2.8651E-08	0.26349483	1.9277E-14	1.4897E-09	0.00012367	1.6491E-09
CDK9_5576_1708	6.4949E-06	0.32060927	0.19775574	0.00011326	0.00240009	0.22460769	0.24807492	7.3556E-19	4.9375E-13	0.75348059	2.5927E-06
CDK9_5038_1709	2.6513E-08	0.51848114	0.98199425	0.00369557	0.09840624	0.46996997	0.29230931	1.2626E-22	1.1073E-11	0.64347991	3.0237E-08
ERK_2440_1713	2.4141E-15	0.01076075	0.2352385	3.4405E-07	2.0086E-14	0.57007628	2.676E-11	1.0226E-07	4.6339E-14	2.0799E-09	0.00045994
ERK_6604_1714	4.9352E-10	0.43985051	0.17058242	1.5146E-09	0.00807856	0.80119269	6.1095E-11	0.15008949	0.31283661	0.10852645	4.5402E-08
IRAK4_4710_1716	0.00398061	0.00041451	0.13363673	0.68113007	0.00883746	0.00351503	1.4735E-06	1.6407E-10	4.5472E-07	0.88123777	1.0138E-07
JAK1_8709_1718	0.36717885	0.00043539	2.6116E-05	0.43123824	0.65338115	0.01422825	0.00159314	1.1279E-16	1.5553E-09	5.5004E-11	3.1927E-24
AZD5991_1720	7.0093E-12	0.00086666	0.68247557	0.15105437	0.00048628	1.414E-05	3.094E-13	1.1668E-10	2.7647E-08	0.09720077	2.9168E-11
PAK_5339_1730	3.0161E-20	0.00628673	5.8121E-06	0.00538231	6.3575E-05	0.69283735	0.05887023	3.1477E-30	1.1171E-24	0.00052494	0.00013016
TAF1_5496_1732	0.02321311	0.00042882	3.6559E-07	0.00022536	1.9446E-07	0.52999876	8.7429E-19	0.00199047	0.4395359	0.00151958	5.5317E-16
JAK_8517_1739	0.80844392	0.00022465	0.14410343	7.4858E-07	0.00671213	0.18387411	0.00437473	6.9365E-14	1.306E-12	0.16739867	0.00105105
AZD4547_1786	1.5732E-07	0.17868004	0.23298631	0.35340065	7.8407E-12	0.0459191	2.4822E-08	7.5247E-10	0.0472502	0.10377785	0.00036234
Zoledronate_1802	1.8368E-08	0.02662519	0.8282037	0.31769319	0.06172452	0.12237196	0.02636949	2.6145E-22	5.19E-27	0.71902264	5.8943E-16
Oxaliplatin_1806	0.61905763	1.2803E-06	5.6119E-05	0.22215026	0.53945602	0.12670946	0.00015014	3.2072E-14	4.4344E-10	1.3266E-07	8.8522E-19
Carmustine_1807	2.7171E-13	0.1253072	0.49145667	0.98546562	1.3751E-05	0.00869902	0.00038161	1.0968E-09	1.3625E-16	0.0011568	5.3637E-05
Topotecan_1808	0.67098808	2.5204E-07	0.06719792	0.48597527	0.33473495	0.05908811	0.00017865	9.1123E-16	4.9398E-31	0.76020209	7.2262E-11
Teniposide_1809	0.01299894	0.00057902	0.35558293	0.03039387	0.5527971	0.01224603	0.00300909	1.0075E-23	1.7103E-42	0.01434128	6.253E-08
Mitoxantrone_1810	9.7929E-08	8.4003E-24	2.7393E-10	0.41715692	0.00049094	7.0439E-08	1.0605E-09	9.0803E-10	6.503E-14	5.493E-07	2.1568E-15
Dactinomycin_1811	0.0102539	0.21344101	0.05279011	0.09547614	0.2692845	0.28343607	0.95265098	6.1694E-08	2.2545E-13	0.49015089	0.00855405
Fludarabine_1813	0.14161179	0.0001198	0.22336262	1.3063E-06	0.01267524	0.01160253	0.52436545	2.8455E-20	5.3173E-46	0.19561128	4.659E-06
Nelarabine_1814	1.7263E-05	0.00052022	0.21997327	0.06593973	0.46990451	0.05955788	0.13028643	1.0324E-19	1.9859E-32	0.12925881	1.5188E-09
Fulvestrant_1816	2.596E-35	4.506E-05	1.1203E-07	0.70170489	5.0293E-17	0.75049351	0.02646604	2.4564E-10	1.0041E-13	1.5693E-10	0.03020828
Docetaxel_1819	2.0184E-18	5.1923E-06	0	1.5723E-09	6.4975E-09	0.08934833	0.01315159	9.301E-10	9.5655E-08	1.3602E-21	5.8882E-06
Dihydrorotenone_182	1.6492E-14	0.01720616	0.53362784	0.00648556	4.4672E-17	0.06648028	6.6086E-08	0.01023186	0.41625812	0.24271403	9.9984E-05
Gallibiscoquinazole_1	2.1862E-32	0.01156572	1.2895E-07	0.08385349	3.8683E-12	0.43442758	0.07291763	3.7831E-17	6.0015E-24	1.0984E-12	0.00189703
Elephantin_1835	0.0399189	5.6835E-06	1.1756E-05	0.59898189	0.00037681	0.00080164	0.00778992	4.0852E-16	1.4716E-14	0.00117002	3.5304E-18
Sinularin_1838	1.1018E-09	0.38875492	0.0192892	0.00342872	0.01619064	0.56590238	0.0572653	0.00052954	0.00010428	0.29538825	2.5592E-10
Sabutoclax_1849	8.3529E-07	4.702E-05	0.20978164	0.30306283	0.30036048	4.5157E-05	1.2167E-07	1.3589E-15	3.5101E-16	0.703723	1.4466E-12
LY2109761_1852	0.00304684	0.00026979	0.03678638	0.50219546	0.2538448	0.77547062	0.09658943	3.2239E-17	3.8381E-20	0.46132717	5.4339E-14
OF-1_1853	0.01839731	0.00037377	3.6457E-07	2.085E-05	4.0613E-07	0.11449838	4.5041E-25	0.0125347	0.3403947	0.0001737	3.0484E-18
MN-64_1854	1.188E-16	0.98707371	0.01058551	0.00951588	0.07452716	0.35235032	0.22317923	8.2064E-12	7.3561E-20	4.2041E-05	0.00302315
KRAS (G12C) Inhibitor	2.2367E-20	0.28061241	0.09072861	0.84004762	1.4495E-07	0.64712957	0.00090587	2.4805E-10	2.7714E-09	0.02002351	3.4806E-07
Venetoclax_1909	0.00086633	9.1872E-08	0.0765424	0.03923241	0.39881845	0.00352108	1.4687E-09	9.3449E-27	7.7222E-23	0.74310916	2.6346E-16
ABT737_1910	0.01752404	1.1592E-10	4.8947E-05	0.92004244	0.82024721	0.00039717	2.7793E-09	7.5964E-15	2.9299E-18	0.9718971	2.0368E-13
Dactinomycin_1911	0.91437121	0.00031935	0.26136204	0.25240268	0.84063202	0.00060438	0.01038338	6.9854E-05	0.12900867	0.08074261	0.00355972
Afuresertib_1912	7.8534E-12	0.13633219	0.00717048	0.03333325	0.03304638	1.8285E-06	0.02195228	5.269E-17	1.1084E-05	0.24849719	1.0512E-08
AGI-5198_1913	9.3147E-06	1.4316E-06	0.59632597	0.31478094	0.06721038	0.0002137	8.9577E-11	7.5381E-20	1.5024E-18	0.14882114	2.3374E-11
AZD5363_1916	1.1962E-22	4.0386E-06	1.4047E-12	4.0094E-06	0.0009442	5.0558E-07	7.1874E-07	1.4041E-21	5.3741E-13	0.00438217	0.00817033
AZD6738_1917	1.5353E-31	6.0052E-11	0	7.269E-13	4.1226E-14	0.15522316	3.4457E-06	3.2881E-09	4.9693E-21	2.2732E-36	1.006E-07
AZD8186_1918	0.00538141	0.01626058	4.4935E-06	1.3872E-08	0.02632539	5.7586E-06	4.1009E-17	1.8858E-25	3.1004E-21	0.94487455	0.00085867
Osimertinib_1919	1.493E-26	1.0244E-18	1.2314E-16	0.00529951	1.2114E-05	4.5288E-08	9.6983E-21	0.00108217	3.694E-06	1.7974E-08	0.02668552
Ipatasertib_1924	1.1834E-30	3.529E-09	1.1644E-14	0.00082956	1.0627E-06	2.1976E-11	1.3978E-06	5.7459E-16	7.4671E-10	0.00065706	0.01423557
GNE-317_1926	0.02118651	0.00096672	0.00112298	0.00031359	0.17863814	0.0009406	3.0636E-05	6.6768E-25	2.8044E-08	0.00518715	8.367E-08
GSK2578215A_1927	4.2342E-26	0.25708222	0.0030299	0.123468	2.731E-06	0.00113382	7.8772E-09	1.0693E-23	1.4181E-27	4.4936E-07	2.1118E-08
I-BRD9_1928	6.4483E-21	0.05115513	7.8179E-08	0.01957985	1.5019E-05	0.0232494	0.46677479	7.6624E-26	3.9574E-28	0.00037842	9.6684E-08
MIRA-1_1931	4.8796E-22	0.38445599	0.21917544	0.41318105	0.00011687	0.05418302	0.00017066	3.6085E-25	4.3844E-32	0.00051097	8.0256E-11
NVP-ADW742_1932	1.7836E-08	7.399E-05	2.3523E-07	0.00118837	7.7147E-08	0.01581167	0.0001549	4.0485E-16	3.8585E-06	0.1774507	0.02158437
P22077_1933	1.5464E-38	0.00780852	1.1861E-07	0.64123388	1.417E-11	0.13403077	1.332E-05	7.4891E-10	7.7573E-14	2.7049E-11	0.00055802
Savolitinib_1936	0.00536632	0.08196456	6.6752E-05	3.6216E-05	0.00045834	8.2654E-06	0.00528472	0.00140888	5.9513E-07	3.4894E-12	0.01769191
UMI-77_1939	4.4685E-20	0.93160127	0.0022586	0.01021367	0.00019842	5.0278E-08	3.4658E-05	0.00025986	1.2878E-05	3.8153E-20	0.00884959
WIKI4_1940	3.1363E-24	2.3086E-10	1.2971E-18	2.0891E-06	6.7626E-05	0.16950926	2.3279E-09	2.3455E-05	8.9398E-17	1.2894E-26	1.1651E-07

Sepantronium bromic	4.9382E-09	0.0373864	0.01106374	0.31742316	0.16890738	1.907E-07	0.00083689	0.01330644	0.02049762	8.7881E-12	4.1734E-06
BIBR-1532_2043	0.00184583	2.9016E-12	2.5008E-06	0.2429644	0.46111779	2.1732E-07	4.0749E-15	8.7151E-13	2.3177E-08	0.02439071	1.8429E-18
Pyridostatin_2044	1.6011E-20	0.08383297	0.05677703	0.14691885	0.00031966	0.09925571	0.00217107	9.3878E-14	4.8756E-12	0.00184841	9.2397E-06
AMG-319_2045	1.0686E-05	1.0456E-07	0.97387122	0.00598302	0.03044222	0.03360316	0.01216232	1.6248E-35	1.0079E-56	0.05998792	1.7586E-13
VX-11e_2096	0.13912558	2.8008E-09	2.6077E-13	1.6393E-12	0.02410824	0.00106563	3.2637E-21	3.8931E-05	2.6525E-05	0.03797237	0.0031967
Uprosertib_2106	7.0727E-25	9.1079E-06	2.0248E-10	0.00541635	0.00036556	1.8952E-06	0.00027367	4.3049E-18	7.8501E-09	0.08018117	3.2636E-05
LJI308_2107	2.5319E-07	3.9927E-05	0.53627677	0.01305493	0.80561769	7.7305E-05	0.00043504	2.7877E-30	1.0638E-34	0.12422125	1.9003E-14
AZ6102_2109	0.0010536	0.29371754	0.19861473	0.00011941	0.07283613	0.10919464	0.00022834	9.0137E-21	6.0809E-11	0.10432811	4.8695E-07
GSK591_2110	0.01740124	8.7657E-08	0.00853337	0.3038041	0.19509893	1.096E-05	1.4232E-17	2.2507E-12	6.2545E-11	0.05649678	2.9992E-17
AZD6482_2169	0.00238774	1.0517E-13	2.8131E-06	0.42760976	2.6661E-11	0.28454098	0.14840498	9.4101E-34	2.4389E-28	4.3577E-08	6.0116E-33
AT13148_2170	4.144E-30	0.05463588	9.0452E-09	0.00080176	5.579E-07	0.26581515	0.01611528	3.8725E-15	8.1278E-12	1.2727E-06	0.00061493
BMS-754807_2171	0.09556608	0.91869341	0.87565887	0.02427675	0.17598853	0.00028129	0.01745142	2.3255E-41	1.1366E-40	0.00061363	6.5701E-26
JQ1_2172	0.001266	0.05457436	0.24242259	0.0353181	0.52617769	0.07314683	0.93213332	4.6992E-38	8.5352E-53	0.45934211	3.626E-22
