# Supplementary material for: Machine learning reveals diverse cell death patterns in lung adenocarcinoma prognosis and therapy
Source: NPJ Precis Oncol. 2024 Feb 26;8:49. doi: 10.1038/s41698-024-00538-5 (PMC10897292; doi:10.1038/s41698-024-00538-5)
Supplement: Supplementary file 2 — REPORTING SUMMARY [file 41698_2024_538_MOESM2_ESM.pdf]

nature portfolio

Corresponding author(s): Jie Huang

Last updated by author(s): 2024-1-25

Reporting Summary

Nature Portfolio wishes to improve the reproducibility of the work that we publish. This form provides structure for consistency and transparency in reporting. For further information on Nature Portfolio policies, see our Editorial Policies and the Editorial Policy Checklist.

Statistics

For all statistical analyses, confirm that the following items are present in the figure legend, table legend, main text, or Methods section.

n/a	Confirmed	
☐	☒	The exact sample size (*n*) for each experimental group/condition, given as a discrete number and unit of measurement
☐	☒	A statement on whether measurements were taken from distinct samples or whether the same sample was measured repeatedly
☐	☒	The statistical test(s) used AND whether they are one- or two-sided<br>*Only common tests should be described solely by name; describe more complex techniques in the Methods section.*
☐	☒	A description of all covariates tested
☐	☒	A description of any assumptions or corrections, such as tests of normality and adjustment for multiple comparisons
☐	☒	A full description of the statistical parameters including central tendency (e.g. means) or other basic estimates (e.g. regression coefficient) AND variation (e.g. standard deviation) or associated estimates of uncertainty (e.g. confidence intervals)
☐	☒	For null hypothesis testing, the test statistic (e.g. *F*, *t*, *r*) with confidence intervals, effect sizes, degrees of freedom and *P* value noted<br>*Give P values as exact values whenever suitable.*
☐	☒	For Bayesian analysis, information on the choice of priors and Markov chain Monte Carlo settings
☐	☒	For hierarchical and complex designs, identification of the appropriate level for tests and full reporting of outcomes
☐	☒	Estimates of effect sizes (e.g. Cohen's *d*, Pearson's *r*), indicating how they were calculated

Our web collection on statistics for biologists contains articles on many of the points above.

Software and code

Policy information about availability of computer code

Data collection	RNA-seq data and corresponding clinicopathological information for LUAD samples were obtained from The Cancer Genome Atlas (TCGA) . In addition, clinicopathological information and genome-wide expression data for four other LUAD cohorts (GSE116959, GSE31210, GSE50081, and GSE72094) along with the immunotherapy cohort (GSE126044 and GSE78220) were obtained from the Gene Expression Omnibus (GEO) database . To compile the list of PCD genes, we gathered genes associated with 13 different PCD patterns from reputable scientific sources, including GSEA gene sets, KEGG, review articles, and manual compilation . After eliminating duplicate genes, a total of 2090 PCD-associated genes were included for subsequent analysis.
Data analysis	Statistical analyses were performed using R software (version 4.1.0). Continuous variables were reported as the standard error of the mean and compared using either the Student's t test or Wilcoxon rank sum test. Categorical data were assessed using the chi-square test. Statistical significance was defined as P< 0.05.

For manuscripts utilizing custom algorithms or software that are central to the research but not yet described in published literature, software must be made available to editors and reviewers. We strongly encourage code deposition in a community repository (e.g. GitHub). See the Nature Portfolio guidelines for submitting code & software for further information.

Data

Policy information about availability of data

All manuscripts must include a data availability statement. This statement should provide the following information, where applicable:

- Accession codes, unique identifiers, or web links for publicly available datasets
- A description of any restrictions on data availability
- For clinical datasets or third party data, please ensure that the statement adheres to our policy

The datasets used in this paper are available online

Research involving human participants, their data, or biological material

Policy information about studies with human participants or human data. See also policy information about sex, gender (identity/presentation), and sexual orientation and race, ethnicity and racism.

Reporting on sex and gender	N/A
Reporting on race, ethnicity, or other socially relevant groupings	N/A
Population characteristics	N/A
Recruitment	N/A
Ethics oversight	N/A

Note that full information on the approval of the study protocol must also be provided in the manuscript.

Field-specific reporting

Please select the one below that is the best fit for your research. If you are not sure, read the appropriate sections before making your selection.

☒ Life sciences ☐ Behavioural & social sciences ☐ Ecological, evolutionary & environmental sciences

For a reference copy of the document with all sections, see nature.com/documents/nr-reporting-summary-flat.pdf

Life sciences study design

All studies must disclose on these points even when the disclosure is negative.

Sample size	N/A
Data exclusions	None
Replication	none
Randomization	none
Blinding	none

Reporting for specific materials, systems and methods

We require information from authors about some types of materials, experimental systems and methods used in many studies. Here, indicate whether each material, system or method listed is relevant to your study. If you are not sure if a list item applies to your research, read the appropriate section before selecting a response.

Materials & experimental systems

n/a	Involved in the study	
☐	☒	Antibodies
☒	☐	Eukaryotic cell lines
☒	☐	Palaeontology and archaeology
☒	☐	Animals and other organisms
☒	☐	Clinical data
☒	☐	Dual use research of concern
☒	☐	Plants

Methods

n/a	Involved in the study	
☒	☐	ChIP-seq
☒	☐	Flow cytometry
☒	☐	MRI-based neuroimaging

Antibodies

Antibodies used	Antibodies against GPX4 (1: 5000, ab125066, Abcam), SLC7A11 (1: 1000, 26864-1- AP, Abcam), ACSL4(1: 50000, ab155282, Abcam), and GAPDH (1: 15000, 60004-I-Ig, Proteintech) were used as primary antibodies,antibodies against CHEK2 (rabbit, 1:100, Absin, Abs106836), RRM2 (rabbit, 1:100, Proteintech, 11661-1-AP), and GAPDH (mouse, 1:100, Proteintech, 60004-1-lg)
Validation	data provided in the manuscript

Plants

Seed stocks	none
Novel plant genotypes	none
Authentication	none
